# Supplementary material for: Synthetically engineered probiotic suppresses colorectal cancer via inhibition of Wnt/β-catenin signaling
Source: Cell Rep Med. 2026 Aug 18;7(8):102913. doi: 10.1016/j.xcrm.2026.102913 (PMC13522751; doi:10.1016/j.xcrm.2026.102913)
Supplement: Supplementary file 1 [file mmc1.pdf]

Cell Reports Medicine, Volume *7*

Supplemental information

Synthetically engineered probiotic suppresses colorectal cancer via inhibition of Wnt/β-catenin signaling

Yujie Sun (孙妤婕), Yu Zhao (赵宇), Kang Han (韩康), Wulong Hu, Guofan Peng, Shengen Yi, Jun Zhou (周俊), and Jin Hai Zheng (郑金海)

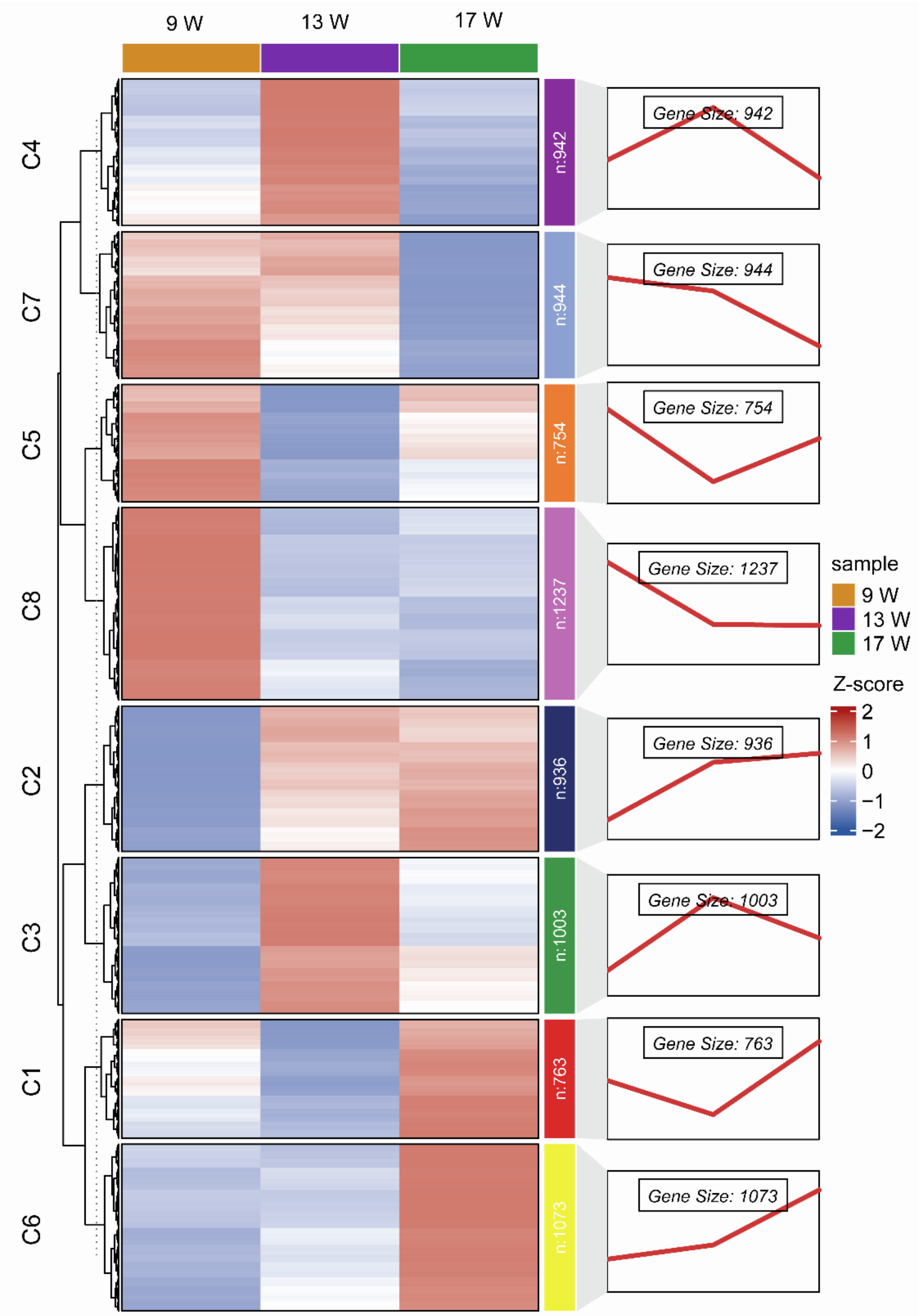

Figure S1. Heatmap cluster for visualizing clustered metabolite of *Apc*$^{Min/+}$ mice stools in time course. Related to Figure 1.

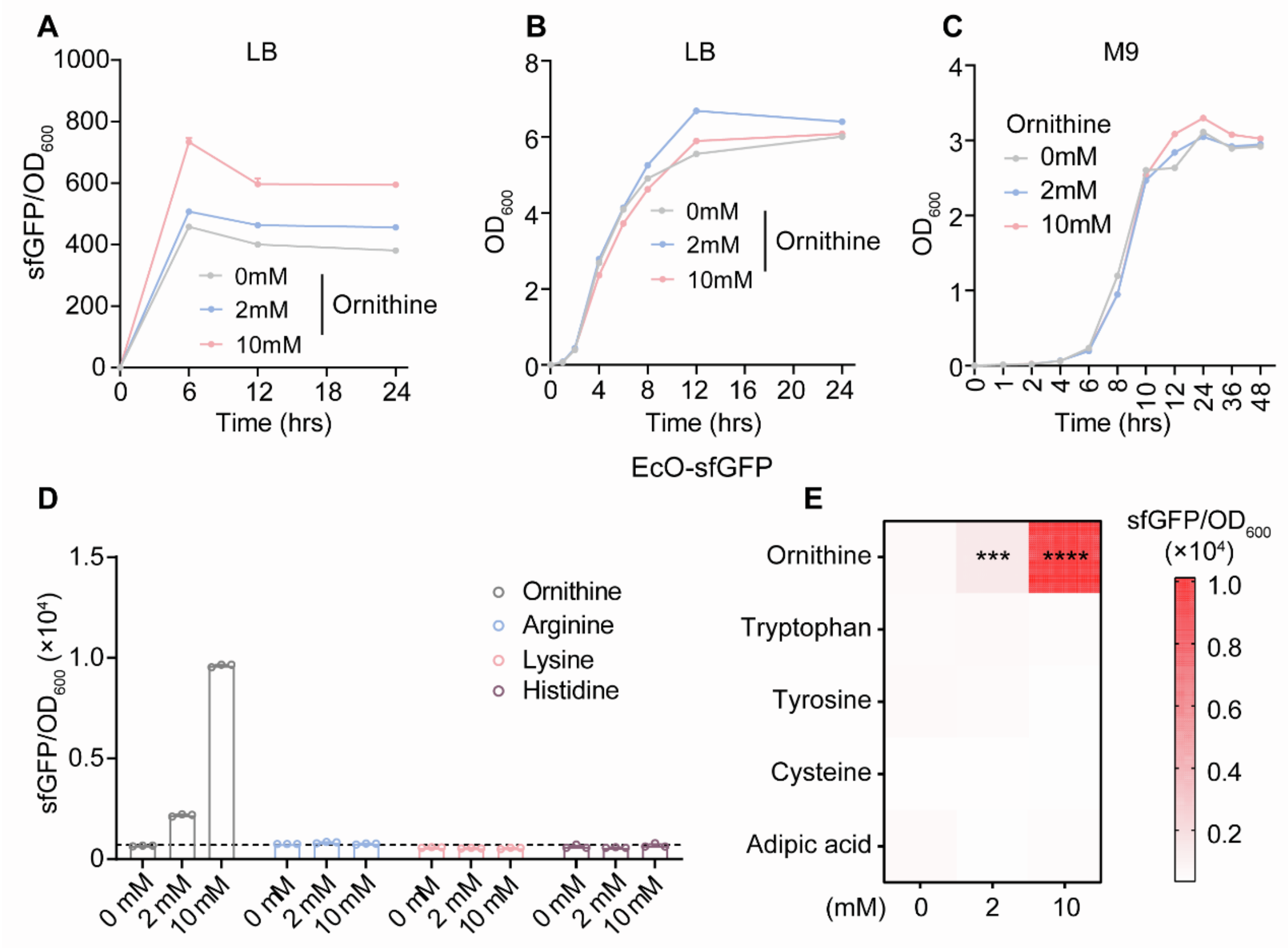

Figure S2. The profiling of EcO-sfGFP and its specificity *in vitro*. Related to Figure 1. (A) Fluorescence intensity of with ornithine responsive plasmid treated with 0, 2, 10 mM ornithine within 24 hours in LB medium. (B and C) Growth curve of engineered bacteria EcO-sfGFP with different ornithine concentrations in LB medium (B) and M9 medium (C). (D) Fluorescence intensity of EcO-sfGFP with arginine, lysine and histidine supplementation. (E) Heatmap of fluorescence intensity of EcO-sfGFP co-culture with tryptophan, tyrosine, cysteine and adipic acid. Statistical significance determined using an unpaired t test; *** $P \leq 0.001$, and **** $P \leq 0.0001$.

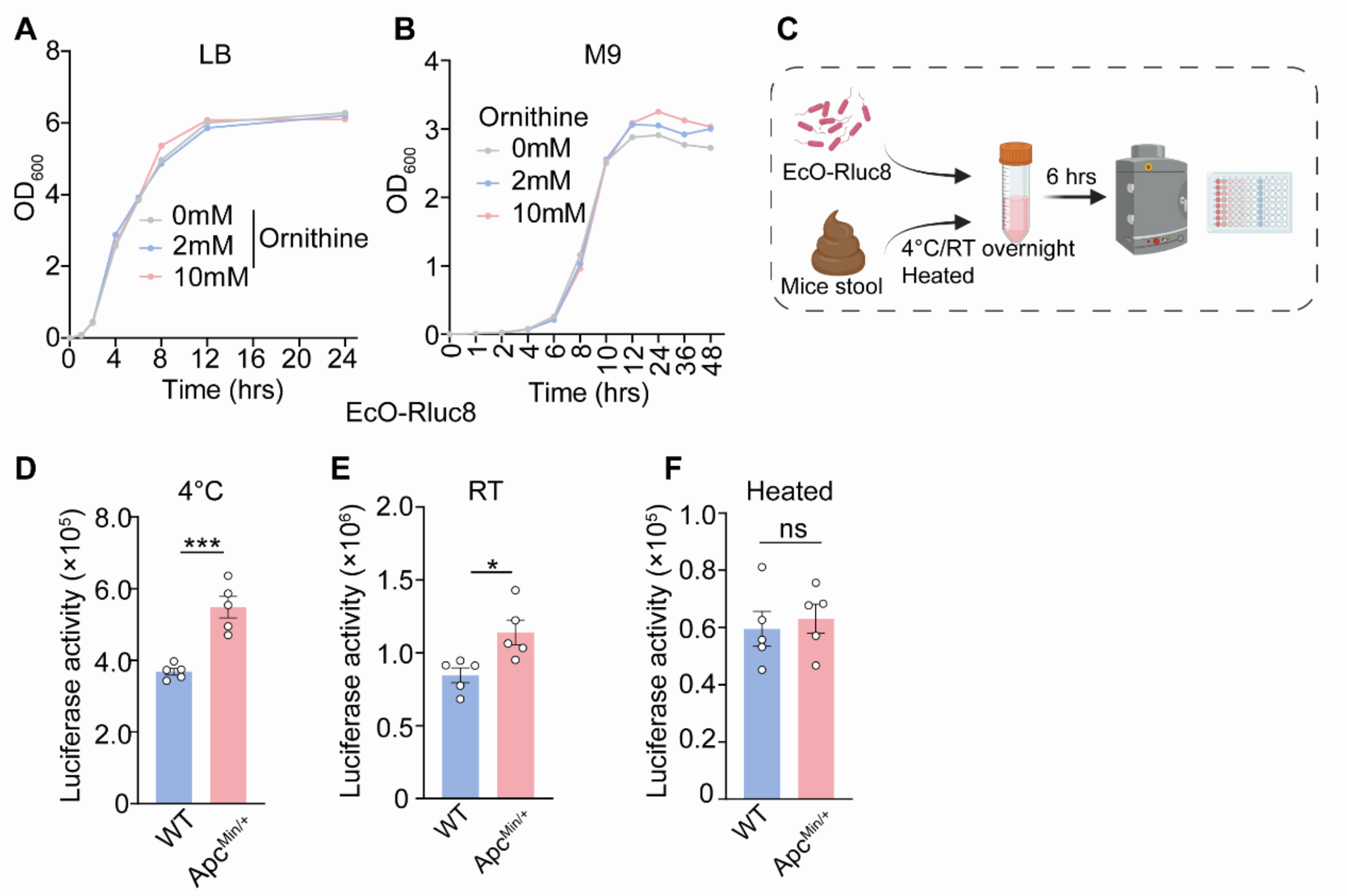

Figure S3. The profiling of EcO-Rluc8 with ornithine. Related to Figure 2. (A) Growth curve of engineered bacteria EcO-Rluc8 with different ornithine concentrations in LB medium. (B) Growth curve of engineered bacteria EcO-Rluc8 with different ornithine concentrations in M9 medium within 24 hours. (C) Schematic design of the efficiency of EcO-Rluc8. (D-F) Bioluminescence signals of EcO-Rluc8 exposure with stools deal with 4°C (D), room temperature (E) overnight and heated (F). Data are presented as mean ± SEM. Statistical significance determined using an unpaired t test; ns > 0.05, $*P \leq 0.05$ and $***\ P \leq 0.001$.

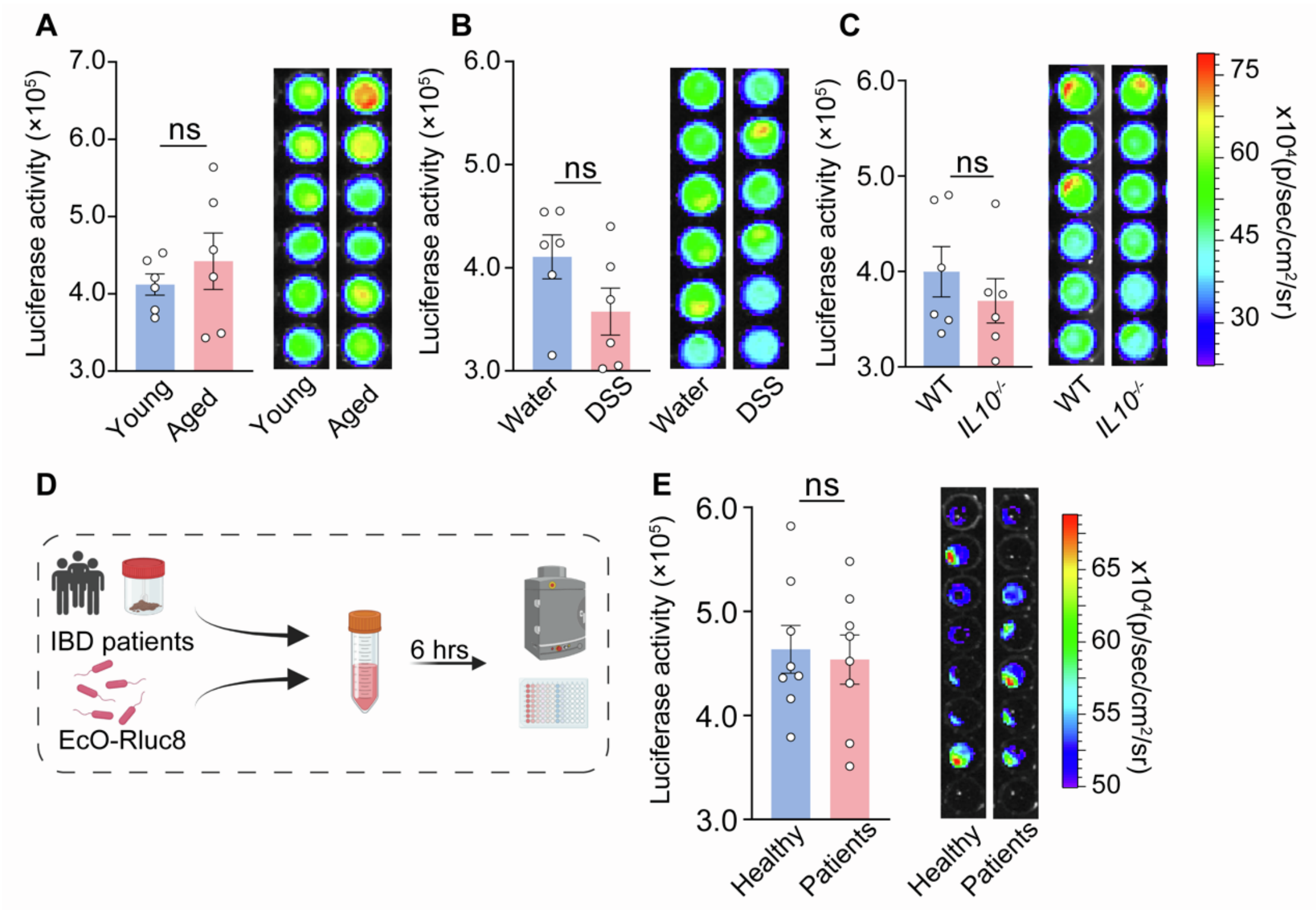

Figure S4. Specificity of EcO-Rluc8 in multiple intestinal disease models. Related to Figure 2. (A to C) Quantitative bioluminescence signals and images after co-incubating bacteria with stools from aged mice (A), DSS-treated (B), *IL10*$^{-/-}$ mice (C) and corresponding control groups (n = 6). (D) Schematic diagram of the efficiency of EcO-Rluc8 responsive to stools from IBD patients and healthy individuals. (E) Quantitative bioluminescence signals and images of co-culture EcO-Rluc8 with stools from IBD patients (n = 8). Data are presented as mean ± SEM. Statistical significance determined using an unpaired t test. ns, $P > 0.05$.

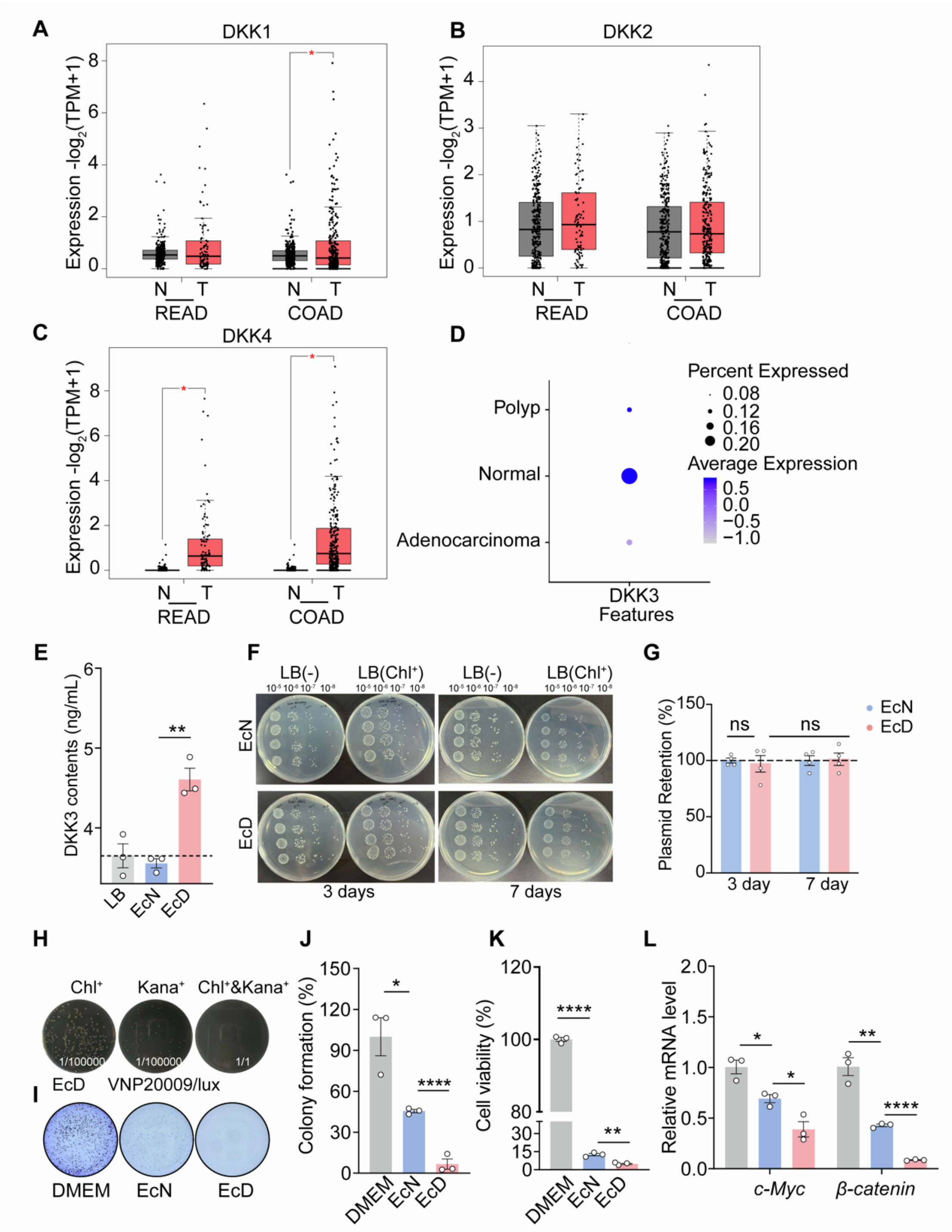

Figure S5. Expression of DKKs from the GEPIA database in colorectal cancer and the profiling of EcD *in vitro*. Related to Figure 3. (A to C) The mRNA level of DKK1 (A), DKK2 (B), and DKK4 (C) in colorectal cancer patients. READ (num(N)= 318, num(T) = 92), COAD (num(N) = 349, num(T) = 275). N, normal, T, tumor. (D) The expression of DKK3 in CRC patients within scRNA-seq analysis (GSE201349). (E) The contents of DKK3 in bacterial supernatant with 1×10^{8} CFU bacteria. (F and G) Plasmid retention ratio of EcN and EcD *in vitro*. (H) The potential ability of horizontal gene transfer of EcD. (I) Representative images with crystal violet staining of HT29 cells with DKK3 co-incubation. (J and K) Colony formation

ratio (J) and cell viability (K) of HT29 treated with DKK3. (L) Quantification of mRNA levels of HT29 treated with DKK3. Statistical significance determined using an unpaired t test; ns, $P > 0.05$, *$P \leq 0.05$, ** $P \leq 0.01$ and **** $P \leq 0.0001$.

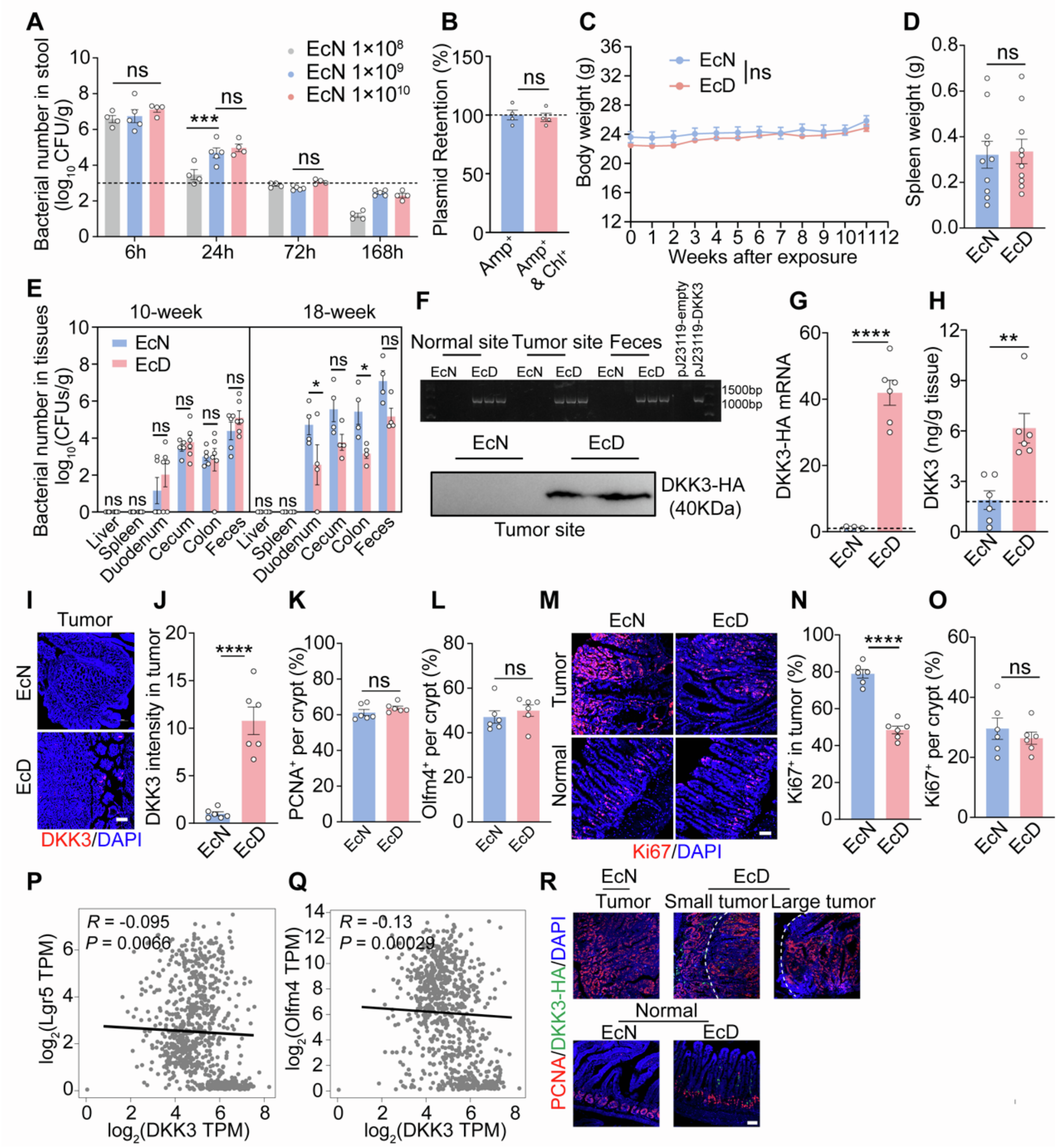

Figure S6. The stability of EcD *in vivo* and the effects of ISCs proliferation within EcD administration. Related to Figure 4. (A) Fecal colonization kinetics of EcN with single dose administration. (B) Plasmid stability of pJ23119-pelB-DKK3-HA in gut. (C and D) Body weight and spleen weight change in $Apc^{Min/+}$ mice with bacteria exposure. (E) Bacterial colonization after administration in 10- and 18-week-old $Apc^{Min/+}$ mice. (F) PCR and Western blot assays of engineered bacteria selected from related tissues. (G) The mRNA levels of DKK3-HA in tumors with EcD treatment. (H) Concentrations of DKK3 in tumor tissues. (I and J) Immunofluorescent staining (I) and signal intensity (J) of DKK3 in tumor tissues of small intestines. (K and L) Immunofluorescence signal quantification of PCNA (K) and Olfm4 (L) in crypts. (M to O) Immunofluorescent staining (M) and signal intensity with Ki67 in tumor tissues (N) and normal tissues (O) of small intestines. (P and Q) Correlation analysis between *DKK3* and *Lgr5* (P) and *Olfm4* (Q) in CRC patients using TCGA database. A tendency line was added based on the value of “*R*”. (R) Immunofluorescent

co-staining of PCNA and HA-tag in small intestines of *Apc*$^{Min/+}$ mice with EcD treatment. Data are presented as mean ± SEM. Statistical significance determined using an unpaired t test. ns, $P > 0.05$, *$P \leq 0.05$, ** $P \leq 0.01$ and **** $P \leq 0.0001$.

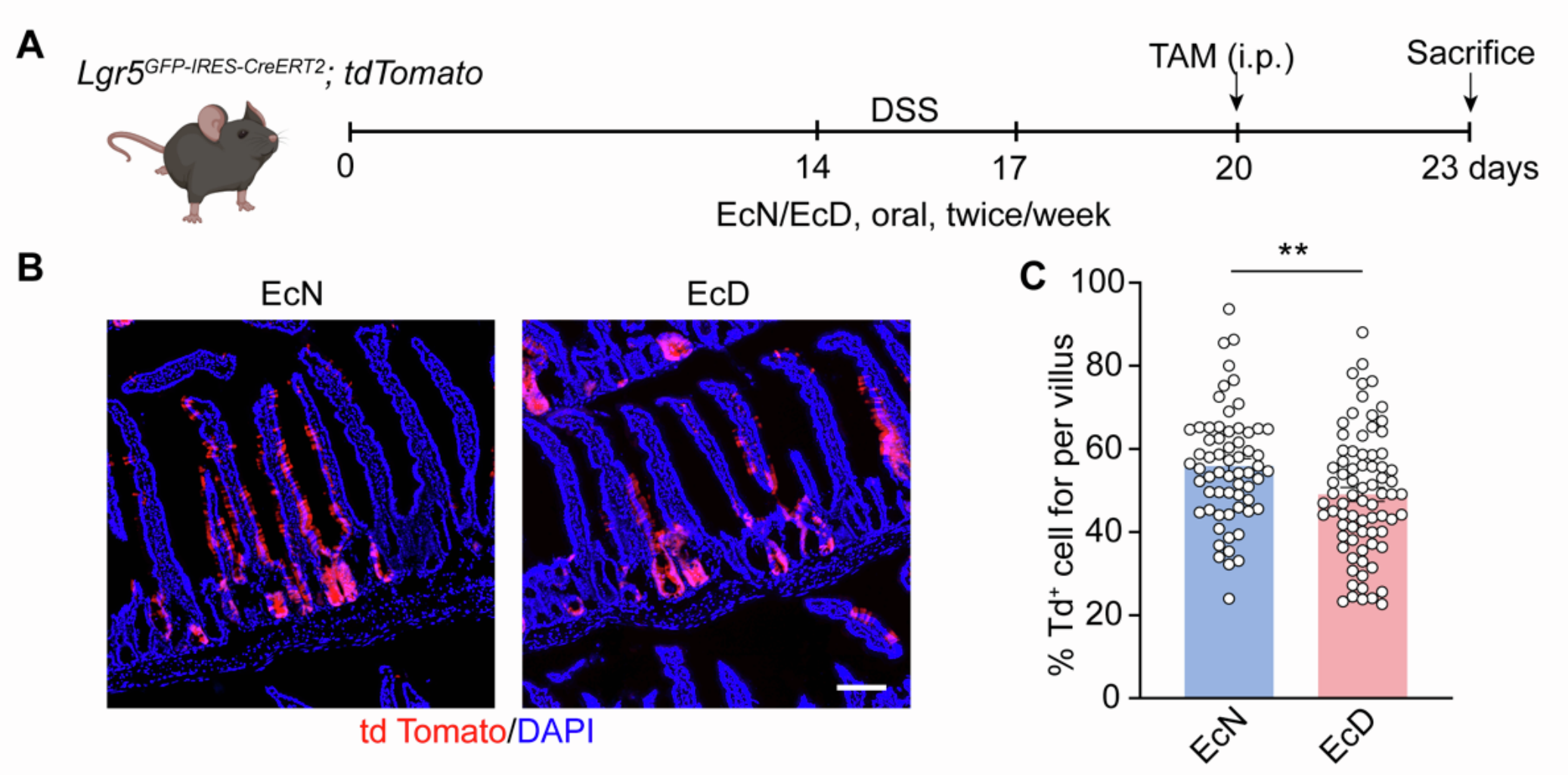

Figure S7. ISC proliferation in *Lgr5$^{GFP\text{-}CreERT2}$; tdTomato* mice with EcD treated. Related to Figure 4. (A) Experimental scheme of DSS and EcD treatment. (B) Representative images showing Lgr5-derived progeny (red) in small intestine after TAM treatment. (C) Quantification of tdTomato positive cell per villus with EcN and EcD treated. Data are presented as mean ± SEM. Statistical significance determined using an unpaired t test, ** $P \leq 0.01$.

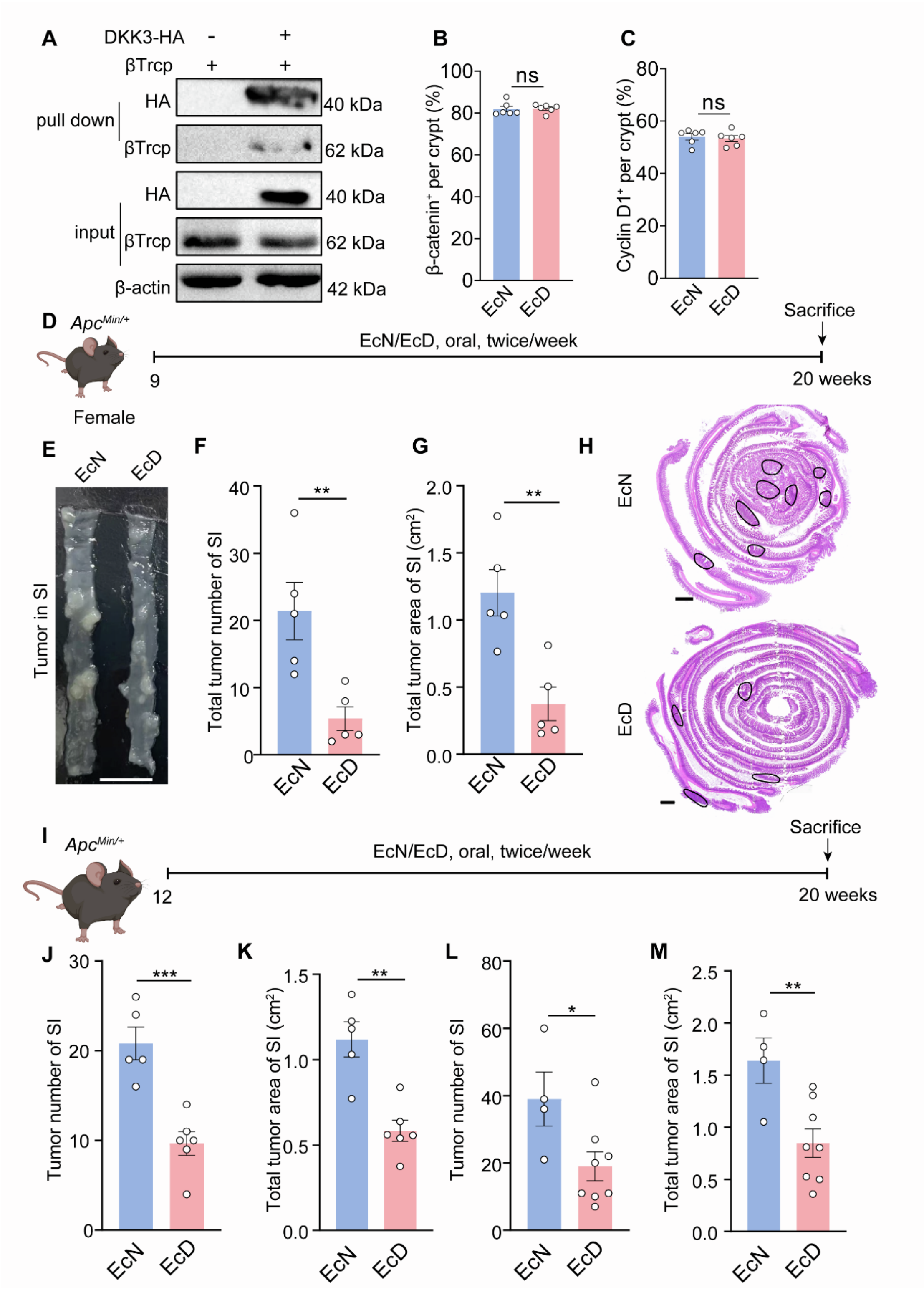

Figure S8. Effects of Wnt/β-catenin signaling within EcD administration. Related to Figure 5. (A) The interaction of DKK3-HA secreted from EcD with the βTrcp protein was validated by pull-down assay in HCT116 cells. (B and C) Immunofluorescent signal intensity analysis of β-catenin (B) and Cyclin D1 (C) in normal tissues of small intestines of *Apc*$^{Min/+}$ mice with EcD treatment. (D) Experimental design. The 9-week-old female *Apc*$^{Min/+}$ mice were randomly grouped

and oral administrated with EcN or EcD twice a week till the 20th week. The tumor numbers were counted and tumor tissues were collected. (E) At the 20th week, mice were sacrificed, and intestinal tissues were excised and counted, scale bar = 2 cm. (F and G) Analysis tumor numbers and total tumor areas in small intestine, n = 5. (H) Representative H&E-stained histological images of small intestines with EcN and EcD treated mice, scale bar = 1 mm. (I) Experimental design. The 12-week-old male and female $Apc^{Min/+}$ mice were divided randomly and oral administrated with EcN or EcD twice a week till the 20th week. The tumor numbers were counted. (J to M) At the 20th week, male (J and K) and female (L and M) mice were sacrificed, tumors from small intestine were counted and the total tumor areas were analyzed. Data are presented as mean ± SEM. Statistical significance determined using an unpaired t test. ns, $P > 0.05$, *$P \leq 0.05$, ** $P \leq 0.01$ and *** $P \leq 0.001$.

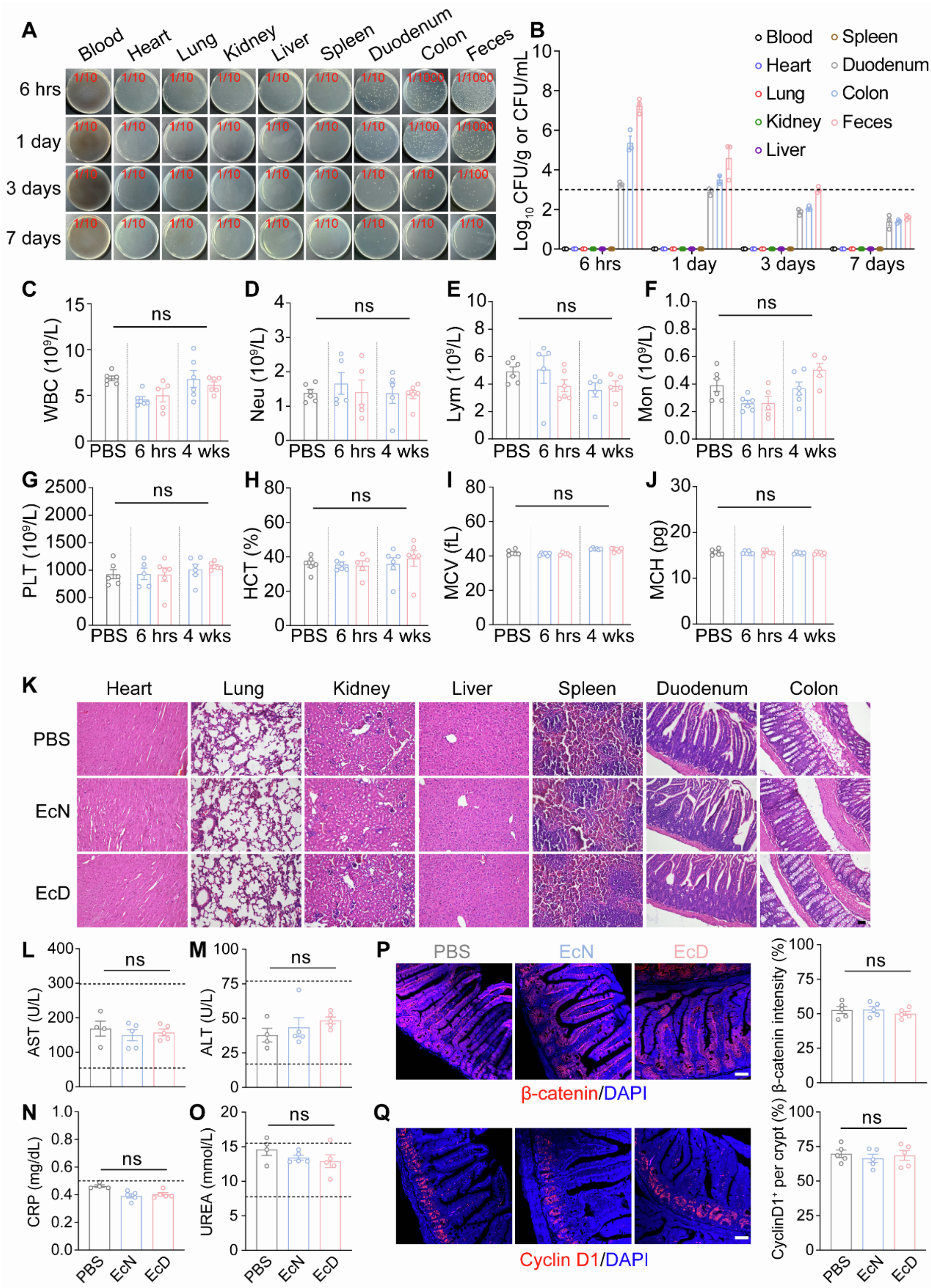

Figure S9. Biosafety assessment of EcD. Related to Figures 4 and 5. (A and B) Representative images (A) and quantification (B) of plate colony counting of blood, heart, lung, kidney, liver, spleen, duodenum, colon tissues and fecal samples, harvested from mice after single oral gavage. (C-J) Routine blood examination of WBC, Neu, Lym, Mon, PLT, HCT, MCV and MCH with long-term administration. WBC, white blood cell; Neu, neutrophils; Lym, lymphocytes; Mon, monocytes; PLT, platelet; HCT, hematocrit; MCV, mean corpuscular volume and MCH, mean

corpuscular. (K) Representative H&E-stained sections of duodenum, colon and other organs (heart, lung, kidney, liver and spleen) post 4 weeks treatments, scale bar = 50 μm. (L-O) Serum levels of AST, ALT, CRP and UREA in wild type mice within 4 weeks bacterial exposure. AST, aspartate aminotransferase; ALT, alanine aminotransferase; CRP, C-reaction protein; UREA, urea. Normal values: AST, 54-298 IU/L; ALT, 17-77 IU/L; CRP, < 0.5mg/dL; UREA, 7.75-15.52 mmol/L. The dashed lines represent the normal ranges. (P and Q) Representative images and quantification of β-catenin (P) and Cyclin D1 (Q) expression in duodenum with 4 weeks bacterial exposure, scale bar = 50 μm. Data are presented as mean ± SEM. Statistical significance determined using an unpaired t test. ns, $P > 0.05$.

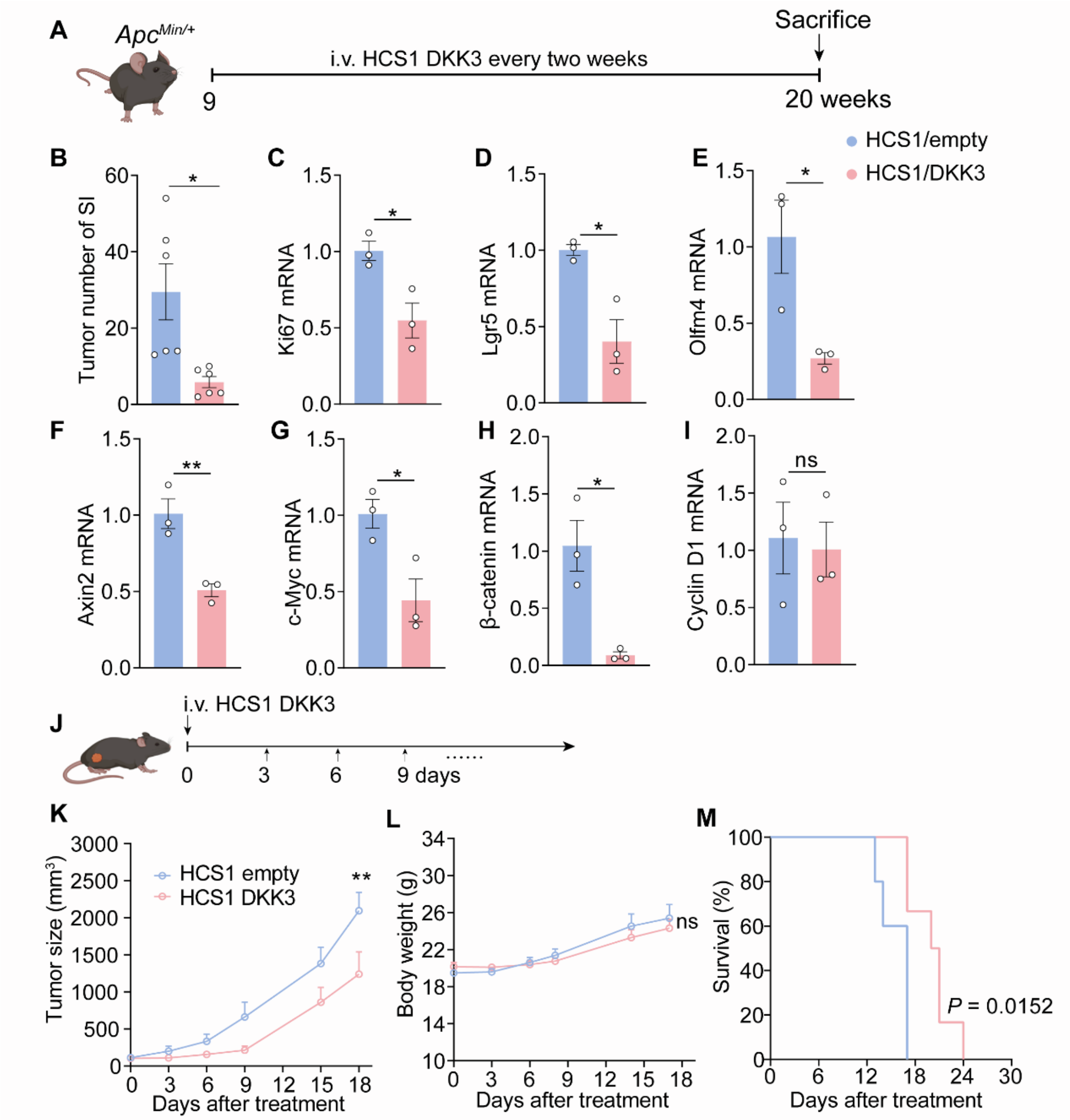

Figure S10. Antitumor effects of HCS1/DKK3 in *Apc*$^{Min/+}$ mice and MC38 models. Related to Figures 4 and 5. (A) Experimental scheme of engineered HCS1 in *Apc*$^{Min/+}$ mice. (B) Tumor numbers in small intestine at the 20th week, n = 6. (C to E) mRNA level of *Ki67* (C), *Lgr5* (D) and *Olfm4* (E) in tumors with HCS1/DKK3-treated. (F to I) mRNA level analysis of *Axin2* (F), *c-Myc* (G), *β-catenin* (H) and *Cyclin D1* (I) in *Apc*$^{Min/+}$ mice tumors with HCS1/DKK3-treated. (J) Experimental scheme of engineered HCS1 in MC38 subcutaneous tumor model. (K to M) Tumor growth (K), body weight change (L) and animal survival (M) of MC38-bearting mice after bacterial injection (n = 5-6). Data are presented as mean ± SEM. Statistical significance determined using an unpaired t test or two-way ANOVA. ns, $P > 0.05$, $*P \leq 0.05$ and $** P \leq 0.01$.

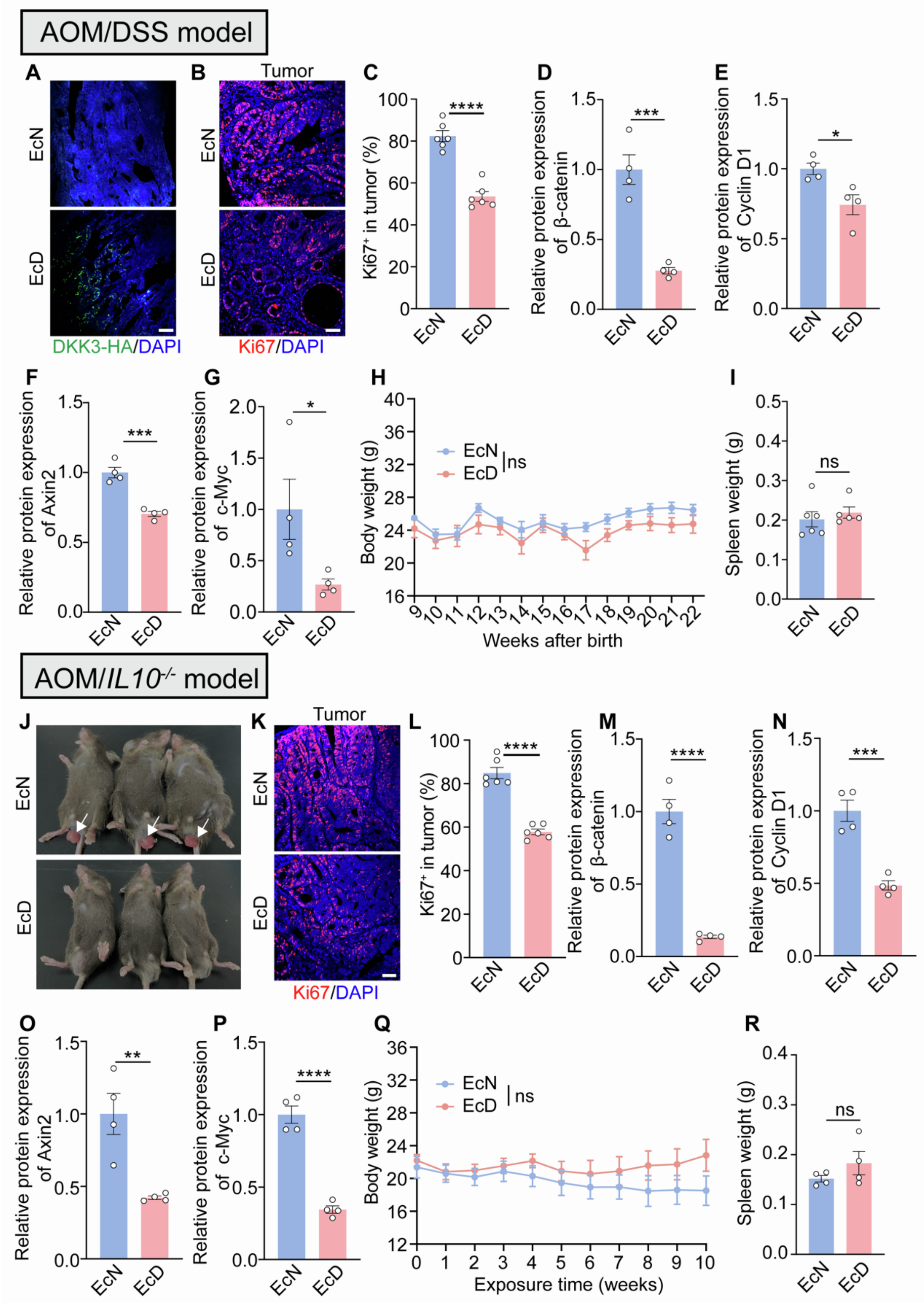

Figure S11. Effects of ISCs proliferation and Wnt signaling with EcD administration in AOM/DSS and AOM/*IL10*$^{-/-}$ models. Related to Figure 6. (A) IF staining image of HA-tagged DKK3 in colon tumor. (B and C) IF staining image of Ki67 in colon tumors and its signal quantification. (D to G) Quantitative analysis for western blot analysis of β-catenin (D), Cyclin D1 (E), Axin2 (F) and c-Myc (G) in AOM/DSS-treated tumors. (H and I) Body weight (H) and spleen weight (I) changes

of AOM/DSS mice with EcD treated. (J) The physical appearance of AOM/*IL10*$^{-/-}$ mice with EcN and EcD exposure. (K and L) IF staining image of Ki67 in colon tumors and its signal quantification. (M to P) Quantitative analysis for western blot analysis of β-catenin (M), Cyclin D1 (N), Axin2 (O) and c-Myc (P) in AOM/*IL10*$^{-/-}$ mice tumors. (Q and R) Body weight (Q) and spleen weight (R) changes of AOM/*IL10*$^{-/-}$ mice with EcD treated. Data are presented as mean ± SEM. Statistical significance determined using an unpaired t test. ns, $P > 0.05$, *$P \leq 0.05$, ** $P \leq 0.01$, *** $P \leq 0.001$ and **** $P \leq 0.0001$.

Table S1. The basic information of the patients and healthy cohort. Related to STAR Methods.

Cohorts	No	Sex	Age	Stage	Sample	Application
CRC patients	1	Male	63	Rectal cancer	Tissue	IF, PDO
	2	Male	72	Colon cancer	Tissue	IF
	3	Male	65	Rectal cancer	Tissue	IF
	4	Male	62	Rectal cancer	Tissue	PDO
	5	Male	76	Colon cancer	Tissue	PDO
	6	Female	63	Colon cancer	Tissue	IF
	7	Female	70	Colon adenoma	Tissue	IF
	8	Female	62	Colon cancer	Tissue	PDX
	9	Male	66	Colon cancer	Stool	Diagnosis
	10	Male	68	Colon cancer	Stool	Diagnosis
	11	Male	58	Rectal cancer	Stool	Diagnosis
	12	Male	62	Colon cancer	Stool	Diagnosis
	13	Male	73	Rectal cancer	Stool	Diagnosis
	14	Female	37	Rectal cancer	Stool	Diagnosis
	15	Male	65	Rectal cancer	Stool	Diagnosis
	16	Male	68	Colon cancer	Stool	Diagnosis
	17	Male	62	Rectal cancer	Stool	Diagnosis
	18	Female	78	Rectal cancer	Stool	Diagnosis
	19	Female	61	Rectal cancer	Stool	Diagnosis
	20	Female	62	Colon cancer	Stool	Diagnosis
IBD patients	1	Male	39	Crohn's Disease	Stool	Diagnosis
	2	Male	55	Ulcerative Colitis	Stool	Diagnosis
	3	Male	28	Crohn's Disease	Stool	Diagnosis
	4	Male	36	Crohn's Disease	Stool	Diagnosis
	5	Male	37	Ulcerative Colitis	Stool	Diagnosis
	6	Male	42	Crohn's Disease	Stool	Diagnosis
	7	Female	40	Crohn's Disease	Stool	Diagnosis
	8	Male	30	Ulcerative Colitis	Stool	Diagnosis
Healthy control	1	Male	37	NA	Stool	Diagnosis
	2	Male	43	NA	Stool	Diagnosis
	3	Female	27	NA	Stool	Diagnosis
	4	Male	40	NA	Stool	Diagnosis
	5	Female	31	NA	Stool	Diagnosis
	6	Male	60	NA	Stool	Diagnosis
	7	Male	36	NA	Stool	Diagnosis
	8	Female	42	NA	Stool	Diagnosis
	9	Male	58	NA	Stool	Diagnosis
	10	Male	25	NA	Stool	Diagnosis
	11	Male	65	NA	Stool	Diagnosis
	12	Female	35	NA	Stool	Diagnosis

NA, not applicable.

Table S2. Summary of the strains used in this study. Related to STAR Methods.

Lable	Bacterial strain	Plasmids	Use(s)
EcO-sfGFP	EcN	pJ23119-speFL-sfGFP	Fluorescence detection and imaging
EcO-Rluc8	EcN	pJ23119-speFL-Rluc8	Bioluminescence imaging
EcN	EcN	pJ23119-empty (backbone)	Drug protein expression control
EcD	EcN	pJ23119-pelB-DKK3-HA	Drug protein expression
HCS1/empty	HCS1	pJ23119-empty (backbone)	Drug protein expression control
HCS1/DKK3	HCS1	pJ23119-pelB-DKK3-HA	Drug protein expression
