# Supplementary material for: Synthetically engineered probiotic suppresses colorectal cancer via inhibition of Wnt/β-catenin signaling
Source: Cell Rep Med. 2026 Aug 18;7(8):102913. doi: 10.1016/j.xcrm.2026.102913 (PMC13522751; doi:10.1016/j.xcrm.2026.102913)
Supplement: Supplementary file 2 [file mmc2.pdf]

Cell Reports
Medicine

Article

Synthetically engineered probiotic suppresses colorectal cancer via inhibition of Wnt/β-catenin signaling

Graphical abstract

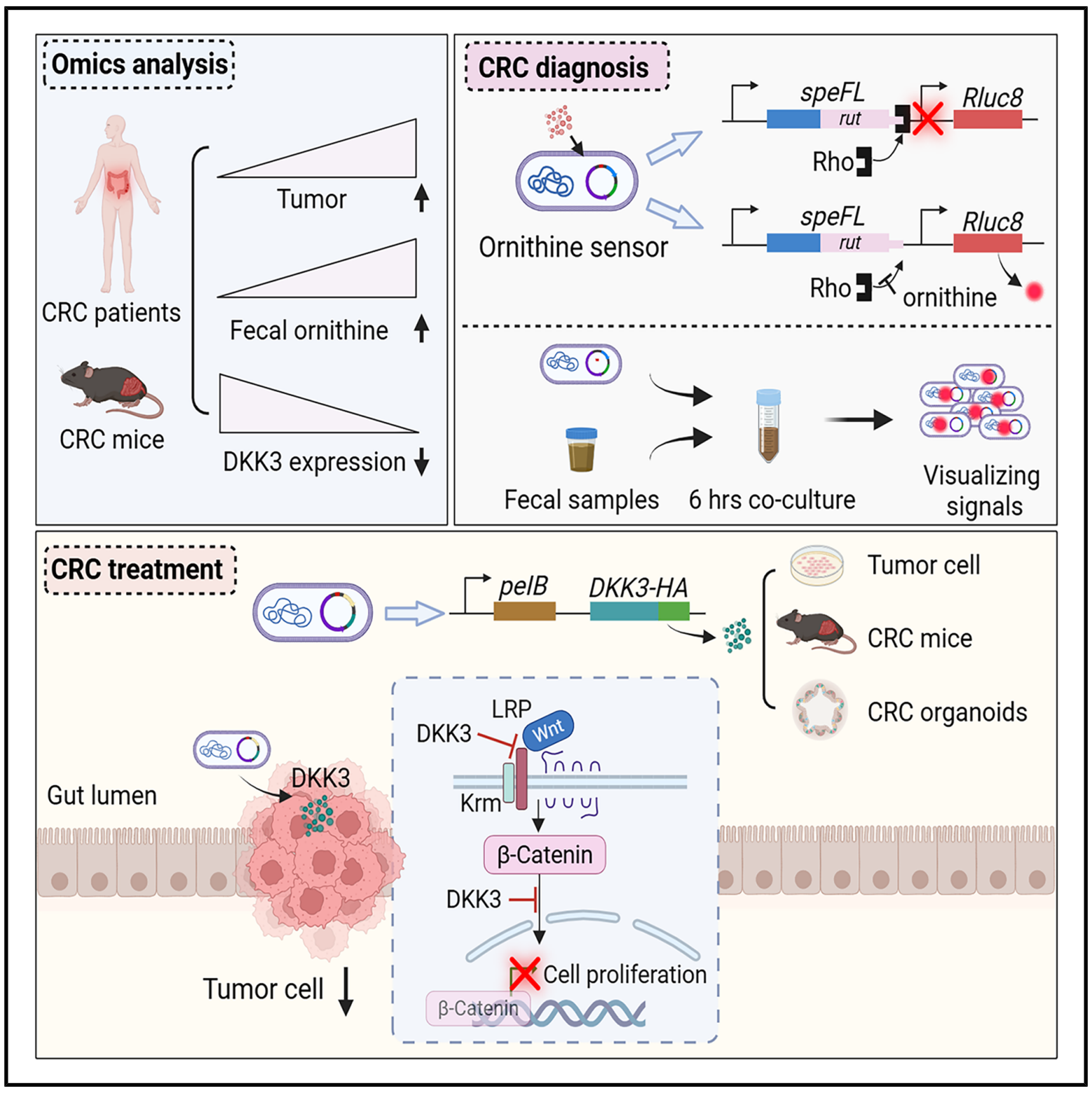

Highlights

- EcO-sensing ornithine enables early screen of intestinal adenoma non-invasively
- EcD inhibits cell proliferation in multiple tumor models via Wnt signaling
- EcD retards the patient-derived organoid and PDX growth offers translation potential

Authors

Yujie Sun (孙妤婕), Yu Zhao (赵宇), Kang Han (韩康), ..., Shengen Yi, Jun Zhou (周俊), Jin Hai Zheng (郑金海)

Correspondence

junzhou82@hnu.edu.cn (J.Z.),
jhzheng@hnu.edu.cn (J.H.Z.)

In brief

Sun et al. construct an engineered probiotic theranostic platform for early detection and precise therapy of CRC. EcO enables non-invasive screening of adenoma through bacteria-fecal co-incubation, while EcD could retard tumor development via inhibiting Wnt activation in mice, patient-derived organoids, and PDX tumor models.

Sun et al., 2026, Cell Reports Medicine 7, 102913
August 18, 2026 © 2026 The Author(s). Published by Elsevier Inc.
https://doi.org/10.1016/j.xcrm.2026.102913

CellPress

Cell Reports Medicine

CellPress OPEN ACCESS

Article

Synthetically engineered probiotic suppresses colorectal cancer via inhibition of Wnt/β-catenin signaling

Yujie Sun (孙妤婕),[1,2,5] Yu Zhao (赵宇),[1,2,5] Kang Han (韩康),[1,2,4,5] Wulong Hu,[1,2] Guofan Peng,[1,2] Shengen Yi,[3] Jun Zhou (周俊),[1,2,*] and Jin Hai Zheng (郑金海)[1,2,6,*]

[1]School of Biomedical Sciences, Hunan University, Changsha 410082, China
[2]Hunan Key Laboratory of Animal Models and Molecular Medicine, Hunan University, Changsha 410082, Hunan, China
[3]Department of General Surgery, the Second Xiangya Hospital of Central South University, Changsha 410011, Hunan, China
[4]Xiangtan Central Hospital, the Affiliated Hospital of Hunan University, Xiangtan 411100, Hunan, China
[5]These authors contributed equally
[6]Lead contact
*Correspondence: junzhou82@hnu.edu.cn (J.Z.), jhzheng@hnu.edu.cn (J.H.Z.)
https://doi.org/10.1016/j.xcrm.2026.102913

SUMMARY

Colorectal cancer (CRC) is one of the most prevalent malignancies globally. Recent breakthrough of synthetic biology stimulates the utilization of engineered bacteria for cancer diagnosis and treatment. We perform a comprehensive fecal metabolomic analysis in CRC mouse models and patients and identify ornithine as a specific metabolic biomarker for tumor development. We subsequently engineer an *E. coli* Nissle 1917 (EcN)-based bioluminescent reporter via ornithine-responsive tumor detection. For therapeutic intervention, we construct a probiotic system that locally releases Dickkopf 3 (DKK3), a potent Wnt signaling antagonist to retard tumor growth. Oral administration of the bacteria demonstrates reduced tumor burden and improved survival outcomes in multiple preclinical models, and the anticancer efficacy is further confirmed in CRC patient-derived organoids and patient-derived xenograft (PDX) model. Taken together, we develop a novel probiotic platform combing non-invasive diagnostic capability with targeted therapeutic delivery for CRC management, underscoring the strong potential for clinical translation and eventual application in preventive oncology.

INTRODUCTION

Colorectal cancer (CRC) remains one of the most lethal malignancies globally. Moreover, the incidence of CRC among the young population is rising,[1] implying that timely detection and therapeutic intervention is imperative. Current screening modalities in clinical application include colonoscopy, fecal occult blood tests, and multi-target stool DNA analysis, but certain limitations remain.[2] For example, although colonoscopy is the gold standard, its invasiveness, high cost, and risks of bleeding hinder widespread adoption for CRC screening.[3] Hence, there is an unmet need to develop non-invasive alternatives for early diagnosis of CRC.

With recent advances in synthetic biology, the inherent capacity of bacteria to synthesize bioactive molecules *in situ* enables their development as precision tools for cancer diagnosis and therapy.[4] An editable probiotic *E. coli* Nissle 1917 (EcN) system with programmed gene circuits detects liver metastases in mice via urine test.[5] The EcN has also been engineered to produce salicylate and release GM-CSF, PD-L1^{Nb}, and CTLA-4^{Nb} for CRC detection and immunotherapy.[6] Another study developed a *S. typhimurium*-based quorum sensing system initiating green fluorescent protein (GFP) specifically in high-density colonies within tumors.[7] Although these systems represent significant advances, they are limited by requirements of additional exogenous inducers or dependence on advanced disease status. To construct a smart-responsive system for CRC detection, we engineered a probiotic biosensor that is capable of detecting the level of specific metabolic biomarker in the feces of CRC mouse models and patients and subsequently emitting bioluminescence signal through a simple co-incubation of bacteria and feces.

Engineered probiotic EcN has emerged as an optimized antitumor vaccination platform for enhanced production and cytosolic delivery of neoepitope-containing peptide arrays, achieving specific T cell-mediated anticancer immunity.[8] The synergetic combination of IFN-γ-producing bacteria and PD-1 blockade was demonstrated as a therapeutic strategy for overcoming immunotherapy resistance in metastatic tumors.[9] These approaches preferred to utilize bacteria to strengthen antitumor immune responses in the tumor microenvironment (TME) with depletion of tumor nutrients, or production of tumor-inhibitory mediators. Although bacterial-mediated TME modulation has been suggested for potential cancer treatments, challenges

© 2026 The Author(s). Published by Elsevier Inc.
This is an open access article under the CC BY license (http://creativecommons.org/licenses/by/4.0/).

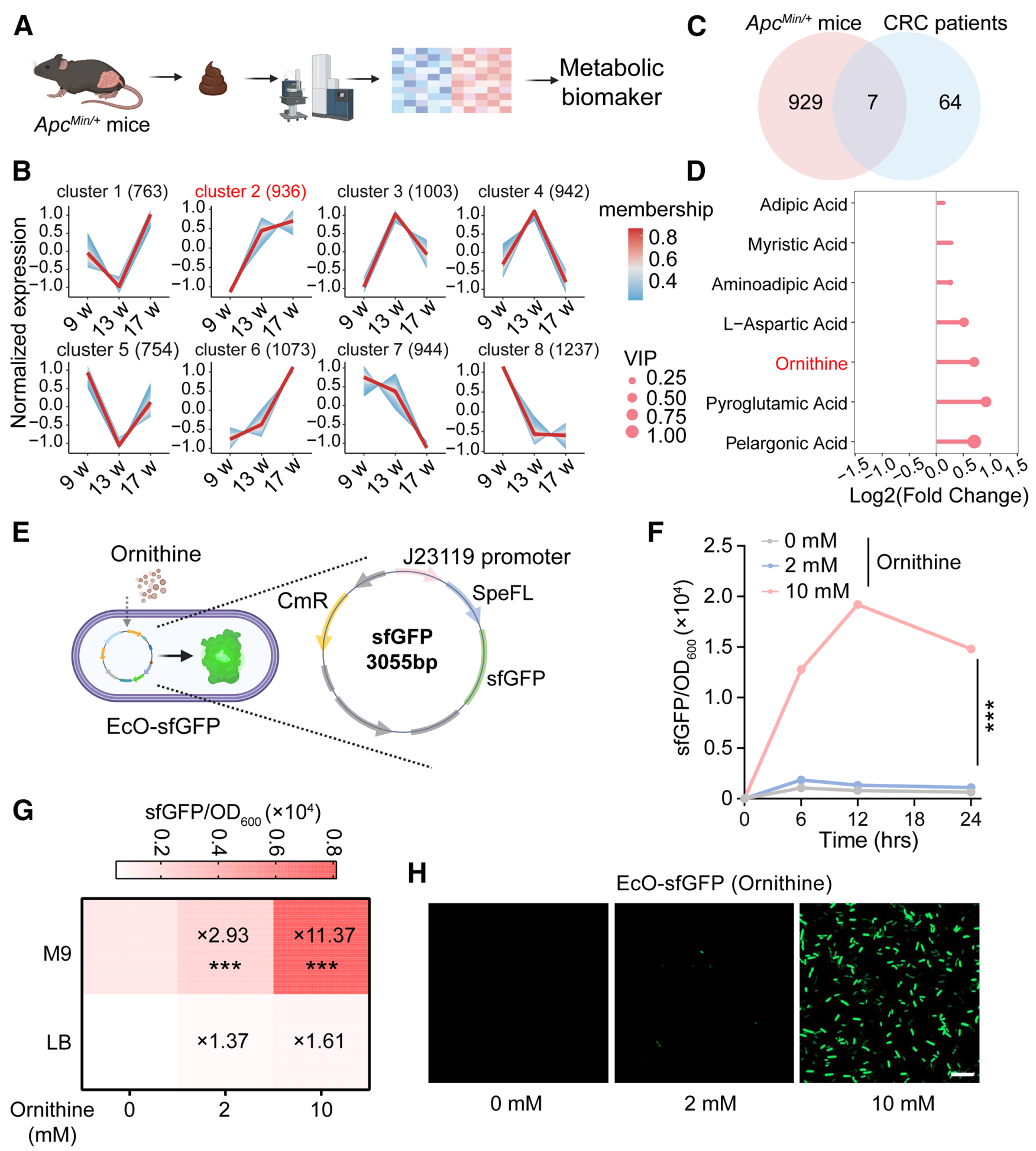

Figure 1. Ornithine up-regulated in CRC and construction of ornithine-responsive genetic circuit

(A) Schematic diagram of analysis of the tumor metabolite biomarker.

(B) Eight metabolite clusters were identified with temporal patterns.

(C) Venn diagram for fecal metabolites from cluster 2 and differential fecal metabolites between CRC patients and healthy samples.

(D) Lollipop chart showing the common metabolites expressed between tumor mice and CRC patients, with the *x* axis indicating fold change and the *y* axis representing differential metabolites. The dot size reflects VIP value.

(E) Schematic diagram of ornithine-responsive plasmid. Ornithine induces engineered bacteria to produce the green fluorescent protein.

(F) Fluorescence intensity change with ornithine-responsive plasmid treated with 0, 2, 10 mM ornithine in M9 medium within 24 h.

(G) Fluorescence intensity measurement of engineered EcO-sfGFP in LB medium and M9 medium with different ornithine concentrations.

(H) Visualization of EcO-sfGFP in the absence (0 mM) and presence of ornithine (2, 10 mM) by confocal fluorescence microscopy; scale bar, 10 μm.

Data are presented as mean ± SEM. Statistical significance determined using an unpaired *t* test; $^{***}p \leq 0.00$.

remain in limited systemic biosafety and therapeutic efficiency,[10] such as immune evasion and tumor relapse.[11]

The canonical Wnt/β-catenin signaling pathway, mediated through T cell factor/lymphoid enhancer factor (TCF/LEF), plays pivotal roles in embryonic development and tissue homeostasis.[12] Importantly, dysregulation of this pathway has been well established as a fundamental driver of colonic tumorigenesis,[13–15] indicating it is one of the most promising molecular targets for CRC therapy. For example, Wnt2 could serve as a target to augment the antitumor immunity in CRC.[16] Besides, the inhibitory proteins of Wnt-dependent signaling such as Dickkopfs (DKKs) have been reported as Wnt signaling antagonists. DKK members (DKK1–DKK4) function through direct binding to lipoprotein receptor-related protein 5/6 (LRP5/6) or Kremen protein 1 (Krm1), preventing their interaction with Frizzled receptors and downstream β-catenin stabilization, eventually inhibiting the transcriptional activation of Wnt target genes.[17–19]

In this study, we developed a synthetic biology-driven theranostic platform enabling non-invasive early diagnosis and targeted therapeutic intervention of CRC. The dual-function system demonstrated non-invasive fecal-based screening of intestinal tumors through engineered probiotic sensing, and the localized delivery of DKK3 via oral administration of EcD potently suppresses tumor progression in mouse models and CRC patient-derived organoids. The work establishes a novel paradigm in CRC treatment by integrating synthetic biology with precision medicine, offering a clinically translatable solution that bridges the gap between early diagnosis and effective therapy.

RESULTS

Identification of ornithine as a metabolic biomarker for CRC

Cancer progression has been associated with alterations in the microbiome and in the metabolites of feces, plasma, serum, and tissues, suggesting their potential as novel diagnostic biomarkers for various cancers, including CRC.[20] Herein, we collected the stools of *Apc*$^{Min/+}$ mice at different stages across tumor development and then performed untargeted liquid chromatography-mass spectrometry (LC-MS) to identify the early metabolic signatures of CRC in fecal samples (Figure 1A). Clustering analysis revealed eight distinct metabolic clusters (Figure S1). To search potential metabolic biomarkers in early stages of colorectal tumorigenesis, we focused on cluster 2 containing 936 metabolites, which showed a progressive increase during tumorigenesis, especially from the 9th to 13th week, which is a critical window for the early process of CRC development (Figure 1B). With metabolic profiling on fecal samples from CRC patients and healthy cohorts, 71 metabolites up-regulated

in CRC patients were identified.[21] Subsequently, the overlay analysis revealed 7 common metabolites elevated in both murine and human CRC (Figure 1C). After comprehensive consideration of their variation magnitude, solubility properties, and compatibility with prokaryotic sensing systems, ornithine was selected for further validation (Figure 1D). In addition, ornithine has been reported to be significantly up-regulated in adenoma patients.[22] To develop an ornithine-responsive biosensor, we engineered EcN with the speF operon to obtain EcO-sfGFP,[23] which generates fluorescence upon ornithine induction (Figure 1E). The induction efficiency was tested by measuring the fluorescence signal after exposure to ornithine; stable and robust GFP signal was detected within 6 h of ornithine exposure (Figure 1F). We also performed a dose-dependent assay in LB or M9 medium to optimize detection sensitivity and found that 10 mM ornithine in M9 minimal medium elicited the strongest fluorescence signal (Figures 1G and S2A), in which a bright visible signal was observed (Figure 1H). Moreover, the presence of ornithine did not impair bacterial growth as checked by OD_{600} measurement (Figures S2B and S2C). Importantly, since ornithine is a basic amino acid and plays a role in urea cycle, we supplied different concentrations of arginine, lysine, and histidine to the culture of EcO-sfGFP, and the result showed that none of these could trigger the expression of sfGFP (Figure S2D). In addition, similar results were observed in tyrosine, tryptophan, and adipic acid supplementation (Figure S2E). These data indicate that the engineered bacteria could sense ornithine levels specifically *in vitro* and have potential application for CRC screening.

Engineered probiotic biosensor for non-invasive early detection of CRC

Given the heterogeneous composition of murine fecal matrices, there are potential signal interferences from dietary components or gut microbes. Bioluminescent reporters are widely applied in preclinical studies due to their high sensitivity and the absence of endogenous background signal.[24] To optimize the bacterial sensor in cancer diagnosis with high efficiency, sensitivity, and accuracy, sfGFP was replaced with a bioluminescence reporter Rluc8, to avoid endogenous signal interference (Figure 2A). The bacterial biosensor system was validated by exogenous ornithine supplementation (Figures 2B, 2C, S3A, and S3B).

To evaluate the CRC diagnostic potential of our engineered biosensor system, fresh stools of age-matched wild-type (WT) and $Apc^{Min/+}$ mice of C57BL/6J background were collected and homogenized in M9 medium; the supernatants were then diluted in M9 medium and cultured with the bacterial biosensors EcO-Rluc8 (Figure 2D). Using the IVIS (In Vivo Imaging System), the signals were measured at 3, 6, and 24 h; the output signals indicated differential luciferase activities at 6 and 24 h after co-culturing bacteria with stool samples from healthy and tumor-bearing mice. To this end, 6 h was chosen for further study to improve diagnosis efficiency (Figure 2E). Then, mice at different ages were randomly selected, and their stools were co-cultured with EcO-Rluc8; the results showed that the bioluminescence intensities of stools of tumor-bearing mice were significantly higher than those of healthy mice ($p = 0.0001$; Figure 2F). Moreover, age-stratified analysis was performed by continuously monitoring the ornithine level within a same batch of mice across different stages of tumor development. Significantly higher output signals could be detected with stools of $Apc^{Min/+}$ mice in the initial stage of tumorigenesis ($p < 0.01$; Figure 2G), which indicated that an early therapeutic intervention could be beneficial starting from the 11th week.

To further validate the stability of the bacterial biosensor system, we kept the stools overnight at 4°C or room temperature, and the visible signals in tumor feces could still be detected without notable attenuation (Figures S3C–S3E). However, upon heat treatment of the stool supernatants and co-incubation with bacteria, there was no difference in the fecal signals between healthy and tumor-bearing mice (Figure S3F); we speculated that this is because high temperature partially degraded the ornithine. These results suggest that the ornithine-responsive system is stable and sensitive for early diagnosis of tumor progression.

Given that ornithine was identified as a promising metabolic biomarker, we further determined whether elevated ornithine levels are specifically linked to tumorigenesis. To assess the diagnostic specificity, the biosensor was tested in well-established gastrointestinal disease models, including dextran sulfate sodium (DSS)-induced and $IL10^{-/-}$ murine models of inflammatory bowel disease (IBD), as well as in aged mice; there was no visual difference in bioluminescence signals in disease conditions compared with healthy controls (Figures S4A–S4C), which strongly indicated that the elevation of ornithine was specifically associated with tumor development rather than being a general feature of gastrointestinal pathologies. More importantly, we collected stools from CRC patients and healthy controls in order to access the clinical application potential of the ornithine sensor (Figure 2H). Under the optimized condition, analysis of samples from 12 randomly selected CRC patients and healthy controls revealed that the biosensor signal was significantly increased in CRC patients compared with healthy individuals ($p < 0.01$; Figures 2I and 2J). Interestingly, stool samples of IBD and healthy individuals have also been checked (Figure S4D), and the results showed that there was no significant difference between the two groups (Figure S4E). The above findings indicated that changes in ornithine levels in stools were associated with tumor development rather than general intestinal disorders. Taken together, these findings suggest that the biosensor system could not only be used for tumor detection but also manifested clinical application potential for early diagnosis and monitoring of CRC progression.

Engineered probiotics release DKK3 that inhibits proliferation of cancer cells

The Wnt/β-catenin signaling pathway controls various cellular processes, such as proliferation, differentiation, and motility.[12] To develop a bacterial-mediated Wnt inhibition strategy, we focused on the secretory protein of the DKK family, which contains four members (DKK1, DKK2, DKK3, and DKK4) that have emerged as modulators of Wnt/β-catenin signaling.[25] To investigate the potential link between DKKs and CRC, we comprehensive profiled DKK1–4 expression in human CRC datasets of the Gene Expression Profiling Interactive Analysis (GEPIA) database and found that DKK3 was significantly down-regulated in both colorectal READ and COAD cancer tissues compared with

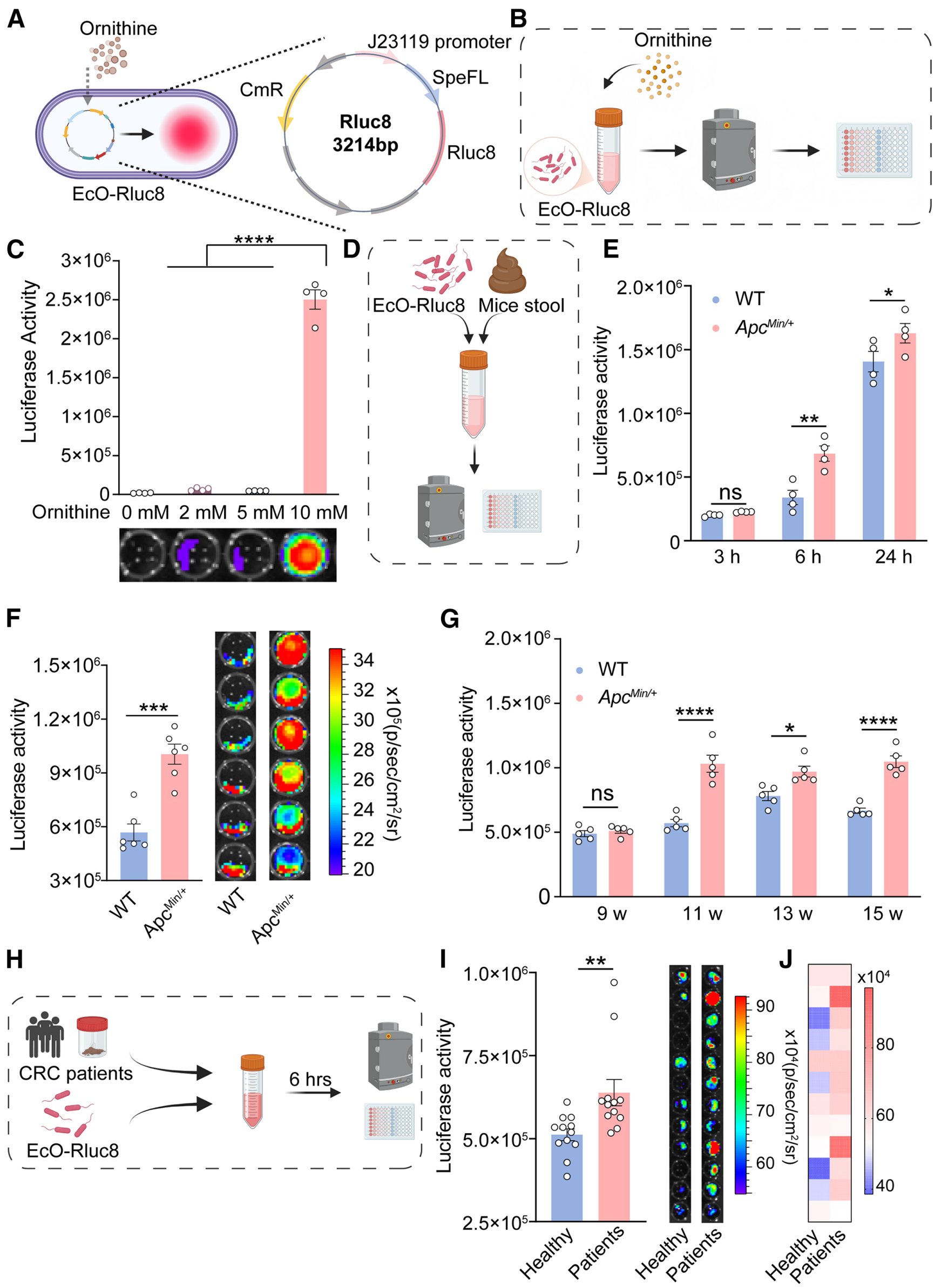

Figure 2. Ornithine-responsive reporter Rluc8 for CRC diagnosis

(A) Schematic diagram of ornithine-responsive plasmid. Ornithine induces engineered bacteria to produce the Rluc8 protein, which reacted with coelenterazine to emit bioluminescence signals.

(B) Schematic diagram of EcO-Rluc8 sensing ornithine *in vitro*.

(C) Luciferase activity of engineered bacteria with different concentrations of ornithine examined by IVIS *in vitro*.

(D) Schematic diagram of ornithine-responsive EcO-Rluc8 to detect mice.

(E) Bioluminescence signal outputs at different co-culture times.

(F) Bioluminescence signals after co-incubating bacteria and stools of wild-type mice or $Apc^{Min/+}$ mice checked by IVIS.

(G) Continuous monitoring of bioluminescence signals with bacteria and stool co-culture during disease progression.

(H) Schematic diagram of the efficiency of EcO-Rluc8 responsive to stools from CRC patients and healthy individuals.

(I) Quantitative bioluminescence signals of co-culture of EcO-Rluc8 with stools from CRC patients.

(J) Heatmap of the bioluminescence signals.

Data are presented as mean ± SEM. Statistical significance determined using an unpaired *t* test; $^{*}p \leq 0.05$, $^{**}p \leq 0.01$, $^{***}p \leq 0.001$, and $^{****}p \leq 0.0001$.

normal colorectal tissues ($p < 0.05$; Figure 3A). In contrast, DKK1 was only down-regulated in COAD tumors (Figure S5A) and DKK2 and DKK4 exhibited no significant changes between tumor and paired normal tissues (Figures S5B and S5C). Moreover, the low expression of DKK3 is associated with poor overall survival of CRC patients (Figures 3B and 3C) and single-cell RNA sequencing analysis validated the higher DKK3 expression in normal tissues (Figure S5D). Furthermore, we also assessed DKK3 expression via immunofluorescence staining with 5 paired CRC patient samples, and the intensity of DKK3 in tumor tissues was obviously down-regulated compared with adjacent normal tissues (Figures 3D and 3E). Consequently, we engineered probiotic EcN to express and secrete functional DKK3, termed as EcD, and the expression and secretion of DKK3 was successfully confirmed *in vitro* (Figures 3F, 3G, and S4E). Besides, in a preliminary experiment, we tested the plasmid loss of EcN harboring the pJ23119 backbone and its derived pJ23119-DKK3 without antibiotic pressure *in vitro* for 3 and 7 days; the results indicate that the EcD could maintain the plasmid well (Figures S5F and S5G). Besides, when EcD was cultured with VNP20009/lux, an attenuated bacteria derived from *Salmonella typhimurium* 14028s, in non-antibiotic condition for 7 days, there was no VNP20009/lux containing pJ23119-DKK3 in the obtained bacterial culture (Figure S5H), which suggested the stability of the plasmid of EcD without potential of horizontal gene transfer.

To evaluate the therapeutic potential of bacteria derived DKK3, the supernatant of bacterial culture was concentrated and incubated with human colon cancer cells (HCT116 and HT29). The colony formation counts and CCK8 assay indicated that the supernatant with DKK3 did inhibit the proliferation of HCT116 cells and HT29 cells (Figures 3H–3J and S5I–S5K). In addition, the quantitative reverse-transcription PCR (RT-qPCR) assay revealed that DKK3-treated HCT116 cells significantly decreased the levels of *c-Myc*, *β-catenin*, and *Axin2* (Figure 3K, $p < 0.05$), which are downstream targets of Wnt signaling; a similar phenomenon was shown in the HT29 cell line (Figure S5L). These results suggest that DKK3 suppresses the transcription of Wnt genes in CRC cells.

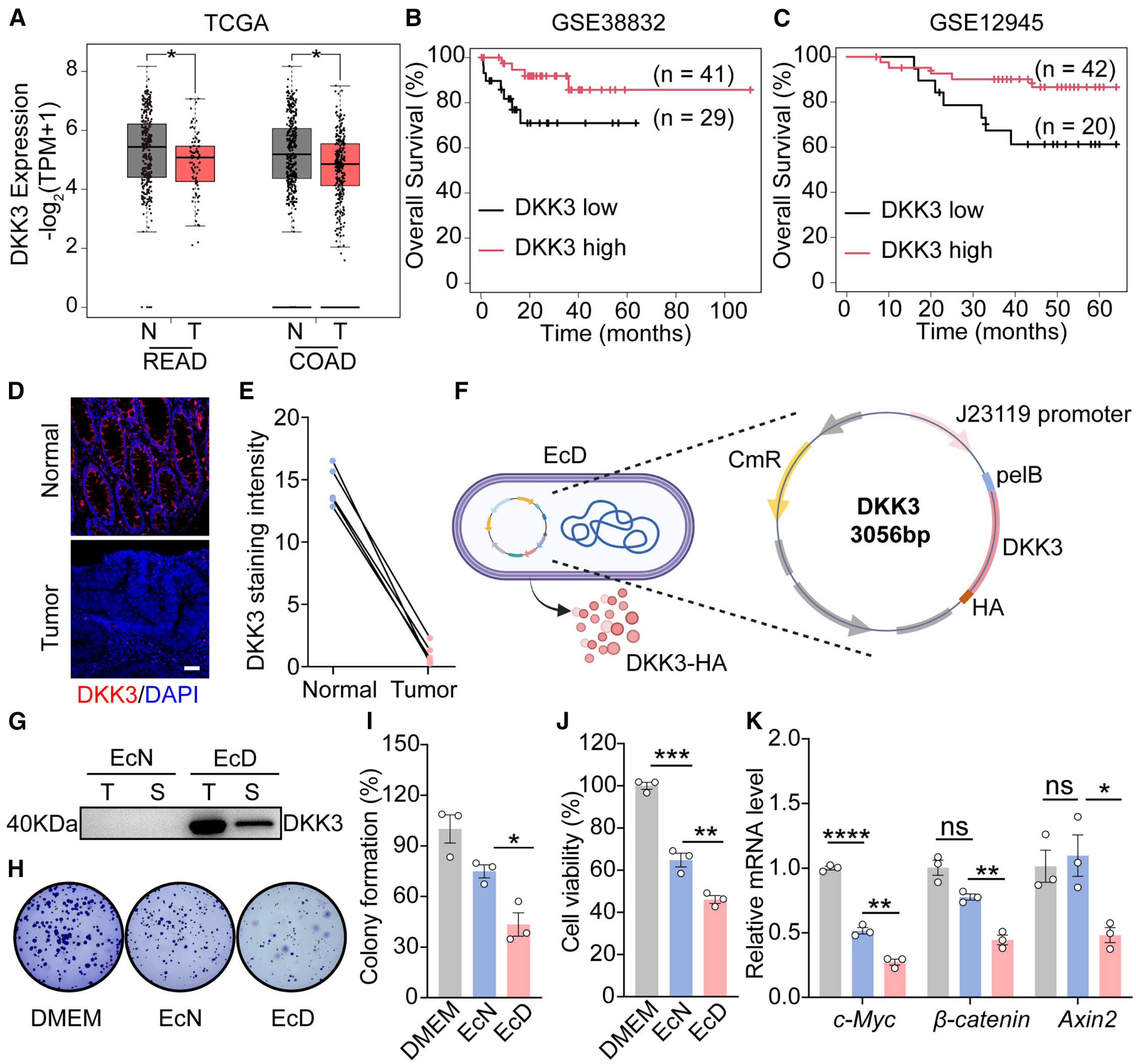

Figure 3. Therapeutic bacterial design and activity evaluation *in vitro*

(A) Expression of DKK3 in colorectal cancer in the GEPIA database. READ (num(N) = 318, num(T) = 92), COAD (num(N) = 349, num(T) = 275). N, normal, T, tumor.

(B and C) Kaplan-Meier plot of overall survival using the GSE38832 (B, log rank $p = 0.0402$, hazard ratio [HR] = 0.29, 95% confidence interval [CI], [0.09–1.02]) and GSE12945 datasets (C, log rank $p = 0.0307$, HR = 0.30, 95%CI, [0.1–0.95]).

(D) Representative images of immunofluorescence staining for DKK3 expression in normal and tumor tissues from CRC patients; scale bar, 50 μm.

(E) Quantification of DKK3 expression intensity in normal and tumor tissues, $n = 5$.

(F) Schematic diagram of engineered DKK3-secreting EcN (EcD).

(G) Expression and secretion of DKK3 within EcD were detected by western blot. T, bacterial culture total; S, culture supernatant.

(H) Representative images with crystal violet staining of HCT116 cells after DKK3 co-incubation for 6 days.

(I) Colony formation ratio after DKK3 co-incubation for 6 days.

(J) Cell viability of HCT116 treated with DKK3 for 6 days.

(K) Quantification of mRNA levels of HCT116 treated with DKK3 for 6 days and checked by RT-qPCR.

Data are presented as mean ± SEM. Statistical significance determined using an unpaired t test; $^{*}p \leq 0.05$, $^{**}p \leq 0.01$, $^{***}p \leq 0.001$, and $^{****}p \leq 0.0001$.

Engineered probiotics release DKK3 to suppress intestinal tumor growth

Genetic mutation of adenomatous polyposis coli (Apc) has been detected in 70% of patients with sporadic CRC, indicating a crucial role of Wnt activation in tumorigenesis. Thus, the $Apc^{Min/+}$ transgenic mouse was created as a spontaneous cancer model for CRC research that maximum mimics clinical pathology.[26,27] To optimize the conditions of administration, we evaluated the intestinal colonization kinetics of EcN in WT mice following a single oral dose of 1×10^8, 1×10^9, 1×10^{10} colony-forming unit (CFU); considering both the persistence of colonization and the physiological burden on the animals, a dose of 1×10^9 CFU was chosen for subsequent experiments (Figure S6A). We also confirmed the plasmid stability *in vivo* (Figure S6B). Next, to evaluate whether the DKK3 derived from the engineered probiotic could inhibit intestinal tumor growth, $Apc^{Min/+}$ mice were orally administered

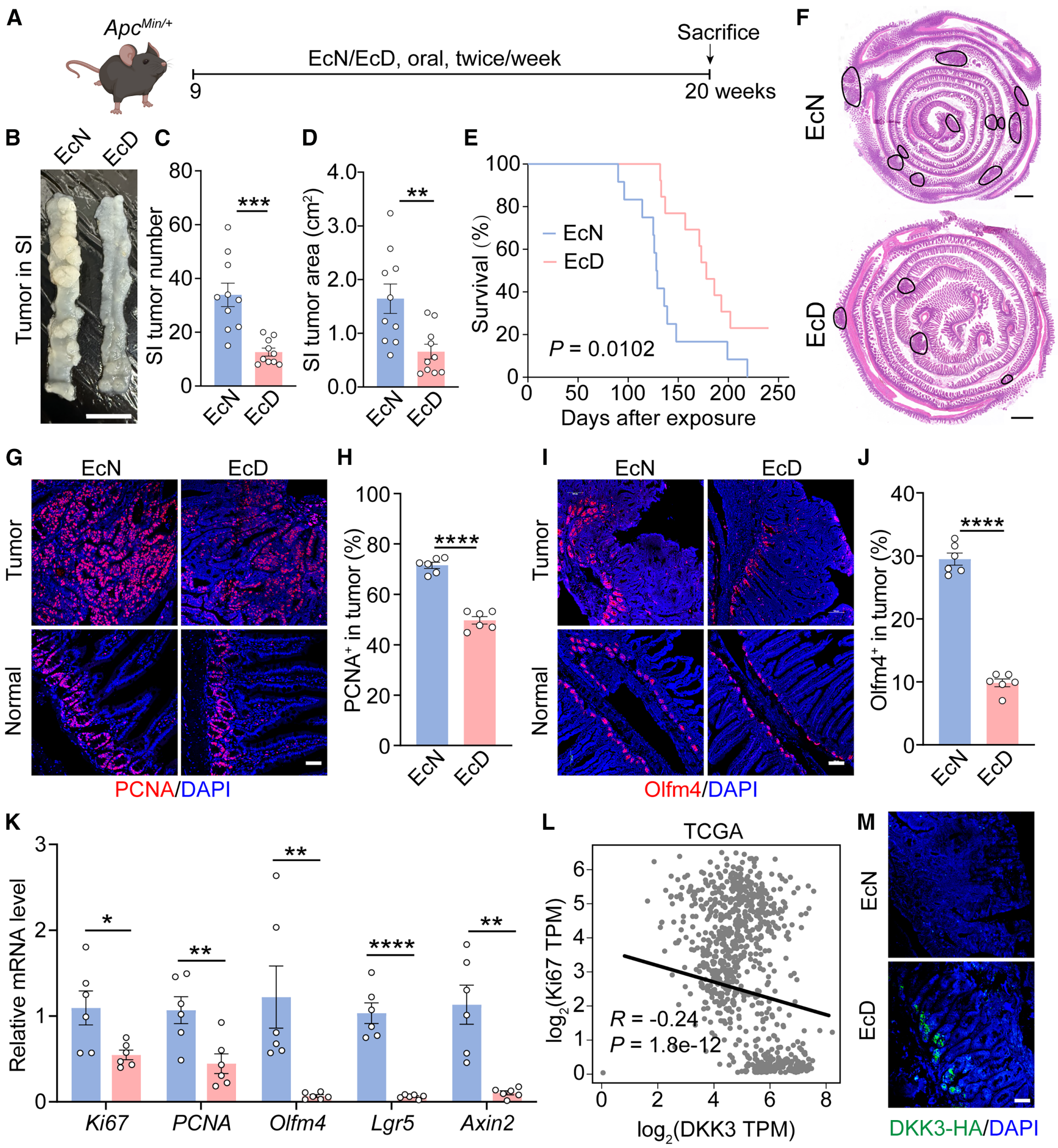

Figure 4. Therapeutic effects of engineered probiotic in *Apc*$^{Min/+}$ mice

(A) Experimental design. The 9-week-old male *Apc*$^{Min/+}$ mice were divided into two groups and were orally administered EcN or EcD twice a week until the 20th week; then the tumor numbers were counted and tumor tissues were collected.

(B) At the 20th week, mice were sacrificed, and intestines were excised and graphed; scale bar, 2 cm.

(C and D) Analysis of tumor numbers and total tumor area in small intestines, $n = 10$.

(E) Mice survival curve of EcN- ($n = 12$) and EcD- ($n = 13$) treated groups.

(F) Representative H&E-stained histology images of EcN- and EcD-treated mice; scale bars, 1 mm.

(G–J) Immunofluorescence staining and signal intensity analysis with PCNA (G and H) and Olfm4 (I and J) in tumors; scale bars, 50 μm.

(K) mRNA analysis of ISC proliferation markers (*Ki67*, *PCNA*, *Olfm4*, *Lgr5*, and *Axin2*) in tumors treated with EcN and EcD.

(L) TCGA analysis of expression relationship between DKK3 and Ki67 in CRC patients.

A tendency line was added based on the "*R*." (M) Immunofluorescent staining of HA-tagged DKK3 secreted from EcD in adenoma; scale bar, 50 μm.

Data are presented as mean ± SEM. Statistical significance determined using an unpaired *t* test; *$p \leq 0.05$, **$p \leq 0.01$, ***$p \leq 0.001$, and ****$p \leq 0.0001$.

EcN or EcD twice a week since the ninth week and then sacrificed at the 20th week (Figure 4A). We observed an overall reduction in tumor number in the small intestine of *Apc*$^{Min/+}$ mice post EcD treatment ($p \leq 0.01$; Figures 4B and 4C). In addition, EcD treatment also induces a marked decrease in total tumor areas compared with EcN ($p \leq 0.01$; Figure 4D), and there were no

significant changes of body and spleen weights in mice with long-term EcD exposure (Figures S6C and S6D). Importantly, with EcD gavage, the survival of tumor-bearing mice was prolonged significantly ($p \leq 0.05$; Figure 4E). Moreover, histological analysis with H&E staining further confirmed the reduction of tumor nodules after treatment with EcD, while abundant tumor lesions spread throughout the whole small intestine in the EcN-treated mice (Figure 4F). To further assess the distribution of bacteria in *Apc$^{Min/+}$* mice, bacterial counts in mice at early and late stages were performed (Figure S6E), and PCR and western blot of EcD validated the gene existence and expression of DKK3 in tumor tissues (Figures S6F and S6G). In addition, functional DKK3 concentration checked by ELISA and immunofluorescence staining revealed the obviously increased expression of DKK3 in tumor post-EcD administration (Figures S6H–S6J).

Wnt signaling is indispensable to maintain stemness and the undifferentiated state of intestinal stem cells (ISCs).[28,29] During tissue development and homeostasis, canonical Wnt signaling is known to be the primary pathway for regulating ISC proliferation and self-renewal.[30] To assess the impact of EcD treatment on tumor cell proliferation and expansion, immunofluorescence staining was performed to analyze the expression of PCNA, Olfm4, and Ki67; we found that all of these proliferative indexes were drastically decreased in tumor tissues post-EcD treatment, but did not change in the non-tumor areas (Figures 4G–4J and S6K–S6O). In addition, the mRNA levels of *Ki67*, *Lgr5*, and *Olfm4* in tumors also declined significantly with EcD exposure (Figure 4K), which is consistent with the association between DKK3 and *Ki67*, *Lgr5*, and *Olfm4* levels determined by Pearson correlation analysis in The Cancer Genome Atlas (TCGA) database (Figure 4L, S6P, and S6Q). To further investigate bacterial localization and exogenous DKK3 expression *in situ*, intestinal tissues were stained with hemagglutinin (HA) tag, and positive signals were observed in adenoma post-EcD treatment, suggesting targeted release of DKK3-HA via oral administration of the engineered probiotic (Figure 4M); furthermore, immunofluorescent co-staining of PCNA and HA tag revealed that tumor shrinkage was associated with high concentrations of DKK3 (Figure S6R). These results indicated that oral administration of EcD could inhibit tumor development via inhibition of tumor cell proliferation.

Given that Lgr5$^+$ intestinal cells play an indispensable role in radiation-induced regeneration, we further evaluated the role of EcD using *Lgr5$^{GFP\text{-}CreERT2}$* mice. Besides, Cre-inducible *Rosa26$^{LSL\text{-}tdTomato}$* mice were employed for lineage tracing (*Lgr5$^{GFP\text{-}CreERT2}$; tdTomato*). Tamoxifen (TAM) induces Cre activity in Lgr5$^+$ cells in a mosaic fashion, driving heritable tdTomato expression post-EcD treatment.[31,32] After 3 days of TAM administration, the lengths of tdTomato-marked epithelial strips along the crypt-villus axes were reduced in EcD-treated mice, indicating the robust inhibition of ISC activities (Figures S7A–S7C). In summary, these results indicated that administration of EcD could repress the proliferation of Lgr5$^+$ cells and thus suppress tumor progression.

EcD reduces tumor progression by inhibiting Wnt/β-catenin signaling

To investigate how DKK3 secreted from the probiotic interacts with Wnt signaling components to interrupt its activity, we next performed the pull-down assay and confirmed that DKK3 secreted from EcD physically interacts with Krm1 and β-transducin repeat-containing protein (βTrcp) (Figures 5A and S8A). Krm1 has been identified as a negative regulator of the canonical Wnt signaling pathway and synergistically works with the secreted Wnt inhibitor DKK by competing for the LRP5/6 co-receptor.[19,33] Previous findings also demonstrated that DKK3 mediated Wnt signaling by interacting with βTrcp, which mediated the β-catenin degradation.[34,35] To comprehensively understand the mechanisms underlying the tumor-suppressive activities of EcD, we performed RNA sequencing analysis on tumor tissues from *Apc$^{Min/+}$* mice with EcN or EcD treatment (Figure 5B). Heatmap revealed that the genes associated with cell proliferation were down-regulated with EcD exposure (Figure 5C); in addition, gene set enrichment analysis and Kyoto Encyclopedia of Genes and Genomespathway analysis demonstrated that the drastically down-regulated genes were enriched in the Wnt signaling pathways (Figures 5D and 5E). Furthermore, we observed reduced expression of the classical Wnt/β-catenin signaling targets via immunofluorescence staining, such as β-catenin and CyclinD1, which are usually used as reporters of Wnt/β-catenin signaling activation (Figures 5F–5I, S8B, and S8C). The mRNA levels of genes related to Wnt/β-catenin signaling, such as *Wnt3a*, *LRP5*, *β-catenin*, *c-Myc*, and *Cyclin D1* were decreased in EcD-treated tumors (Figures 5J–5O). Moreover, the antitumor effect of EcD has also been validated in female *Apc$^{Min/+}$* mice (Figures S8D–S8H). To evaluate the potential of EcD for treating established colorectal neoplasia, we postponed the treatment and began from the age of 12 weeks (Figure S8I), when the adenomas have already well established in the small intestine; similar tumor shrinkages were observed in both male (Figures S8J and S8K) and female mice (Figures S8L and S8M), suggesting that the DKK3 derived from the engineered EcD could also repress tumor even at a later stage.

To investigate the safety profiles of this therapeutic approach, we subsequently conducted a long-term toxicological assessment with multiple administrations of the engineered probiotics in healthy mice. As the plate colony counts of blood, heart, lung, kidney, liver, spleen, duodenum, colon, and feces showed, there was no translocation of bacteria in other tissues other than the gut (Figures S9A and S9B). Consistent with prior research that repeated gavage of EcN did not induce a proinflammatory response,[36] there was no significant difference in the total white blood cells, neutrophils, lymphocytes, and monocytes among each group (Figures S9C–S9F), and the parameters such as PLT, HCT, MCV and MCH also showed no significant changes compared with untreated healthy mice (Figures S9G–S9J), indicating there was no notable immune stimulation and disruption for hematopoiesis. Furthermore, histopathological analysis of gut tissues and organs revealed no significant morphological changes post-long-term exposure of EcD (Figure S9K). Additionally, serum levels of aspartate aminotransferase, alanine aminotransferase, C-reaction protein, and urea, key indicators of liver and kidney function, showed no significant differences among the 3 groups (Figures S9L–S9O). More importantly, immunofluorescence staining with β-catenin and Cyclin D1 in the duodenum showed that there was no off-target effect of DKK3 in the normal intestines (Figures S9P and S9Q). These results demonstrated

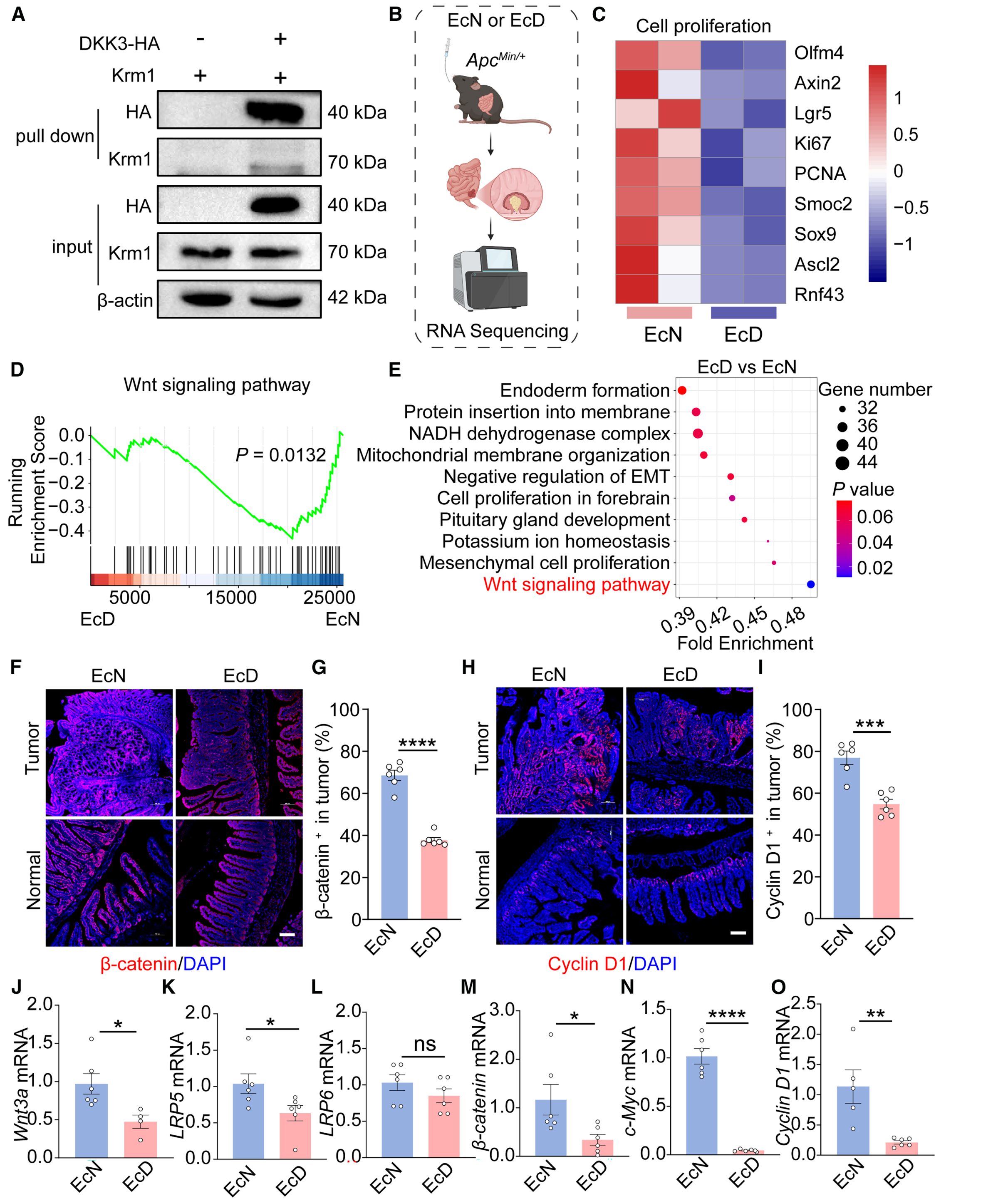

Figure 5. EcD inhibits Wnt/β-catenin signaling in *Apc*$^{Min/+}$ mice

(A) The interaction of DKK3-HA secreted from EcD with the Krm1 protein was validated by pull-down assay in HCT116 cells.
(B) Scheme of gene expression analysis of Wnt/β-catenin signaling by RNA sequencing.
(C) Heatmap showing the mRNA levels of ISC markers in tumors with EcN and EcD treatment.
(D) Gene set enrichment analysis (GSEA) of Wnt/β-catenin signaling in tumors post-EcD treatment at the 20th week.
(E) Gene Ontology analysis revealing the down-regulated expressed genes post-EcD treatment.
(F–I) Immunofluorescence staining and signal intensity analysis with β-catenin (F and G) and Cyclin D1 (H and I) in tumor tissues and normal areas; scale bars, 50 μm.
(J–O) The mRNA analysis of Wnt/β-catenin signaling markers in EcN- and EcD-treated tumors checked by RT-qPCR.
Data are presented as mean ± SEM. Statistical significance determined using an unpaired *t* test; *$p \leq 0.05$, **$p \leq 0.01$, ***$p \leq 0.001$, and ****$p \leq 0.0001$.

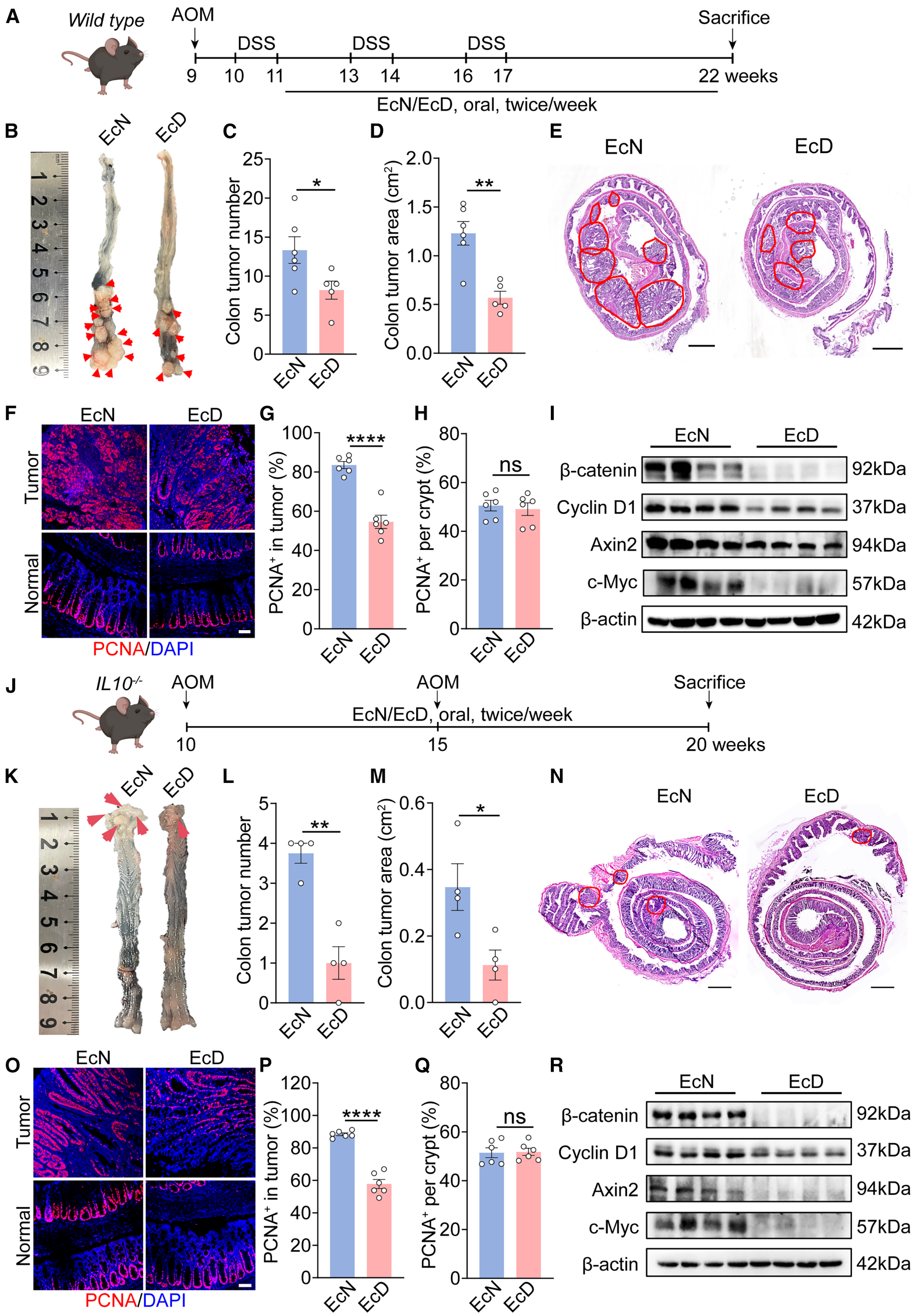

Figure 6. Therapeutic effects in carcinogen-induced mouse colorectal cancer models

(A) Experimental schematic of AOM/DSS-induced colorectal cancer mice. Mice were orally administrated EcN or EcD twice a week since the 11th week.

(B) At the end of treatment, mice were sacrificed, and the representative pictures of the opened colon are shown.

(legend continued on next page)

that EcD exhibits a favorable long-term safety profile in mice, laying the groundwork for clinical translation.

To explore a better delivery platform with less-frequent administration and expand the therapeutic potential of DKK3 beyond oral probiotics, we engineered an attenuated *Salmonella typhimurium* strain HCS1 for systemic delivery. HCS1 is an excellent oncolytic bacterial strain with specific tumor targeting and good safety profiles as reported previously.[37] In $Apc^{Min/+}$ mice, intravenously administered with HCS1/DKK3 every 2 weeks, tumor burden was obviously reduced in the small intestines (Figures S10A and S10B) and cell proliferation markers *Ki67*, *Lgr5*, and *Olfm4* were decreased with HCS1/DKK3 exposure (Figures S10C–S10E), with reduction of the mRNA levels of genes related to Wnt/β-catenin signaling (Figures S10F–S10I). Next, we evaluated the bacterial therapeutic efficacy in the subcutaneous MC38 tumor model; intravenous administration of HCS1/DKK3 could also inhibit tumor development and prolong animal survival without weight loss or notable toxicity (Figures S10J–S10M). These findings demonstrated that the bacteria-secreted DKK3 as a therapeutic platform could work efficiently in various bacterial secretion systems in different tumor models with good universality and operability.

EcD reduces tumor progression in carcinogen-induced CRC models

Azoxymethane (AOM) is a carcinogen used to mimic the development of sporadic CRC. Co-administration of AOM and DSS could develop a colitis-related mouse colon carcinogenesis model.[38] To assess the anticancer activity of engineered probiotic, AOM/DSS-induced mice were orally administrated with EcN or EcD twice a week since the end of the first DSS cycle (Figure 6A). The tumor numbers and total tumor areas in colon were significantly reduced post-EcD therapy ($p < 0.05$; Figures 6B–6E). HA signals were also observed in colon tumors with oral delivery of EcD (Figure S11A). Likewise, the expressions of PCNA and Ki67 were decreased in tumor tissues with EcD treatment (Figures 6F–6H, S11B, and S11C). Western blot and RT-qPCR analysis revealed the down-regulation of Wnt signaling in tumor tissues as well (Figure 6I and S11D–S11G), which further confirmed that the engineered probiotic represses tumor growth by inhibiting tumor cell proliferation. There was no significant change of body and spleen weights in mice with EcD exposure, suggesting the good biosafety of EcD (Figures S11H and S11I).

The AOM/interleukin-10 knockout (AOM/$IL10^{-/-}$) mouse model provides critical insights into inflammation-driven tumorigenesis, which closely mimics clinical IBD-associated colon cancer.[39] Mice orally administered EcN showed more severe disease phenotypes compared with the EcD-treated group, such as rectal prolapse and tumor burden (Figures 6J–6N and S11J). Immunofluorescence staining also demonstrated a substantial decrease in the level of PCNA and Ki67 in tumor areas, and the protein expression of Wnt signaling targets declined with EcD exposure (Figures 6O–6R and S11K–S11P). Besides, there was no significant change of body and spleen weights (Figures S11Q and S11R). These results revealed that EcD could ameliorate inflammation-induced tumor in various murine models and process good biosafety.

Probiotic-derived DKK3 inhibits the Wnt activity and the growth of CRC patient-derived organoids and PDX model

To further assess the clinical antitumor potential of DKK3 in CRC patients, we established patient-derived organoids from CRC patients (Figure 7A). Bacterial culture supernatant of EcD was co-incubated with organoids and revealed that DKK3 supplementation markedly repressed the growth of CRC patient-derived organoids (Figures 7B–7D). Next, immunofluorescence staining with HA tag antibody showed positive signaling specifically located in the EcD-treated group, confirming the DKK3-HA uptake in CRC organoids (Figure 7E). The PCNA staining indicated that cell proliferation of organoid was significantly inhibited by EcD treatment ($p < 0.001$; Figures 7F and 7G). In addition, analysis of Wnt signaling by RT-qPCR further showed that DKK3-HA supplementation decreased the level of Wnt signaling targets, inhibiting the Wnt signaling pathway in CRC organoids (Figure 7H). Moreover, to better simulate patient conditions and assess the clinical potential of EcD, we developed a CRC patient-derived xenograft (PDX) model in NCG mice. Tumor-bearing mice were administered HCS1/DKK3 intravenously, and tumor volume was recorded subsequently (Figure 7I). The results showed that tumor growth was significantly retarded by the injection of HCS1/DKK3 ($p < 0.05$; Figures 7J and 7K), suggesting the antitumor activity and biosafety of HCS1/DKK3. These findings highlight the therapeutic potential of the engineered bacteria in treating clinical patients.

DISCUSSION

As adenoma progress slowly to carcinoma in the gastrointestinal tract clinically, there is a critical need for improved strategies for both early detection and therapeutic intervention. Here, we constructed EcO and EcD as an engineered probiotic theranostic platform for early cancer detection and precise therapy. First, EcO enables non-invasive adenoma diagnosis through

(C and D) Analysis of tumor numbers (C) and total tumor areas (D) in colon with EcN ($n = 6$) and EcD ($n = 5$) treatment.
(E) Representative H&E-stained histological images of colon; scale bar, 2 mm.
(F–H) Immunofluorescence staining (F) and signal intensity analysis with PCNA in tumor tissues (G) and normal areas (H); scale bar, 50 μm.
(I) Western blot analysis of β-catenin, Cyclin D1, Axin2, and c-Myc in tumor tissues of AOM/DSS-treated mice.
(J) Experimental schematic of AOM/$IL10^{-/-}$ colorectal cancer mice. Mice were orally administrated EcN or EcD twice a week.
(K) At the 20th week, mice were sacrificed, and the representative pictures of the dissected colon are shown.
(L and M) Analysis of tumor numbers and total tumor areas in colon, $n = 4$.
(N) Representative mice images in EcN- and EcD-treated mice; scale bar, 2 mm.
(O–Q) Immunofluorescence staining (O) and signal intensity analysis with PCNA in tumor tissues (P) and normal areas (Q); scale bar, 50 μm.
(R) Western blot analysis of β-catenin, Cyclin D1, Axin2, and c-Myc in tumor tissues of AOM/$IL10^{-/-}$ models.
Data are presented as mean ± SEM. Statistical significance determined using an unpaired *t* test; $*p \leq 0.05$, $**p \leq 0.01$, and $****p \leq 0.0001$.

CellPress
OPEN ACCESS

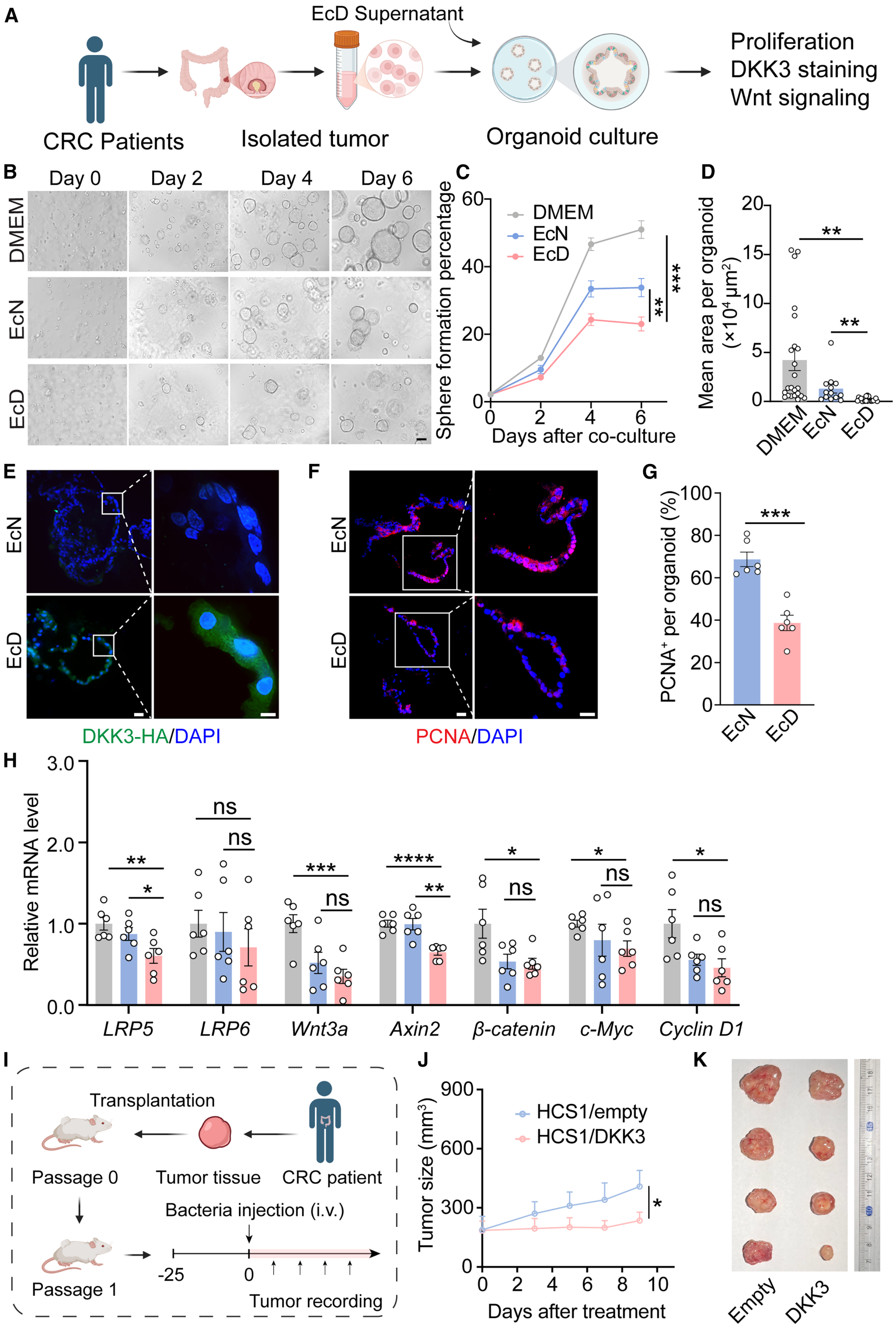

Figure 7. Therapeutic validation of engineered probiotic in CRC organoids and PDX model

(A) Experimental design of CRC organoids and DKK3 co-incubation.

(B) Representative bright-field images of tumor organoids derived from CRC patients under different treatments; scale bar, 100 μm.

(legend continued on next page)

bacteria-fecal co-incubation. Then, EcD could retard tumor development by inhibiting Wnt activation in multiple mouse models and CRC patient-derived organoids.

Ornithine serves as a metabolic biomarker for engineered probiotic diagnosis of tumor progression: although colonoscopy is effective at reducing CRC incidence and mortality, its limitations, including invasiveness, high cost, and procedural risks, emphasize the need for alternative diagnostic approaches. With the advances in synthetic biology, bacteria could be manipulated to respond to tumor environment[40,41] or external stimulus.[42–44] Bacteria are well adapted to sense and respond to the local environmental changes, and this capacity can be utilized for cancer diagnosis. Previous work showed that oral delivery of EcN to release salicylate could increase salicylate level in the urine of adenoma-bearing mice.[6] This approach needs bacteria to enter into the intestine and tends to require severe disease progression. Early diagnosis tools to improve the intervention efficacy are still rare. Herein, our strategy focused on developing a probiotic based non-invasive method to measure changes of certain metabolites in the feces during tumor progression. Using overlay analysis of fecal metabolomics of CRC patients and $Apc^{Min/+}$ mice, we identified ornithine as one of the conserved up-regulated metabolites associated with intestinal tumor progression rather than with other intestinal diseases such as IBD or aging.[22,45–47] We, therefore, established an effective cancer diagnosis platform with probiotic sensing ornithine. Unlike the pre-existing findings, our strategy proposed a non-invasive detection concept that simply involved incubating the bacteria with fecal supernatant rather than bacteria entering into the body. Furthermore, the co-culture of the ornithine-responsive sensor EcO-Rluc8 with fecal samples from mice and human individuals with multiple intestinal diseases verified its specificity for identifying CRC individuals, which suggests this approach could be potentially applied in the clinic for early screening of CRC. In brief, the engineered probiotic EcO could achieve early screening via sensing ornithine variation of CRC stools in a safe and convenient pattern.

Engineered probiotic releases DKK3 for CRC treatment: Wnt/β-catenin signaling is implicated in many physiological processes, including development, tissue regeneration, and cancer.[48] CRC is driven by the dysregulation of multiple key signaling pathways, whose cumulative and coordinated effects enable the acquisition of hallmarks essential for tumorigenesis,[49] Wnt/β-catenin signaling pathway is an evolutionarily conserved and unique signaling pathway that regulates gene expression, cell invasion, migration, proliferation, and differentiation in the initiation and progression of CRC. As a result, Wnt signaling inhibition represents a promising therapeutic strategy for cancer treatment.[50] Numerous studies have explored that many FDA-approved drugs and natural products have the potential to function as Wnt inhibitors, such as chlorquinaldol[51] and quercetin.[52] While these agents demonstrate Wnt inhibitory activities, they encounter obvious limitations, such as poor targeting, toxicity to normal tissues, and incomplete pharmacokinetic profiles. Thus, these highlight the urgent need to develop next-generation Wnt inhibitors with increased efficacy and specificity for cancer therapy. Previous studies have identified DKK proteins as antagonists of Wnt/β-catenin signaling, and the mRNA level of *DKK3* was down-regulated in CRC tissues.[53–55] To this end, we established a probiotic expressing DKK3 to inhibit tumor growth, and we found tumor development in multiple orthotopic tumor models could be repressed via oral administration of the modified probiotic. Our findings also revealed that probiotic-secreted DKK3 effectively inhibits the growth of CRC organoids and PDX tumors, highlighting its strong clinical potential. Unlike bacterial therapies that rely on delivering cytokines or immune checkpoint inhibitors to the TMEs,[42,56,57] EcD directly suppresses intestinal tumor progression by inhibiting cancer cell proliferation, offering an effective alternative for immune-insensitive "cold tumors." Beyond efficiency, EcD exhibited no systemic toxicity, even post-long-term administration, supporting its safety profiles for CRC therapy.

In conclusion, this study established a probiotic platform that facilitates early detection and precise therapy of CRC. This strategy consists of two steps: first, to develop the sense-and-response functional bacteria as a non-invasive approach for tumor detection, and then, targeted delivery of Wnt antagonist to retard tumor development by repressing tumor cell proliferation. With its dual diagnostic and therapeutic potentials, this probiotic platform offers a promising therapeutic strategy for CRC.

Limitations of the study

Although our engineered probiotic platform demonstrates forceful potential for CRC theranostics, some key aspects remain to be further explored. For example, while the delivery of DKK3 exhibits significant tumor-suppressive efficacy, other DKK protein family members have not been investigated. Although our experimental design aimed to clarify the ability of EcD to directly induce tumor cell apoptosis and inhibit its proliferation by inhibiting the classic Wnt/β-catenin signaling pathway, the immunomodulatory function of DKK3 still deserved to be further explored. Despite these limitations, our study offers a promising alternative for convenient, non-invasive CRC diagnosis and therapy.

(C) The time course of organoid size under different treatments.
(D) The size of tumor organoids with DMEM, EcN, or EcD treatment.
(E) Immunofluorescence staining of DKK3-HA in CRC organoids; scale bars, 100 μm.
(F and G) Representative images (F) and quantitative analysis (G) of immunofluorescence staining of PCNA in CRC organoids; scale bars, 50 μm.
(H) mRNA analysis of Wnt signaling markers in organoids treated with EcN and EcD by RT-qPCR.
(I) Schematic of the experimental procedure and schedule for evaluating the antitumor activity in PDX model.
(J) Tumor growth curves of mice with the indicated treatments (n = 4).
(K) Images of isolated tumors at 9 days post-treatments.
Data are presented as mean ± SEM. Statistical significance determined using an unpaired t test; $*p \leq 0.05$, $**p \leq 0.01$, $***p \leq 0.001$, and $****p \leq 0.0001$.

RESOURCE AVAILABILITY

Lead contact

Further information and requests for resources and reagents should be directed to and will be fulfilled by the lead contact, Jin Hai Zheng (jhzheng@hnu.edu.cn).

Materials availability

This study did not generate new unique reagents.

Data and code availability

- Metabolomics of fecal samples have been deposited at MetaboLights with accession number MTBLS12971. RNA sequencing data of tumor have been deposited at Gene Expression Omnibus (GEO) with accession number GEO: GSE307572.
- This paper analyzes existing, publicly available data, accessible at https://doi.org/10.1186/s40168-021-01208-5, GEO: GSE38832, GSE12945, and GSE201349.
- This paper does not report original code.
- Any additional information required to reanalyze the data reported in this paper is available from the lead contact upon request.

ACKNOWLEDGMENTS

This work was supported by the National Natural Science Foundation of China (82273831, 32270890, 82303771, and 82311540161), Hunan Natural Science Foundation of China (2023JJ20022 and 2023JJ0007), and Hunan Provincial Key Laboratory of Anti-Resistance Microbial Drugs, the Third Hospital of Changsha (2023TP1013). We would like to thank Shu Zhu (USTC, China), Yinhua Jin (Zhejiang University), Jianfeng Wu (Xiamen University), and GemPharmatech Co., Ltd for providing mouse strains. We thank Yansong Xiong, Yalan Hao, and the Analytical Instrumentation Center of Hunan University for the assistance in confocal microscopy and animal facility.

AUTHOR CONTRIBUTIONS

Y.S., Y.Z., and K.H. performed the experiments and analyzed the data. Y.S. and Y.Z. drafted the figures and wrote the manuscript. S.Y., G.P., and W.H. made contributions to the experimental work and patient sample collection. J.H.Z. and J.Z. conceived the study, analyzed the data, and wrote and revised the manuscript.

DECLARATION OF INTERESTS

The authors declare no competing interests.

STAR★METHODS

Detailed methods are provided in the online version of this paper and include the following:

- KEY RESOURCES TABLE
- EXPERIMENTAL MODEL AND STUDY PARTICIPANT DETAILS
  - Patients and samples
  - Mice
  - Note on sex of animals
  - Bacterial strains and culture condition
  - Tumor cell lines
- METHOD DETAILS
  - Metabolomics and analysis
  - Plasmids construction
  - Growth curve and determination of fluorescence intensity
  - Bioluminescence imaging
  - Cell experiment
  - Tumor models and treatment
  - Pull-down assay
  - Western blot
  - Quantification of DKK3
  - Plasmid stability assessment
  - RNA-sequencing and analysis
  - Colonization of EcD in mice
  - Immunofluorescent (IF) staining
  - Histopathology
  - Hematological assessments
  - RNA isolation and RT-qPCR
  - Organoid culture and treatment
  - Colorectal cancer patient-derived xenograft (PDX) model
- QUANTIFICATION AND STATISTICAL ANALYSIS
  - Statistical analysis

SUPPLEMENTAL INFORMATION

Supplemental information can be found online at https://doi.org/10.1016/j.xcrm.2026.102913.

Received: September 15, 2025
Revised: April 3, 2026
Accepted: June 15, 2026
Published: August 18, 2026

REFERENCES

1. Zhang, H., Tian, Y., Xu, C., Chen, M., Xiang, Z., Gu, L., Xue, H., and Xu, Q. (2025). Crosstalk between gut microbiotas and fatty acid metabolism in colorectal cancer. Cell Death Discov. *11*, 78. https://doi.org/10.1038/s41420-025-02364-5.
2. Ladabaum, U., Dominitz, J.A., Kahi, C., and Schoen, R.E. (2020). Strategies for colorectal cancer screening. Gastroenterology *158*, 418–432. https://doi.org/10.1053/j.gastro.2019.06.043.
3. Yin, H., Xie, J., Xing, S., Lu, X., Yu, Y., Ren, Y., Tao, J., He, G., Zhang, L., Yuan, X., et al. (2024). Machine learning-based analysis identifies and validates serum exosomal proteomic signatures for the diagnosis of colorectal cancer. Cell Rep. Med. *5*, 101689. https://doi.org/10.1016/j.xcrm.2024.101689.
4. Pedrolli, D.B., Ribeiro, N.V., Squizato, P.N., de Jesus, V.N., and Cozzetto, D.A.; Team AQA Unesp at iGEM 2017 (2019). Engineering microbial living therapeutics: the synthetic biology toolbox. Trends Biotechnol. *37*, 100–115. https://doi.org/10.1016/j.tibtech.2018.09.005.
5. Danino, T., Prindle, A., Kwong, G.A., Skalak, M., Li, H., Allen, K., Hasty, J., and Bhatia, S.N. (2015). Programmable probiotics for detection of cancer in urine. Sci. Transl. Med. *7*, 289ra84. https://doi.org/10.1126/scitranslmed.aaa3519.
6. Gurbatri, C.R., Radford, G.A., Vrbanac, L., Im, J., Thomas, E.M., Coker, C., Taylor, S.R., Jang, Y., Sivan, A., Rhee, K., et al. (2024). Engineering tumor-colonizing *E. coli* Nissle 1917 for detection and treatment of colorectal neoplasia. Nat. Commun. *15*, 646. https://doi.org/10.1038/s41467-024-44776-4.
7. Swofford, C.A., Van Dessel, N., and Forbes, N.S. (2015). Quorum-sensing Salmonella selectively trigger protein expression within tumors. Proc. Natl. Acad. Sci. USA *112*, 3457–3462. https://doi.org/10.1073/pnas.1414558112.
8. Redenti, A., Im, J., Redenti, B., Li, F., Rouanne, M., Sheng, Z., Sun, W., Gurbatri, C.R., Huang, S., Komaranchath, M., et al. (2024). Probiotic neoantigen delivery vectors for precision cancer immunotherapy. Nature *635*, 453–461. https://doi.org/10.1038/s41586-024-08033-4.
9. Li, F., Yang, Z., Savage, T.M., Vincent, R.L., de Los Santos-Alexis, K., Ahn, A., Rouanne, M., Mariuzza, D.L., Danino, T., and Arpaia, N. (2024). Programmable bacteria synergize with PD-1 blockade to overcome cancer cell-intrinsic immune resistance mechanisms. Sci. Immunol. *9*, eadn9879. https://doi.org/10.1126/sciimmunol.adn9879.
10. de Oliveira Alves, N., Dalmasso, G., Nikitina, D., Vaysse, A., Ruez, R., Ledoux, L., Pedron, T., Bergsten, E., Boulard, O., Autier, L., et al. (2024). The

colibactin-producing Escherichia coli alters the tumor microenvironment to immunosuppressive lipid overload facilitating colorectal cancer progression and chemoresistance. Gut Microbes *16*, 2320291. https://doi.org/10.1080/19490976.2024.2320291.

11. Huang, X., Pan, J., Xu, F., Shao, B., Wang, Y., Guo, X., and Zhou, S. (2021). Bacteria-based cancer immunotherapy. Adv. Sci. *8*, 2003572. https://doi.org/10.1002/advs.202003572.

12. Nusse, R., and Clevers, H. (2017). Wnt/beta-catenin signaling, disease, and emerging therapeutic modalities. Cell *169*, 985–999. https://doi.org/10.1016/j.cell.2017.05.016.

13. de Sousa e Melo, F., Kurtova, A.V., Harnoss, J.M., Kljavin, N., Hoeck, J.D., Hung, J., Anderson, J.E., Storm, E.E., Modrusan, Z., Koeppen, H., et al. (2017). A distinct role for Lgr5(+) stem cells in primary and metastatic colon cancer. Nature *543*, 676–680. https://doi.org/10.1038/nature21713.

14. Derks, L.L.M., and van Boxtel, R. (2023). Stem cell mutations, associated cancer risk, and consequences for regenerative medicine. Cell Stem Cell *30*, 1421–1433. https://doi.org/10.1016/j.stem.2023.09.008.

15. Yan, K.S., Janda, C.Y., Chang, J., Zheng, G.X.Y., Larkin, K.A., Luca, V.C., Chia, L.A., Mah, A.T., Han, A., Terry, J.M., et al. (2017). Non-equivalence of Wnt and R-spondin ligands during Lgr5(+) intestinal stem-cell self-renewal. Nature *545*, 238–242. https://doi.org/10.1038/nature22313.

16. Cui, C., Zhang, T.T., Lin, Q., Huang, T.X., Rao, E.Y., Du, J.H., and Fu, L. (2024). WNT2 blockade augments antitumor immunity by attenuating myeloid-derived suppressor cells in colorectal cancer. MedComm–Oncology *3*, e70004. https://doi.org/10.1002/mog2.70004.

17. Bao, J., Zheng, J.J., and Wu, D. (2012). The structural basis of DKK-mediated inhibition of Wnt/LRP signaling. Sci. Signal. *5*, pe22. https://doi.org/10.1126/scisignal.2003028.

18. Mao, B., Wu, W., Davidson, G., Marhold, J., Li, M., Mechler, B.M., Delius, H., Hoppe, D., Stannek, P., Walter, C., et al. (2002). Kremen proteins are Dickkopf receptors that regulate Wnt/beta-catenin signalling. Nature *417*, 664–667. https://doi.org/10.1038/nature756.

19. Causeret, F., Sumia, I., and Pierani, A. (2016). Kremen1 and Dickkopf1 control cell survival in a Wnt-independent manner. Cell Death Differ. *23*, 323–332. https://doi.org/10.1038/cdd.2015.100.

20. Sun, Y., Zhang, X., Hang, D., Lau, H.C.H., Du, J., Liu, C., Xie, M., Pan, Y., Wang, L., Liang, C., et al. (2024). Integrative plasma and fecal metabolomics identify functional metabolites in adenoma-colorectal cancer progression and as early diagnostic biomarkers. Cancer Cell *42*, 1386–1400.e8. https://doi.org/10.1016/j.ccell.2024.07.005.

21. Coker, O.O., Liu, C., Wu, W.K.K., Wong, S.H., Jia, W., Sung, J.J.Y., and Yu, J. (2022). Altered gut metabolites and microbiota interactions are implicated in colorectal carcinogenesis and can be non-invasive diagnostic biomarkers. Microbiome *10*, 35. https://doi.org/10.1186/s40168-021-01208-5.

22. Bosch, S., Acharjee, A., Quraishi, M.N., Rojas, P., Bakkali, A., Jansen, E.E., Brizzio Brentar, M., Kuijvenhoven, J., Stokkers, P., Struys, E., et al. (2022). The potential of fecal microbiota and amino acids to detect and monitor patients with adenoma. Gut Microbes *14*, 2038863. https://doi.org/10.1080/19490976.2022.2038863.

23. Herrero Del Valle, A., Seip, B., Cervera-Marzal, I., Sacheau, G., Seefeldt, A.C., and Innis, C.A. (2020). Ornithine capture by a translating ribosome controls bacterial polyamine synthesis. Nat. Microbiol. *5*, 554–561. https://doi.org/10.1038/s41564-020-0669-1.

24. Ohlendorf, R., Li, N., Phi Van, V.D., Schwalm, M., Ke, Y., Dawson, M., Jiang, Y., Das, S., Stallings, B., Zheng, W.T., and Jasanoff, A. (2024). Imaging bioluminescence by detecting localized haemodynamic contrast from photosensitized vasculature. Nat. Biomed. Eng. *8*, 775–786. https://doi.org/10.1038/s41551-024-01210-w.

25. Schunk, S.J., Floege, J., Fliser, D., and Speer, T. (2021). WNT-beta-catenin signalling - a versatile player in kidney injury and repair. Nat. Rev. Nephrol. *17*, 172–184. https://doi.org/10.1038/s41581-020-00343-w.

26. Chun, S.K., Fortin, B.M., Fellows, R.C., Habowski, A.N., Verlande, A., Song, W.A., Mahieu, A.L., Lefebvre, A.E.Y.T., Sterrenberg, J.N., Velez, L.M., et al. (2022). Disruption of the circadian clock drives Apc loss of heterozygosity to accelerate colorectal cancer. Sci. Adv. *8*, eabo2389. https://doi.org/10.1126/sciadv.abo2389.

27. Erazo-Oliveras, A., Muñoz-Vega, M., Mlih, M., Thiriveedi, V., Salinas, M.L., Rivera-Rodríguez, J.M., Kim, E., Wright, R.C., Wang, X., Landrock, K.K., et al. (2023). Mutant APC reshapes Wnt signaling plasma membrane nanodomains by altering cholesterol levels via oncogenic beta-catenin. Nat. Commun. *14*, 4342. https://doi.org/10.1038/s41467-023-39640-w.

28. Li, B., Lee, C., Cadete, M., Zhu, H., Koike, Y., Hock, A., Wu, R.Y., Botts, S.R., Minich, A., Alganabi, M., et al. (2019). Impaired Wnt/beta-catenin pathway leads to dysfunction of intestinal regeneration during necrotizing enterocolitis. Cell Death Dis. *10*, 743. https://doi.org/10.1038/s41419-019-1987-1.

29. Deng, F., Peng, L., Li, Z., Tan, G., Liang, E., Chen, S., Zhao, X., and Zhi, F. (2018). YAP triggers the Wnt/beta-catenin signalling pathway and promotes enterocyte self-renewal, regeneration and tumorigenesis after DSS-induced injury. Cell Death Dis. *9*, 153. https://doi.org/10.1038/s41419-017-0244-8.

30. Farin, H.F., Van Es, J.H., and Clevers, H. (2012). Redundant sources of Wnt regulate intestinal stem cells and promote formation of Paneth cells. Gastroenterology *143*, 1518–1529.e7. https://doi.org/10.1053/j.gastro.2012.08.031.

31. Pan, Q., Zhong, S., Wang, H., Wang, X., Li, N., Li, Y., Zhang, G., Yuan, H., Lian, Y., Chen, Q., et al. (2021). The ZMYND8-regulated mevalonate pathway endows YAP-high intestinal cancer with metabolic vulnerability. Mol. Cell *81*, 2736–2751.e8. https://doi.org/10.1016/j.molcel.2021.04.009.

32. Ren, X., Liu, Q., Zhou, P., Zhou, T., Wang, D., Mei, Q., Flavell, R.A., Liu, Z., Li, M., Pan, W., and Zhu, S. (2024). DHX9 maintains epithelial homeostasis by restraining R-loop-mediated genomic instability in intestinal stem cells. Nat. Commun. *15*, 3080. https://doi.org/10.1038/s41467-024-47235-2.

33. Zebisch, M., Jackson, V.A., Zhao, Y., and Jones, E.Y. (2016). Structure of the dual-mode Wnt regulator Kremen1 and insight into ternary complex formation with LRP6 and Dickkopf. Structure *24*, 1599–1605. https://doi.org/10.1016/j.str.2016.06.020.

34. Liu, C., Kato, Y., Zhang, Z., Do, V.M., Yankner, B.A., and He, X. (1999). beta-Trcp couples beta-catenin phosphorylation-degradation and regulates Xenopus axis formation. Proc. Natl. Acad. Sci. USA *96*, 6273–6278. https://doi.org/10.1073/pnas.96.11.6273.

35. Lee, E.J., Jo, M., Rho, S.B., Park, K., Yoo, Y.N., Park, J., Chae, M., Zhang, W., and Lee, J.H. (2009). Dkk3, downregulated in cervical cancer, functions as a negative regulator of beta-catenin. Int. J. Cancer *124*, 287–297. https://doi.org/10.1002/ijc.23913.

36. Padhi, P., Abdalla, A., Schneider, B., Backes, N., Otto, A.A., Scheibe, I.J., Thomas, J.P., Khadse, G., Samidurai, M., Jochmans, A.K., et al. (2025). Bioengineered gut bacterium synthesizing levodopa alleviates motor deficits in models of Parkinson's disease. Cell Host Microbe *33*, 1837–1854.e13. https://doi.org/10.1016/j.chom.2025.10.005.

37. Sun, Y., Guo, Y., Liu, X., Liu, J., Sun, H., Li, Z., Wen, M., Jiang, S.N., Tan, W., and Zheng, J.H. (2023). Engineered oncolytic bacteria HCS1 exerts high immune stimulation and safety profiles for cancer therapy. Theranostics *13*, 5546–5560. https://doi.org/10.7150/thno.87340.

38. Zhang, X., Yu, D., Wu, D., Gao, X., Shao, F., Zhao, M., Wang, J., Ma, J., Wang, W., Qin, X., et al. (2023). Tissue-resident Lachnospiraceae family bacteria protect against colorectal carcinogenesis by promoting tumor immune surveillance. Cell Host Microbe *31*, 418–432.e8. https://doi.org/10.1016/j.chom.2023.01.013.

39. Rothemich, A., and Arthur, J.C. (2019). The azoxymethane/Il10 (-/-) model of colitis-associated cancer (CAC). Methods Mol. Biol. *1960*, 215–225. https://doi.org/10.1007/978-1-4939-9167-9_19.

40. Stirling, F., Naydich, A., Bramante, J., Barocio, R., Certo, M., Wellington, H., Redfield, E., O'Keefe, S., Gao, S., Cusolito, A., et al. (2020). Synthetic

cassettes for pH-mediated sensing, counting, and containment. Cell Rep. *30*, 3139–3148.e4. https://doi.org/10.1016/j.celrep.2020.02.033.

41. Chowdhury, S., Castro, S., Coker, C., Hinchliffe, T.E., Arpaia, N., and Danino, T. (2019). Programmable bacteria induce durable tumor regression and systemic antitumor immunity. Nat. Med. *25*, 1057–1063. https://doi.org/10.1038/s41591-019-0498-z.
42. Abedi, M.H., Yao, M.S., Mittelstein, D.R., Bar-Zion, A., Swift, M.B., Lee-Gosselin, A., Barturen-Larrea, P., Buss, M.T., and Shapiro, M.G. (2022). Ultrasound-controllable engineered bacteria for cancer immunotherapy. Nat. Commun. *13*, 1585. https://doi.org/10.1038/s41467-022-29065-2.
43. Qiao, L., Niu, L., Wang, Z., Deng, Z., Di, D., Ma, X., Zhou, Y., Kong, D., Wang, Q., Yin, J., et al. (2025). Engineered bacteria for near-infrared light-inducible expression of cancer therapeutics. Nat. Cancer *6*, 612–628. https://doi.org/10.1038/s43018-025-00932-3.
44. Zheng, J.H., Nguyen, V.H., Jiang, S.N., Park, S.H., Tan, W., Hong, S.H., Shin, M.G., Chung, I.J., Hong, Y., Bom, H.S., et al. (2017). Two-step enhanced cancer immunotherapy with engineered *Salmonella typhimurium* secreting heterologous flagellin. Sci. Transl. Med. *9*, eaak9537. https://doi.org/10.1126/scitranslmed.aak9537.
45. Liu, N.N., Jiao, N., Tan, J.C., Wang, Z., Wu, D., Wang, A.J., Chen, J., Tao, L., Zhou, C., Fang, W., et al. (2022). Multi-kingdom microbiota analyses identify bacterial-fungal interactions and biomarkers of colorectal cancer across cohorts. Nat. Microbiol. *7*, 238–250. https://doi.org/10.1038/s41564-021-01030-7.
46. Lloyd-Price, J., Arze, C., Ananthakrishnan, A.N., Schirmer, M., Avila-Pacheco, J., Poon, T.W., Andrews, E., Ajami, N.J., Bonham, K.S., Brislawn, C.J., et al. (2019). Multi-omics of the gut microbial ecosystem in inflammatory bowel diseases. Nature *569*, 655–662. https://doi.org/10.1038/s41586-019-1237-9.
47. Schirmer, M., Stražar, M., Avila-Pacheco, J., Rojas-Tapias, D.F., Brown, E.M., Temple, E., Deik, A., Bullock, K., Jeanfavre, S., Pierce, K., et al. (2024). Linking microbial genes to plasma and stool metabolites uncovers host-microbial interactions underlying ulcerative colitis disease course. Cell Host Microbe *32*, 209–226.e7. https://doi.org/10.1016/j.chom.2023.12.013.
48. Acebron, S.P., Karaulanov, E., Berger, B.S., Huang, Y.L., and Niehrs, C. (2014). Mitotic wnt signaling promotes protein stabilization and regulates cell size. Mol. Cell *54*, 663–674. https://doi.org/10.1016/j.molcel.2014.04.014.
49. Li, Q., Geng, S., Luo, H., Wang, W., Mo, Y.Q., Luo, Q., Wang, L., Song, G.B., Sheng, J.P., and Xu, B. (2024). Signaling pathways involved in colorectal cancer: pathogenesis and targeted therapy. Signal Transduct. Targeted Ther. *9*, 266. https://doi.org/10.1038/s41392-024-01953-7.
50. Zhan, T., Rindtorff, N., and Boutros, M. (2017). Wnt signaling in cancer. Oncogene *36*, 1461–1473. https://doi.org/10.1038/onc.2016.304.
51. Wang, L., Deng, K., Gong, L., Zhou, L., Sayed, S., Li, H., Sun, Q., Su, Z., Wang, Z., Liu, S., et al. (2020). Chlorquinaldol targets the beta-catenin and T-cell factor 4 complex and exerts anti-colorectal cancer activity. Pharmacol. Res. *159*, 104955. https://doi.org/10.1016/j.phrs.2020.104955.
52. Jung, Y.S., and Park, J.I. (2020). Wnt signaling in cancer: therapeutic targeting of Wnt signaling beyond beta-catenin and the destruction complex. Exp. Mol. Med. *52*, 183–191. https://doi.org/10.1038/s12276-020-0380-6.
53. Federico, G., Meister, M., Mathow, D., Heine, G.H., Moldenhauer, G., Popovic, Z.V., Nordström, V., Kopp-Schneider, A., Hielscher, T., Nelson, P.J., et al. (2016). Tubular Dickkopf-3 promotes the development of renal atrophy and fibrosis. JCI Insight *1*, e84916. https://doi.org/10.1172/jci.insight.84916.
54. Mao, B., Wu, W., Li, Y., Hoppe, D., Stannek, P., Glinka, A., and Niehrs, C. (2001). LDL-receptor-related protein 6 is a receptor for Dickkopf proteins. Nature *411*, 321–325. https://doi.org/10.1038/35077108.
55. Sehgal, P., Lanauze, C., Wang, X., Hayer, K.E., Torres-Diz, M., Leu, N.A., Sela, Y., Stanger, B.Z., Lengner, C.J., and Thomas-Tikhonenko, A. (2021). MYC hyperactivates Wnt signaling in APC/CTNNB1-mutated colorectal cancer cells through miR-92a-dependent repression of DKK3. Mol. Cancer Res. *19*, 2003–2014. https://doi.org/10.1158/1541-7786.mcr-21-0666.
56. Gurbatri, C.R., Lia, I., Vincent, R., Coker, C., Castro, S., Treuting, P.M., Hinchliffe, T.E., Arpaia, N., and Danino, T. (2020). Engineered probiotics for local tumor delivery of checkpoint blockade nanobodies. Sci. Transl. Med. *12*, eaax0876. https://doi.org/10.1126/scitranslmed.aax0876.
57. Leventhal, D.S., Sokolovska, A., Li, N., Plescia, C., Kolodziej, S.A., Gallant, C.W., Christmas, R., Gao, J.R., James, M.J., Abin-Fuentes, A., et al. (2020). Immunotherapy with engineered bacteria by targeting the STING pathway for anti-tumor immunity. Nat. Commun. *11*, 2739. https://doi.org/10.1038/s41467-020-16602-0.
58. He, G.W., Lin, L., DeMartino, J., Zheng, X., Staliarova, N., Dayton, T., Begthel, H., van de Wetering, W.J., Bodewes, E., van Zon, J., et al. (2022). Optimized human intestinal organoid model reveals interleukin-22-dependency of paneth cell formation. Cell Stem Cell *29*, 1333–1345.e6. https://doi.org/10.1016/j.stem.2022.08.002.
59. Lin, Q., Zhang, S., Zhang, J., Jin, Y., Chen, T., Lin, R., Lv, J., Xu, W., Wu, T., Tian, S., et al. (2025). Colonic epithelial-derived FGF1 drives intestinal stem cell commitment toward goblet cells to suppress inflammatory bowel disease. Nat. Commun. *16*, 3264. https://doi.org/10.1038/s41467-025-58644-2.
60. Mahe, M.M., Sundaram, N., Watson, C.L., Shroyer, N.F., and Helmrath, M.A. (2015). Establishment of human epithelial enteroids and colonoids from whole tissue and biopsy. J. Vis. Exp. *97*, 52483. https://doi.org/10.3791/52483.

STAR★METHODS

KEY RESOURCES TABLE

REAGENT or RESOURCE	SOURCE	IDENTIFIER
Antibodies		
DKK3	Proteintech	Cat#10365-1-AP; RRID: AB_2261615
β-catenin	Abmart	Cat#M24002; RRID: AB_2920853
Cyclin D1	Abclonal	Cat#A19038; RRID: AB_2862530
c-Myc	Abmart	Cat# T55150, RRID: AB_2934184
Axin2	Proteintech	Cat# 20540-1-AP, RRID: AB_10694569
β-actin	Santa Cruz	Cat# sc-47778, RRID: AB_626632
PCNA	Selleck	Cat# F0018, RRID: AB_3698093
Ki67	Abmart	Cat# TW0001, RRID: AB_3665293
Olfm4	Selleck	Cat# F0742, RRID: AB_3714526
Mouse anti-HA-Tag	Abmart	Cat# M20003, RRID: AB_2864345
Rabbit anti-HA-Tag	Abclonal	Cat#AE105; RRID: AB_2943030
Bacterial strains		
E.coli Nissle 1917	Biobw	Cat# 089890
E. coli DH5α	Lab Collection	N/A
EcO-sfGFP	This study	N/A
EcO-Rluc8	This study	N/A
EcD	This study	N/A
HCS1/empty	Lab Collection	N/A
HCS1/DKK3	This study	N/A
Chemicals, peptides, and recombinant proteins		
Arginine	Sinopharm	Cat# 62004034
Lysine	Sinopharm	Cat# 620167391
Histidine	Sinopharm	Cat# 62014234
Tryptophan	Sinopharm	Cat# 62023034
Cysteine	Sinopharm	Cat# 62007234
Adipic acid	Sinopharm	Cat# 40000761
DMEM medium	Gibco	Cat# C11995500BT
FBS	Gibco	Cat# 10270106
Penicillin-streptomycin	Gibco	Cat# 15140122
LB medium	BD	Cat# 244620
Coelenterazine	MedMol	Cat# S80797
Azoxymethane	Sigma-Aldrich	Cat# A5486
Dextran sulfate sodium	MP Biomedicals	Cat# 0216011080
anti-HA magnetic beads	Biolinkedin	Cat# L-1009
Trizol	Vazyme	Cat# R401-01
Organoid Growth Medium	Daxiang Biotech	Cat# OC105131
Matrigel	Biogenous	Cat# M315066
Critical commercial assays		
Human DKK3 ELISA Kit	Bioswamp	Cat# HM11360
Cell Counting Kit-8	Abbkine	BMU106-CN
HiScript II Q RT SuperMix kit	Vazyme	Cat# R223-01
SYBR Green qPCR Master Mix	Vazyme	Cat# Q711-02/03

(*Continued on next page*)

Continued		
REAGENT or RESOURCE	SOURCE	IDENTIFIER
Experimental models: cell lines		
Human: HCT116 cells	Chonnam National University	N/A
Human: HT29 cells	Bikeman Bio	Cat# 110811042
Mouse: MC38 cells	Chonnam National University	N/A
Experimental models: organisms/strains		
C57BL/6J mice	Gempharmatech	Cat# N000013
Apc$^{Min/+}$ mice	Gempharmatech	Cat# T001457
NCG mice	Gempharmatech	Cat# T001475
Lgr5$^{GFP-IRES-CreERT2}$; *tdTomato* mice	Gift from Dr. Shu, Zhu, USTC, China	N/A
IL10$^{-/-}$ mice	Gift from Dr. Jianfeng, Wu, XiaMen University	N/A
Oligonucleotides		
qPCR primer forward for *DKK3-HA*: CTCTGACCGAAGAAATGGCG	Sangon	N/A
qPCR primer reverse for *DKK3-HA*: ATGCGTAGTCCGGAACGTC	Sangon	N/A
qPCR primer forward for *Lrp5*: AAGGGTGCTGTGTACTGGAC	Sangon	N/A
qPCR primer reverse for *Lrp5*: AGAAGAGAACCTTACGGGACG	Sangon	N/A
qPCR primer forward for *Lrp6*: TTGTTGCTTTATGCAAACAGACG	Sangon	N/A
qPCR primer reverse for *Lrp6*: GTTCGTTTAATGGCTTCTTCGC	Sangon	N/A
qPCR primer forward for *Wnt3*: TGGAACTGTACCACCATAGATGAC	Sangon	N/A
qPCR primer reverse for *Wnt3*: ACACCAGCCGAGGCGATG	Sangon	N/A
qPCR primer forward for *Axin2*: GGACTGGGGAGCCTAAAGGT	Sangon	N/A
qPCR primer reverse for *Axin2*: AAGGAGGGACTCCATCTACGC	Sangon	N/A
qPCR primer forward for *Lgr5*: CCTACTCGAAGACTTACCCAGT	Sangon	N/A
qPCR primer reverse for *Lgr5*: GCATTGGGGTGAATGATAGCA	Sangon	N/A
qPCR primer forward for *Olfm4*: GCCACTTTCCAATTTCAC	Sangon	N/A
qPCR primer reverse for *Olfm4*: GAGCCTCTTCTCATACAC	Sangon	N/A
qPCR primer forward for *PCNA*: TTTGAGGCACGCCTGATCC	Sangon	N/A
qPCR primer reverse for *PCNA*: GGAGACGTGAGACGAGTCCAT	Sangon	N/A
qPCR primer forward for *Ki67*: GAGGAGAAACGCCAACCAAGAG	Sangon	N/A
qPCR primer reverse for *Ki67*: TTTGTCCTCGGTGGCGTTATCC	Sangon	N/A
qPCR primer forward for *c-Myc*: CACCACCAGCAGCGACTCT	Sangon	N/A
qPCR primer reverse for *c-Myc*: GGCACCTCTTGAGGACCAGT	Sangon	N/A
qPCR primer forward for *β-catenin*: AAAGCGGCTGTTAGTCACTGG	Sangon	N/A

(*Continued on next page*)

Continued		
REAGENT or RESOURCE	SOURCE	IDENTIFIER
qPCR primer reverse for *β-catenin*: CGAGTCATTGCATACTGTCCAT	Sangon	N/A
qPCR primer forward for *Cyclin D1*: CGATGCCAACCTCCTCAACG	Sangon	N/A
qPCR primer reverse for *Cyclin D1*: CCAGGTAGTTCATGGCCAGC	Sangon	N/A
qPCR primer forward for *Gapdh*: CATCACTGCCACCCAGAAGACTG	Sangon	N/A
qPCR primer reverse for *Gapdh*: ATGCCAGTGAGCTTCCCGTTCAG	Sangon	N/A
Deposited data		
Fecal metabolomics analysis	MetaboLights	MTBLS12971
RNA sequencing data	Gene Expression Omnibus (GEO)	GSE307572
Software and algorithms		
ImageJ	N/A	https://imagej.nih.gov/ij/
GraphPad prism	N/A	https://www.graphpad.com/
Recombinant DNA		
Plasmid: pJ23119-speFL-sfGFP	This study	N/A
Plasmid: pJ23119-speFL-Rluc8	This study	N/A
Plasmid: pJ23119-pelB-DKK3-HA	This study	N/A

EXPERIMENTAL MODEL AND STUDY PARTICIPANT DETAILS

Patients and samples

Tumor and adjacent normal tissues from 6 patients with colorectal cancer were obtained for immunofluorescent staining and establishing PDO and PDX models. This study collected stool samples (8−12 patients per cohort) from CRC patients, IBD patients and healthy controls for testing ornithine-responsive bacteria (detailed information in Table S1). Specimens were obtained under informed consent approved by the Medical Ethics Committee of Hunan University (HNU-SMYXLL-AP-660005). The patients provided informed consent under Institutional Review Board-approved protocols.

Mice

Apc$^{Min/+}$ mice (9–12 weeks-old) and C57BL/6J mice (6–8 weeks-old) were purchased from Gempharmatech (Jiangsu, China). All mice were maintained in specific-pathogen-free conditions with approval by the Animal Research Committee at Hunan University (HNU-IACUC-2021-103), China. Age- and sex-matched mice were used for all experiments. Both male and female mice were used. The food of all animals accorded with standard diet for rodents. The animals used in study were compliant with all relevant ethical regulations regarding animal research.

Note on sex of animals

This study utilized both female and male mice for tumor models and observed similar results in mice of both sexes. Preliminary findings suggested that there was no gender limitation for the application of the engineered bacteria in this study.

Bacterial strains and culture condition

During the process of plasmid construction and bacterial activation, all bacterial growth was conducted at 37°C with shaking at 200 rpm overnight in lysogeny broth (LB) medium or M9 minimal medium containing appropriate antibiotic (10 mg/mL chloramphenicol). All reagents used in this study were of analytical grade. The bacterial strains used in this study are listed in Table S2.

Tumor cell lines

Human CRC cell line HCT116, HT29 and mouse colon cancer cell line MC38 were cultured in Dulbecco's Modified Eagle's Medium (DMEM) supplemented with 10% heated-inactivated fetal bovine serum (FBS), 100 U/mL penicillin, and 100 μg/mL streptomycin (Gibco). These cells were maintained in a humidified atmosphere containing 5% CO_2 at 37°C. All cell lines were tested negative for mycoplasma contamination using a PCR-based detection kit.

METHOD DETAILS

Metabolomics and analysis

Fecal samples were collected from *Apc*$^{Min/+}$ mice at the 9th, 13th and 17th week and frozen at −80°C for later LC-MS analysis. The metabolomic data analysis was performed by Shanghai Luming Biological Technology (Shanghai, China). Briefly, for sample preparation, 30 mg of sample was added to a 1.5 mL Eppendorf tube with L-2-chlorophenylalanine (4 μg/mL) dissolved in methanol as internal standard, and the tube was vortexed for 10 s. Subsequently, 400 μL of ice-cold mixture of methanol and acetonitrile (4/1, vol/vol) was added, and the mixtures were vortexed for 2 min, and the whole samples were extracted by ultrasonic for 10 min in ice-water bath, stored at −40°C overnight. The extract was centrifuged at 4°C (12000 rpm) for 10 min. The 150 μL supernatants from each tube were collected using crystal syringes, filtered through 0.22 μm microfilters and transferred to LC vials. The vials were stored at −80°C until LC-MS analysis. QC samples were prepared by mixing aliquot of the all samples to be a pooled sample.

For sample detection, an ACQUITY UPLC I-Class plus (Waters Co., Milford, MA) fitted with Q-Exactive mass spectrometer equipped with heated electrospray ionization (ESI) source (Thermo Fisher Scientific, Waltham, MA) was used to analyze the metabolic profiling in both ESI positive and ESI negative ion modes. An ACQUITY UPLC HSS T3 column (1.8 μm, 2.1 × 100 mm) was employed in both positive and negative modes. The original LC-MS data were processed by software Progenesis QI V2.3 (Nonlinear, Dynamics, Newcastle, UK) for baseline filtering, peak identification, integral, retention time correction, peak alignment, and normalization. Variable Importance of Projection (VIP) values obtained from the OPLS-DA model were used to rank the overall contribution of each variable to group discrimination. A two-tailed Student's *t* test was further used to verify whether the metabolites of difference between groups were significant.

Plasmids construction

The construction of plasmids was conducted in *E. coli* strain DH5α in accordance with standard procedures. The ornithine sensing system was derived from the ColE1 backbone with the constitutive promoter J23119 as the origin promoter. To construct the ornithine sensor and input visual signal, a region containing speFL, the speFL-speF intergenic region and the first three codons of speF, amplified from *E. coli* K12 genome, was fused with the sfGFP gene, which was optimized and synthesized by Tsingke Biotechnology (Beijing, China), then the fragment linked with ColE1 backbone to obtain pJ23119-speFL-sfGFP. To optimize the ornithine sensor pJ23119-speFL-sfGFP to avoid impurities interference in feces, sfGFP gene was substituted with Rluc8 gene, which was amplified from the pBAD-Rluc8 plasmid, termed as pJ23119-speFL-Rluc8. The DKK3 gene was optimized and synthesized by Tsingke Biotechnology, and fused with secretory peptide *pelB*, then integrated into ColE1 backbone with J23119 promoter, termed as pJ23119-pelB-DKK3-HA. All of the three plasmids then transformed into EcN respectively, termed as EcO-sfGFP, EcO-Rluc8 and EcD.

Growth curve and determination of fluorescence intensity

Single colony was cultured overnight and harvested, followed by washing and diluting with PBS. Subsequently, 3×10^8 CFU were inoculated into 30 mL LB medium containing different concentrations of ornithine in 200 mL flask and incubated with 200 rpm shaking at 37°C. The OD_{600} of each sample was measured at 0, 2, 4, 6, 8, 12, and 24 h using a UV spectrophotometer (UVmini-1280, Shimadzu, Japan). The overnight cultured bacteria were inoculated into LB medium or M9 medium containing different concentrations of ornithine with a ratio of 1:100 for 6 h and the fluorescence intensity was measured.

For assessment the specific of EcO-sfGFP, the overnight cultured bacteria were inoculated into M9 medium with 1:100 ratio, and different concentration of arginine, lysine, histidine, tryptophan, tyrosine, cysteine and adipic acid were added, then co-culture for 24 h and the fluorescence intensity was measured. A microplate reader (SpectraMax M2/M2e, Molecular Devices, CA) was used to measure the OD_{600} values (absorbance at 600 nm) and fluorescence values (excitation at 488 nm, emission at 510 nm). The Fluo/OD_{600} ratio showed the reporter gene expression intensity.

Bioluminescence imaging

EcN transformed with ornithine-Rluc8 was cultured overnight in M9 medium, then inoculated to fresh M9 medium with 1×10^9 CFU each 100 μL containing 0, 2, 5, 10 mM ornithine. After shaking for 6 h, add 100 μL into 96 well ELISA plate and before imaging, 1 μL of 2 mg/mL coelenterazine dissolved in methanol was diluted in PBS at a final volume of 10 μL and added into samples. Bioluminescence imaging was performed using the *In Vivo* Imaging System (SpectrumCT 2, PerkinElmer, MA). Imaging signals were quantified in units of maximum photons per second per centimeter square per steradian (p/s/cm^2/sr) within the region of interest.

For non-invasive screening via feces, fecal samples of healthy control, *Apc*$^{Min/+}$, DSS-treated, *IL10*$^{-/-}$ and aged mice were collected and dissolved with M9 medium to a final concentration of 1 g/mL, then diluted and shaking with 1×10^9 CFU bacteria in 100 μL for 6 h, then bioluminescence imaging was detected as above.

For assessment of EcO-Rluc8 in response to ornithine in patients, fecal samples from random 12 healthy individuals and CRC patients, and 8 healthy individuals and IBD patients were collected, and dissolved in M9 medium to 1 g/mL and vortex, then 0.5% of which was co-cultured with 1×10^9 EcO-Rluc8 for 6 h, and 100 μL supernatant was added into 96 well ELISA plate for detection.

Cell experiment

HCT116 and HT29 cells were used to assess the antitumor effects of DKK3. 10 mL bacterial supernatant were concentrated with ultrafiltration tube into 1 mL, and then added at a final concentration of 1 mg/mL for incubation. Proliferation of HCT116 as measured according to Cell Counting Kit-8 (CCK-8, BMU106-CN, Abbkine, Wuhan, China) protocol. For colony formation assay, 1,000 cells per well were seeded into a 6-well plate. Bacterial supernatant was supplied every 2 days, after incubation for 6 days, colonies were fixed by 4% PFA and stained by crystal violet, then the colony numbers were analyzed.

Tumor models and treatment

In the subcutaneous tumor model, 6–8 weeks-old C57BL/6J mice were used. The MC38 cells were subcutaneously implanted into the right flank of the mice at a density of 1×10^6 each. When the tumors reached a size of 100–120 mm^3, mice were randomly assigned and intravenously (i.v.) injected with PBS or 1×10^7 HCS1 strain. The tumor sizes and weight of mice were measured and recorded every 3 days until the tumor volumes reached 1,500 mm^3. The tumors were measured with a caliper, and the tumor volume was calculated using the following formula: $(L \times W \times H)/2$, where L, W, and H are the length, width, and height, respectively, of the tumor in millimeters. Mice with tumor volumes over 1,500 mm^3 were euthanized.

For AOM/DSS-induced colorectal cancer tumor model, 6–8 weeks-old C57BL/6J mice were intraperitoneally (i.p.) injected with azoxymethane (AOM, 10 mg/kg body weight) as described previously. The second day, mice were provided water with 2.5% DSS for a week, and then instead of water for two weeks. Mice were given intragastric administration with 200 μL sterile PBS or 1×10^9 cells of bacteria in 200 μL dilution twice a week when provided water. Mice underwent three rounds of treatment with DSS and water and were sacrificed at the 22nd week.

For AOM/*IL10*$^{-/-}$ model, 10-week-old *IL10*$^{-/-}$ mice were i.p. injected with AOM (10 mg/kg body weight) every five weeks, mice were given intragastric administration with 200 μL sterile PBS or 1×10^9 cells of bacteria in 200 μL dilution twice a week till 20th week.

For *Apc*$^{Min/+}$ mice spontaneous colorectal cancer model, 9 or 12-week-old *Apc*$^{Min/+}$ mice were given intragastric administration with 200 μL sterile PBS or 1×10^9 cells of bacteria in 200 μL dilution twice a week till the 20th week. Tumor nodules and area in small intestine of *Apc*$^{Min/+}$ mice were analyzed.

Pull-down assay

DKK3-HA was expressed in EcD and obtained after ultrasonic treatment. For pull-down assays, DKK3-HA protein was incubated with anti-HA magnetic beads at 4°C for 12 hours, after washing with IP lysis buffer, then the beads were incubated with cell lysate from HCT116 cells at 4°C for 12 hours. After incubation, beads were washed four times with IP lysis buffer, and 30 μL 2× loading buffer was added to boil for 5 min. Samples were subjected to SDS-PAGE gel and analyzed with immunoblotting.

Western blot

To assess the expression of DKK3 in EcN, the EcN transformed with pJ23119-empty and pJ23119-DKK3 were cultured overnight and then inoculated to fresh LB medium with a ratio of 1:100 for 6 h incubation with shaking. The 1×10^8 total bacterial culture and supernatant were collected respectively. The samples were subjected to gel electrophoresis. The proteins in the gel were then transferred onto a nitrocellulose membrane, which was then subjected to three approximately 10-min washes with TBST buffer (TBS with 0.1% Tween 20). After washing, 10 mL of skim milk for blocking, and the membrane was incubated with a primary DKK3 antibody for overnight at 4°C. The membrane was then washed three times with TBST and incubated with goat anti-rabbit IgG conjugated to horseradish peroxidase (HRP, diluted at 1: 10,000) as the secondary antibody for 2 h at room temperature. The membrane was then washed three times with TBST and imaged.

For Western blot analysis of tumor tissues, the tissues were homogenized and lysed in RIPA buffer supplemented with protease inhibitor cocktail at a 1:100 dilution, followed by incubation on ice for approximately 30 min. The protein concentration was determined using a NanoDrop (Thermofisher, Waltham, MA). Approximately 100 μg of total protein was used for Western blot following standard conditions with primary antibodies (diluted at 1:1000) against poly β-catenin, Cyclin D1, c-Myc, Axin2 and β-actin. The membrane was then washed three times with TBST and incubated with goat anti-rabbit IgG or goat anti-mouse IgG conjugated to horseradish peroxidase (HRP, diluted at 1: 10,000) as the secondary antibody for 2 h at room temperature. The detection was performed using an ECL plus Western blot detection system (Sagecreation Mini chemi 910, Sinsage, Beijing, China), and the intensity was quantitatively analyzed using the ImageJ software.

Quantification of DKK3

To quantify the DKK3 level, culture supernatants of 1×10^8 CFU EcN and EcD in LB were collected, and the tissue lysates of *Apc*$^{Min/+}$ mice treated with EcN and EcD were measured with a DKK3 ELISA kit following the manufacturer's protocol.

Plasmid stability assessment

In vitro, EcN and EcD were subcultured for 3 and 7 days in LB medium without antibiotics, then diluted and spotted on LB plates with or without chloramphenicol, plasmid retention (%) = (colony counts on chloramphenicol plate/colony counts in non-antibiotic plate) × 100%. To assess the potential horizontal gene transfer of EcD, VNP20009/lux strain was co-culture with EcD for 7 days, and then the culture was spread on plates with kanamycin, chloramphenicol, or plates containing both kanamycin and

chloramphenicol. *In vivo*, mice were fed with EcN harboring pJ23119-pelB-DKK3-HA (ChlR) and pBAD-empty (AmpR) plasmids, and then we counted bacteria in feces on ampicillin and ampicillin-chloramphenicol plates.

To confirm the gene consistency of engineered bacteria *in vivo*, colonies were selected randomly and PCR amplified using specific primers (F: 5′-GGAACCTCTTACGTGCCGATCAA-3′, R: 5′-CTTTGAGTGAGCTGATACCGCTCGC-3′). And the bacteria on the plate were washed off with sterile PBS and centrifuged, then resuspended with PBS, and boiled with 5× loading buffer. Samples were analyzed by Western blot with anti-DKK3 antibody.

RNA-sequencing and analysis

Transcriptome sequencing was conducted by JMDNA Bio-Medical Technology (Shanghai, China). Briefly, the total RNA was extracted with the Trans-TransZol Up Plus RNA kit (ER501-01-V2, TransGen BioTech, Beijing, China). Agarose gel electrophoresis and NanoDrop detection were used to quantify and inspect the extracted RNA. After the detection, usable RNA of ≥50 ng/μL and ≥2 μg was finally obtained for subsequent library construction. Hieff NGS®Ultima Dual-mode mRNA Library Prep Kit (12309ES96, Yeasen, Shanghai, China) was used for library building. Briefly summarized as the following steps: (1) using magnetic beads with poly T to capture and enrich mRNA; (2) breaking the captured mRNA into short segments; (3) synthesizing cDNA; (4) connecting adapters; (5) purifying and fragment screening the connected product by using the purified sorting magnetic beads provided by the kit; (6) using the index primer matched with the kit to amplify the connected product, and then using the purification and sorting magnetic beads provided by the kit to purify and enrich the amplified product; (7) Finally, MGI DNBSEQ-T7 was used for sequencing using the PE150 mode. Raw reads in fastq format were initially processed using fastp, and low-quality reads were removed to obtain clean reads, which were retained for subsequent analyses. The clean reads were aligned to the reference genome using HISAT. The FPKM for each gene was calculated, and the read counts of each gene were obtained using HTSeq-count. Differential expression analysis was performed using DESeq2 with Q value <0.05 and foldchange >2 set as thresholds for significant differences in gene expression. R (v 3.2.0) was used to analyze.

Colonization of EcD in mice

To assess the colonization kinetic of EcN, single dose of 1×10^8, 1×10^9, 1×10^{10} CFU were gavage to WT mice, fecal samples at 6 h, 24 h, 3 days and 7 days were dissolved with PBS and diluted, then plated for colony enumeration. To investigate the biodistribution of EcD *in vivo*, the healthy male C57BL/6 mice were oral administrated with 1×10^9 EcD, blood, heart, lung, kidney, liver, spleen, duodenum, colon and feces were collected at 6, 24 h, 3 and 7 days post gavage. The tissues were homogenized and serial diluted before plating for colony counts. Following 12-h incubation at 37°C, colonies were determined. In $Apc^{Min/+}$ mice, tissue counting of liver, spleen, duodenum, cecum, colon and feces were performed in mice at early and late stage as above.

Immunofluorescent (IF) staining

The small intestine tissues were isolated, fixed in 4% paraformaldehyde (PFA) overnight, and washed with PBS. Cryoprotection was performed by incubating the tissues in 30% sucrose solution overnight. Afterward, the tissues were embedded in optimal cutting temperature (OCT) compound and sectioned into 6 μm-thin slices. Patient-derived CRC organoids were cultured and fixed with 4% paraformaldehyde for 30 min. Organoids were then incubated at 4°C overnight with 30% sucrose, followed by incubated with OCT. Compound at −20°C overnight and sectioned.

For IF staining, primary antibodies against PCNA, Ki67, DKK3, CyclinD1, Olfm4, β-catenin, and HA-tag were used, nuclei were counterstained with DAPI. Fluorescent images were captured with laser scanning confocal microscopy (LSM 980, Zeiss, Oberkochen, Germany).

Histopathology

The 5 μm-thick paraffin sections were deparaffinized and rehydrated using xylene and a descending ethanol gradient (100%, 95%, 85% and 75%). For hematoxylin & eosin (H&E) staining, slides were staining in hematoxylin for 6 min and washed in water. Then tissues were stained with eosin for 3 min and washed. Slides were transferred to xylene for 1 min. Slides were mounted and images were acquired by a Panthera upright compound microscope (Panthera S, Motic, Xiamen, China).

Hematological assessments

Healthy C57BL/6 were oral administrated with 1×10^9 EcN and EcD, plasma was collected with anti-coagulant tubes at 6 h and 4 weeks post-gavage, and complete blood was analyzed by auto hematology analyzer (BC-5130, Mindray, Shenzhen, China). And clinical chemistry parameters of serum, including aspartate aminotransferase (AST) and alanine aminotransferase (ALT) were analyzed by chemistry analyzer (BS-830, Mindray).

RNA isolation and RT-qPCR

Total RNA was isolated from cells or mouse tissues using the TRIzol reagent (Invitrogen) according to the manufacturer's instructions. For cDNA synthesis, 500 ng of total RNA was reverse transcribed using the HiScript II Q RT SuperMix kit. Quantitative reverse transcription polymerase chain reaction (RT-qPCR) was performed to analyze the expression levels of specific genes. The RT-qPCR reactions were conducted using the QuantStudio 1 real-time PCR system (Thermo) with a 10 μL reaction volume containing SYBR

Green qPCR Master Mix. The relative expression levels of the target genes were determined using the comparative CT (cycle threshold) method. The expression data were normalized to the internal control Gapdh. The qPCR primers used in this study are provided in the STAR Methods.

Organoid culture and treatment

Human colorectal crypts were isolated using an optimized protocol adapted from established methods.[58–60] Briefly, a 1 cm segment human colon biopsy samples were dissected and thoroughly washed with ice-cold phosphate-buffered saline (PBS) to eliminate residual fecal material. The tissues were then longitudinally incised and sectioned into small fragments and then washed with chilled PBS to remove cellular debris. Next, the samples were incubated in 10 mM EDTA (prepared in PBS) at 4°C for 30 min under gentle agitation for dissociation. Following this, vigorous mechanical shaking was applied to dislodge crypt structures, and the resulting supernatant was filtered through a 70 μm cell strainer to exclude larger tissue remnants. The crypt-containing filtrate was then centrifuged at 300 g for 2 min to pellet the crypts, which were then resuspended in PBS and quantified.

For organoid culture, a defined number of crypts were centrifuged at 300 g for 2 min and resuspended in Organoid Growth Medium at a density of 40 crypts per 15 μL. This suspension was combined with Matrigel in a 1:1 ratio, and 25 μL of the mixture was plated per well in a 48-well plate. After polymerization at 37°C under 5% CO_2 for 30 min, each well was supplemented with 200 μL of Organoid Growth Medium containing 1× penicillin-streptomycin (Invitrogen, California, CA). All procedures were reviewed and approved by the Medical Ethics Committee of Hunan University (HNU-SMYXLL-AP-660005).

For treatment, DKK3 secreted from EcD was re-suspended in the DMEM medium containing 1% penicillin/streptomycin, and refreshed every 48 h until the 6th day. Organoid formation and morphological features, including bud counts, were monitored and documented using an Invitrogen Evos XL Core Imaging System.

Colorectal cancer patient-derived xenograft (PDX) model

After subcutaneously culturing and expanding the patient-derived colorectal tumors in NCG mice for one generation, the tumors were collected and cut into small pieces of approximately $2 \times 2 \times 2$ mm^3, and then subcutaneously transplanted into the right flank of 4-week-old male NCG mice. Twenty-five days after transplantation, mice received a single intravenous injection of 5×10^7 CFU HCS1/empty or HCS1/DKK3. Tumor volumes were monitored and tumor tissues were collected for photography at 9 days post treatments.

QUANTIFICATION AND STATISTICAL ANALYSIS

Statistical analysis

Statistical analyses and graphical output of the data were performed with the computer program Prism (GraphPad Prism 9.0). Survival analysis was performed using the Kaplan-Meier method and the log rank test. Statistical significance was determined by an unpaired two-tailed *t* test; n.s. $p > 0.05$, *$p \leq 0.05$, **$p \leq 0.01$, ***$p \leq 0.001$, and ****$p \leq 0.0001$. All data are reported as the means ± SEM.

Cell Reports Medicine, Volume *7*

Supplemental information

Synthetically engineered probiotic suppresses colorectal cancer via inhibition of Wnt/β-catenin signaling

Yujie Sun (孙妤婕), Yu Zhao (赵宇), Kang Han (韩康), Wulong Hu, Guofan Peng, Shengen Yi, Jun Zhou (周俊), and Jin Hai Zheng (郑金海)

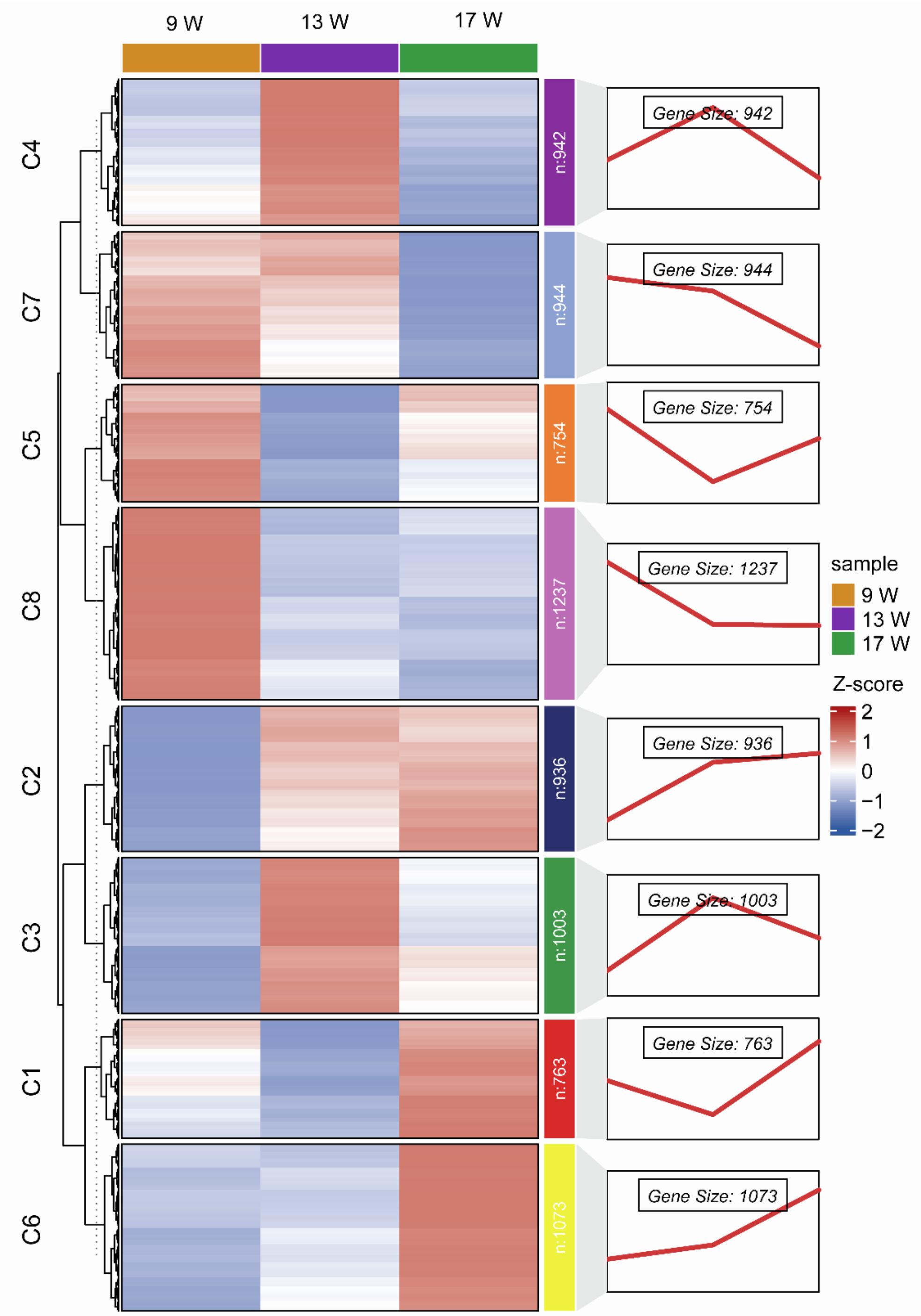

Figure S1. Heatmap cluster for visualizing clustered metabolite of *Apc*$^{Min/+}$ mice stools in time course. Related to Figure 1.

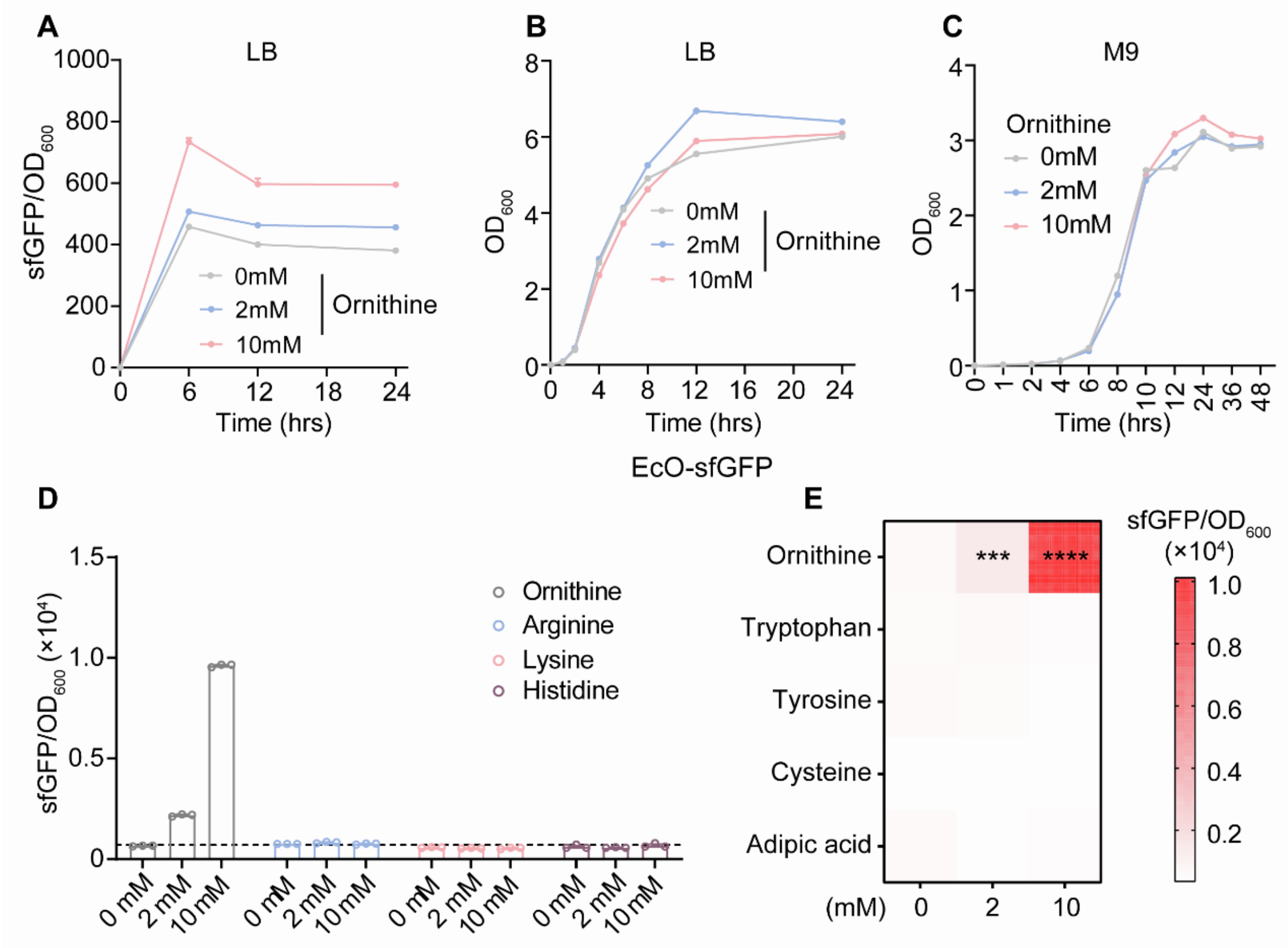

Figure S2. The profiling of EcO-sfGFP and its specificity *in vitro*. Related to Figure 1. (A) Fluorescence intensity of with ornithine responsive plasmid treated with 0, 2, 10 mM ornithine within 24 hours in LB medium. (B and C) Growth curve of engineered bacteria EcO-sfGFP with different ornithine concentrations in LB medium (B) and M9 medium (C). (D) Fluorescence intensity of EcO-sfGFP with arginine, lysine and histidine supplementation. (E) Heatmap of fluorescence intensity of EcO-sfGFP co-culture with tryptophan, tyrosine, cysteine and adipic acid. Statistical significance determined using an unpaired t test; *** $P \leq 0.001$, and **** $P \leq 0.0001$.

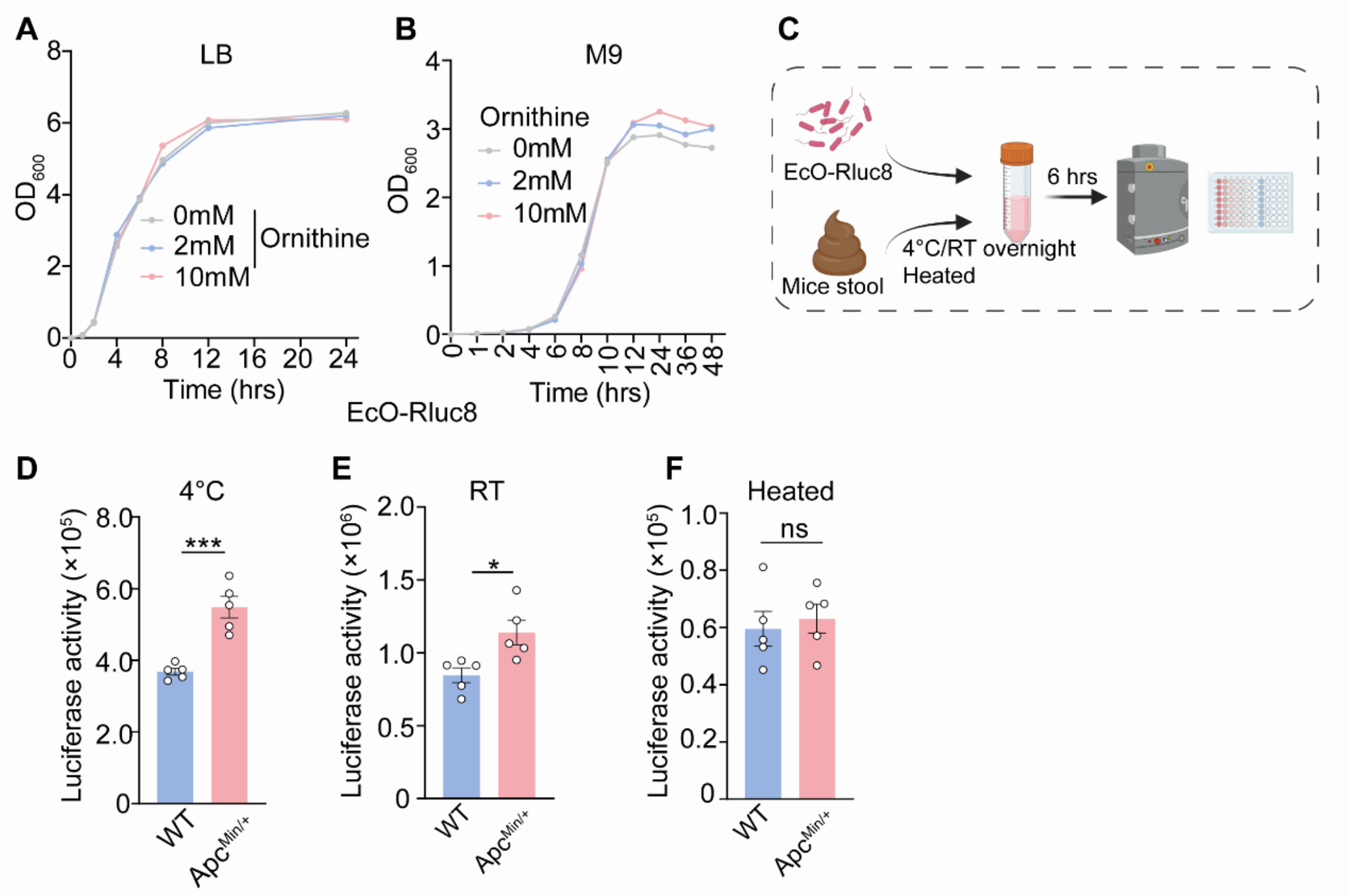

Figure S3. The profiling of EcO-Rluc8 with ornithine. Related to Figure 2. (A) Growth curve of engineered bacteria EcO-Rluc8 with different ornithine concentrations in LB medium. (B) Growth curve of engineered bacteria EcO-Rluc8 with different ornithine concentrations in M9 medium within 24 hours. (C) Schematic design of the efficiency of EcO-Rluc8. (D-F) Bioluminescence signals of EcO-Rluc8 exposure with stools deal with 4°C (D), room temperature (E) overnight and heated (F). Data are presented as mean ± SEM. Statistical significance determined using an unpaired t test; ns > 0.05, *$P \leq 0.05$ and *** $P \leq 0.001$.

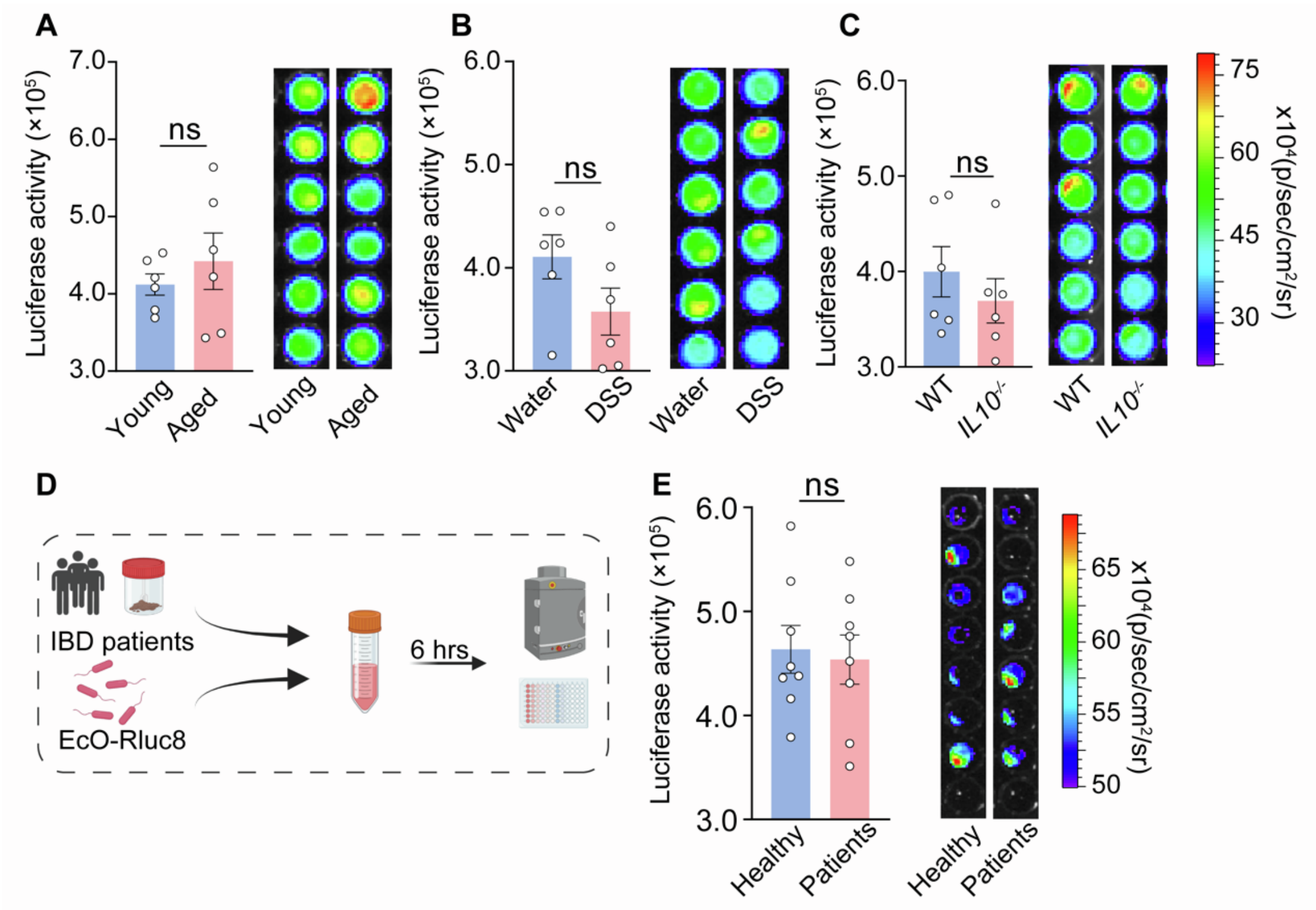

Figure S4. Specificity of EcO-Rluc8 in multiple intestinal disease models. Related to Figure 2. (A to C) Quantitative bioluminescence signals and images after co-incubating bacteria with stools from aged mice (A), DSS-treated (B), *IL10*$^{-/-}$ mice (C) and corresponding control groups (n = 6). (D) Schematic diagram of the efficiency of EcO-Rluc8 responsive to stools from IBD patients and healthy individuals. (E) Quantitative bioluminescence signals and images of co-culture EcO-Rluc8 with stools from IBD patients (n = 8). Data are presented as mean ± SEM. Statistical significance determined using an unpaired t test. ns, $P > 0.05$.

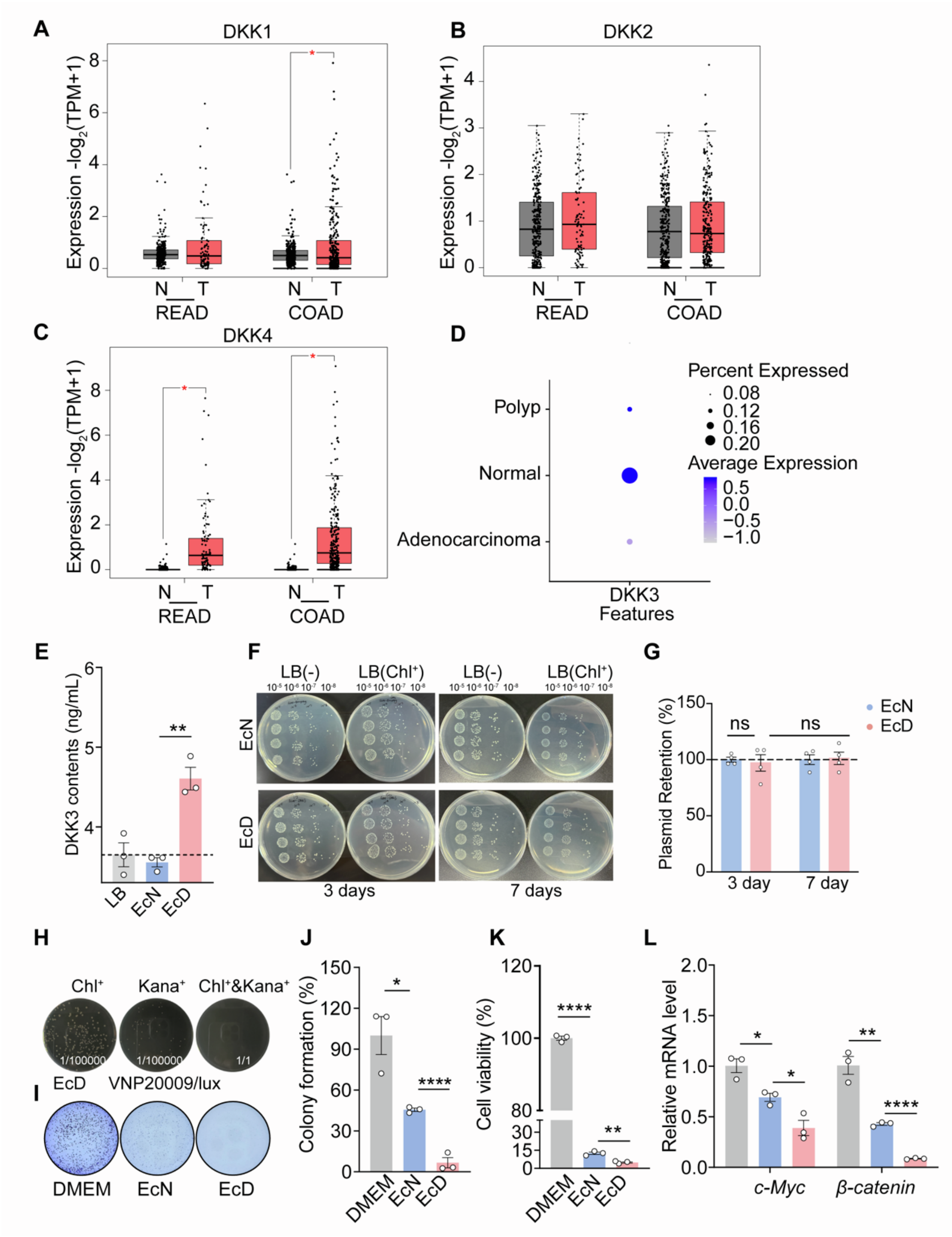

Figure S5. Expression of DKKs from the GEPIA database in colorectal cancer and the profiling of EcD *in vitro*. Related to Figure 3. (A to C) The mRNA level of DKK1 (A), DKK2 (B), and DKK4 (C) in colorectal cancer patients. READ (num(N)= 318, num(T) = 92), COAD (num(N) = 349, num(T) = 275). N, normal, T, tumor. (D) The expression of DKK3 in CRC patients within scRNA-seq analysis (GSE201349). (E) The contents of DKK3 in bacterial supernatant with $1{\times}10^{8}$ CFU bacteria. (F and G) Plasmid retention ratio of EcN and EcD *in vitro*. (H) The potential ability of horizontal gene transfer of EcD. (I) Representative images with crystal violet staining of HT29 cells with DKK3 co-incubation. (J and K) Colony formation

ratio (J) and cell viability (K) of HT29 treated with DKK3. (L) Quantification of mRNA levels of HT29 treated with DKK3. Statistical significance determined using an unpaired t test; ns, $P > 0.05$, *$P \leq 0.05$, ** $P \leq 0.01$ and **** $P \leq 0.0001$.

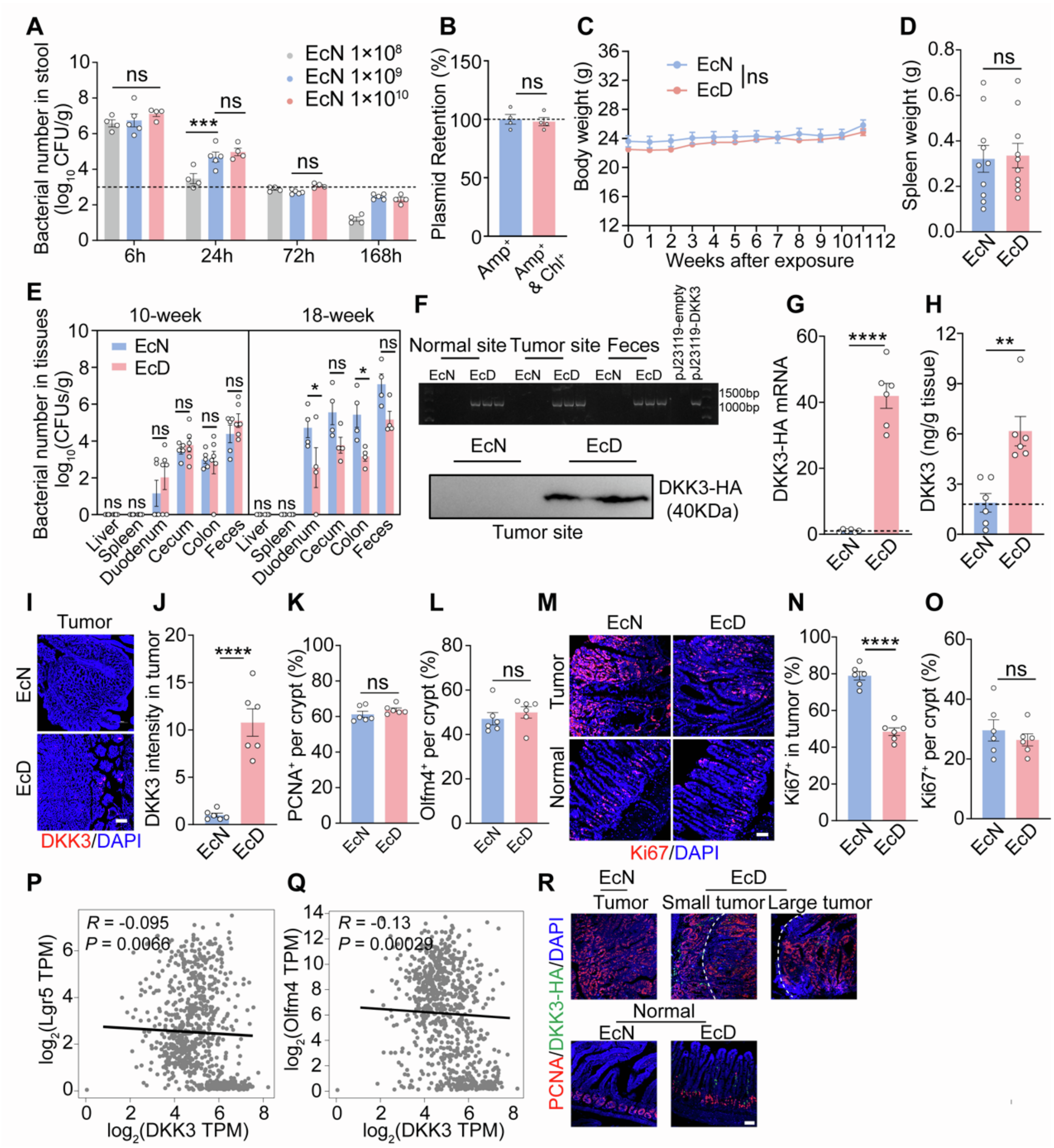

Figure S6. The stability of EcD *in vivo* and the effects of ISCs proliferation within EcD administration. Related to Figure 4. (A) Fecal colonization kinetics of EcN with single dose administration. (B) Plasmid stability of pJ23119-pelB-DKK3-HA in gut. (C and D) Body weight and spleen weight change in $Apc^{Min/+}$ mice with bacteria exposure. (E) Bacterial colonization after administration in 10- and 18-week-old $Apc^{Min/+}$ mice. (F) PCR and Western blot assays of engineered bacteria selected from related tissues. (G) The mRNA levels of DKK3-HA in tumors with EcD treatment. (H) Concentrations of DKK3 in tumor tissues. (I and J) Immunofluorescent staining (I) and signal intensity (J) of DKK3 in tumor tissues of small intestines. (K and L) Immunofluorescence signal quantification of PCNA (K) and Olfm4 (L) in crypts. (M to O) Immunofluorescent staining (M) and signal intensity with Ki67 in tumor tissues (N) and normal tissues (O) of small intestines. (P and Q) Correlation analysis between *DKK3* and *Lgr5* (P) and *Olfm4* (Q) in CRC patients using TCGA database. A tendency line was added based on the value of “*R*”. (R) Immunofluorescent

co-staining of PCNA and HA-tag in small intestines of *Apc*$^{Min/+}$ mice with EcD treatment. Data are presented as mean ± SEM. Statistical significance determined using an unpaired t test. ns, $P > 0.05$, *$P \leq 0.05$, ** $P \leq 0.01$ and **** $P \leq 0.0001$.

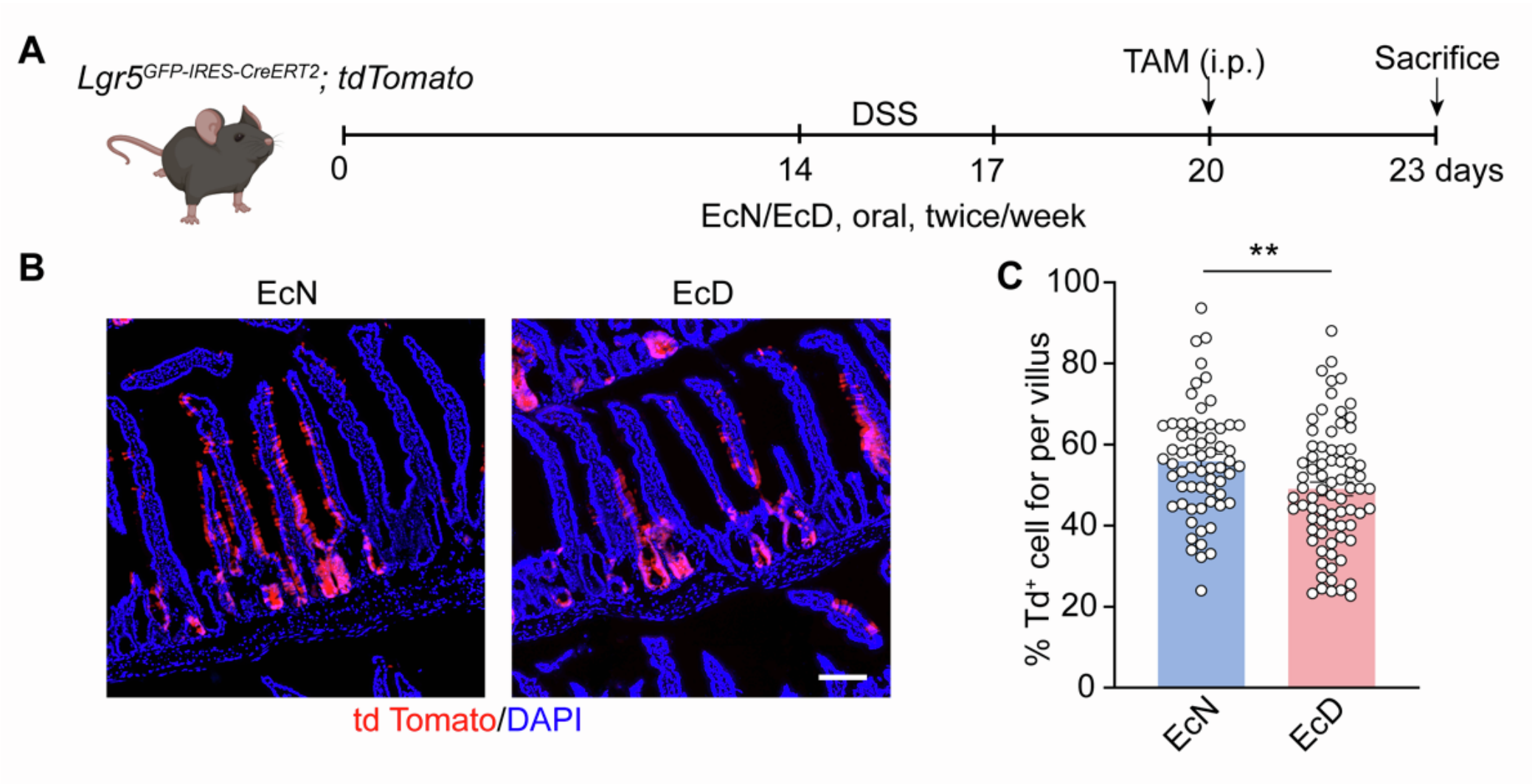

Figure S7. ISC proliferation in *Lgr5$^{GFP\text{-}CreERT2}$; tdTomato* mice with EcD treated. Related to Figure 4. (A) Experimental scheme of DSS and EcD treatment. (B) Representative images showing Lgr5-derived progeny (red) in small intestine after TAM treatment. (C) Quantification of tdTomato positive cell per villus with EcN and EcD treated. Data are presented as mean ± SEM. Statistical significance determined using an unpaired t test, ** $P \leq 0.01$.

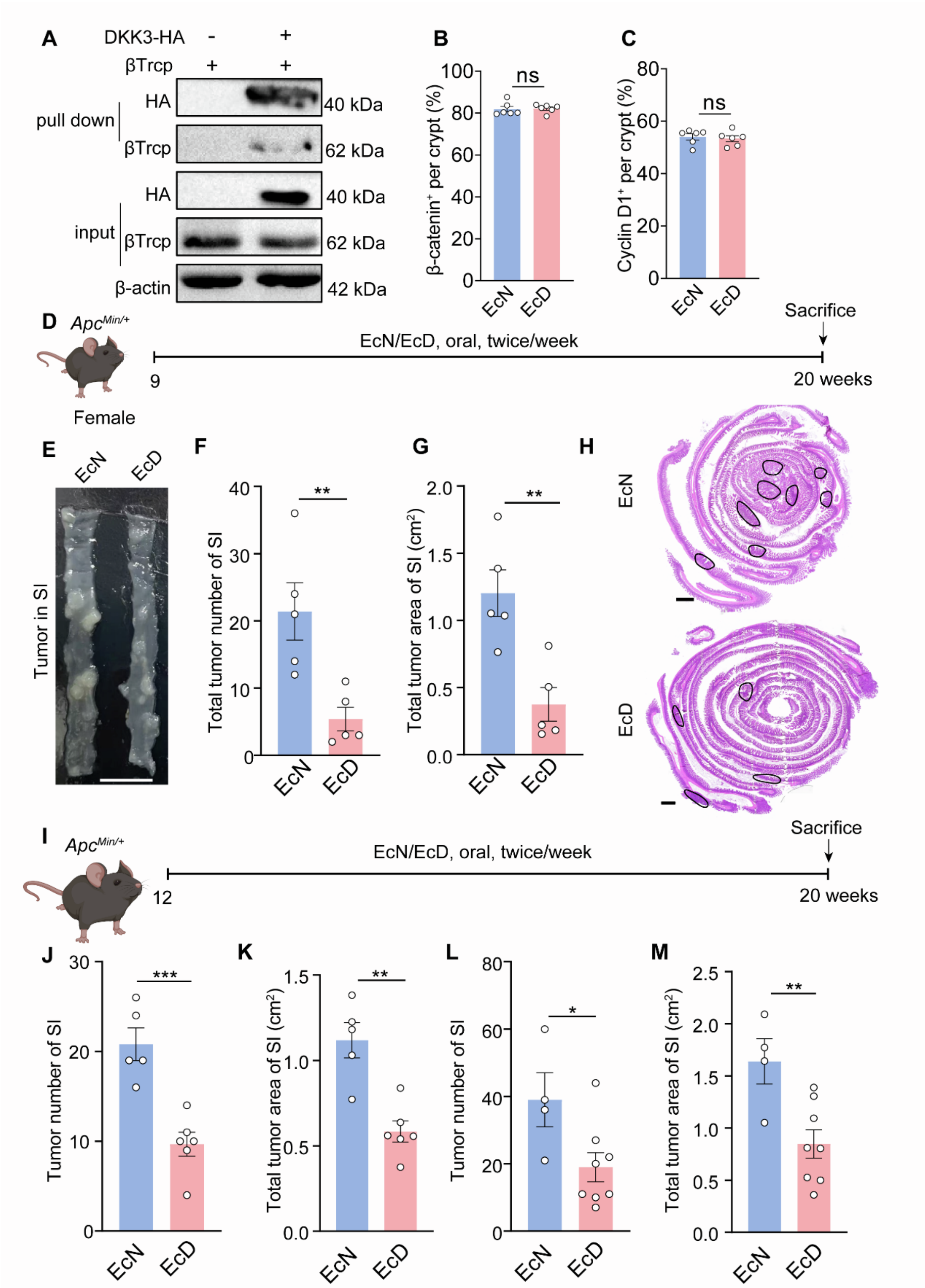

Figure S8. Effects of Wnt/β-catenin signaling within EcD administration. Related to Figure 5. (A) The interaction of DKK3-HA secreted from EcD with the βTrcp protein was validated by pull-down assay in HCT116 cells. (B and C) Immunofluorescent signal intensity analysis of β-catenin (B) and Cyclin D1 (C) in normal tissues of small intestines of *Apc*$^{Min/+}$ mice with EcD treatment. (D) Experimental design. The 9-week-old female *Apc*$^{Min/+}$ mice were randomly grouped

and oral administrated with EcN or EcD twice a week till the 20th week. The tumor numbers were counted and tumor tissues were collected. (E) At the 20th week, mice were sacrificed, and intestinal tissues were excised and counted, scale bar = 2 cm. (F and G) Analysis tumor numbers and total tumor areas in small intestine, n = 5. (H) Representative H&E-stained histological images of small intestines with EcN and EcD treated mice, scale bar = 1 mm. (I) Experimental design. The 12-week-old male and female $Apc^{Min/+}$ mice were divided randomly and oral administrated with EcN or EcD twice a week till the 20th week. The tumor numbers were counted. (J to M) At the 20th week, male (J and K) and female (L and M) mice were sacrificed, tumors from small intestine were counted and the total tumor areas were analyzed. Data are presented as mean ± SEM. Statistical significance determined using an unpaired t test. ns, $P > 0.05$, *$P \leq 0.05$, ** $P \leq 0.01$ and *** $P \leq 0.001$.

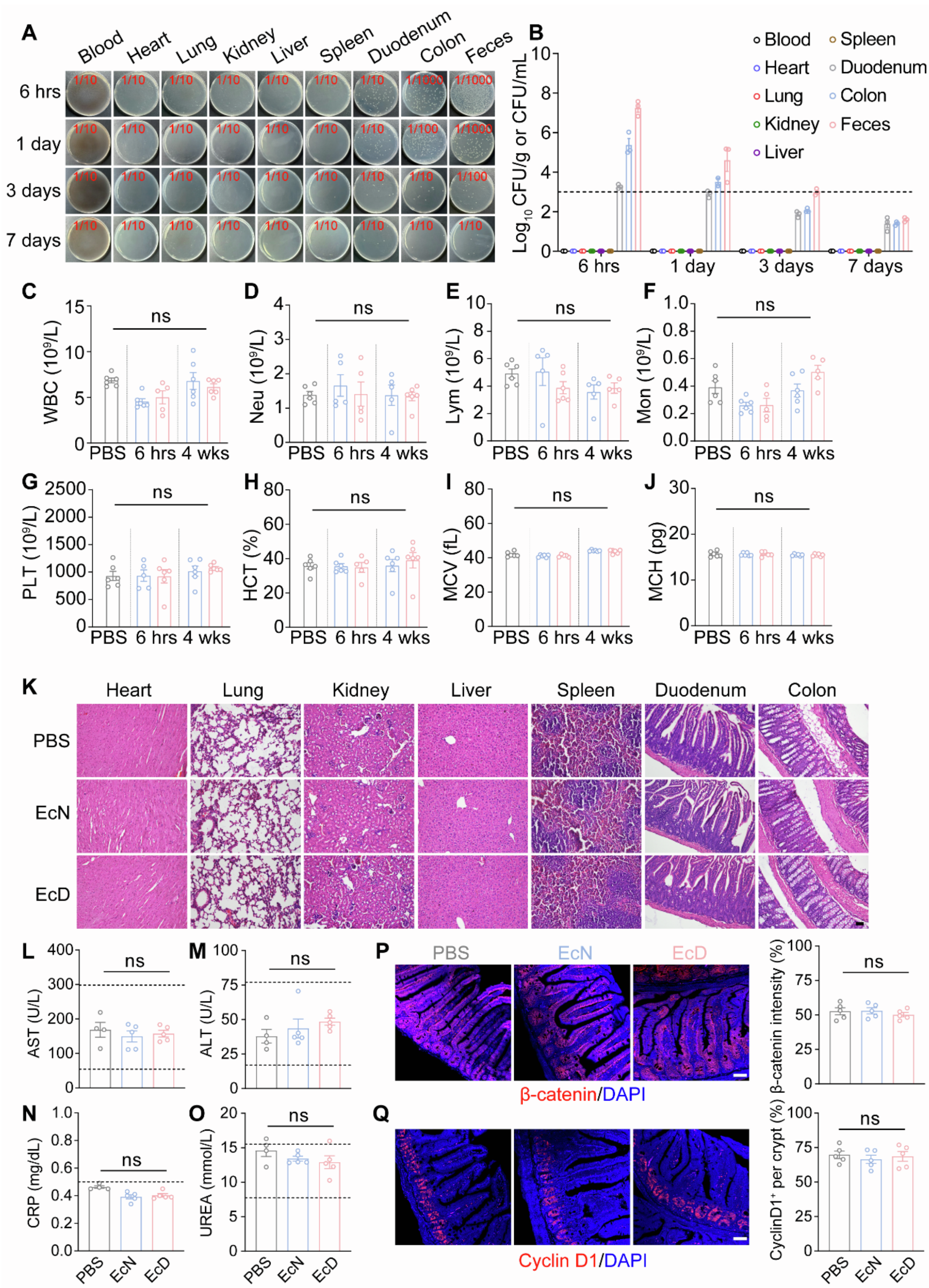

Figure S9. Biosafety assessment of EcD. Related to Figures 4 and 5. (A and B) Representative images (A) and quantification (B) of plate colony counting of blood, heart, lung, kidney, liver, spleen, duodenum, colon tissues and fecal samples, harvested from mice after single oral gavage. (C-J) Routine blood examination of WBC, Neu, Lym, Mon, PLT, HCT, MCV and MCH with long-term administration. WBC, white blood cell; Neu, neutrophils; Lym, lymphocytes; Mon, monocytes; PLT, platelet; HCT, hematocrit; MCV, mean corpuscular volume and MCH, mean

corpuscular. (K) Representative H&E-stained sections of duodenum, colon and other organs (heart, lung, kidney, liver and spleen) post 4 weeks treatments, scale bar = 50 μm. (L-O) Serum levels of AST, ALT, CRP and UREA in wild type mice within 4 weeks bacterial exposure. AST, aspartate aminotransferase; ALT, alanine aminotransferase; CRP, C-reaction protein; UREA, urea. Normal values: AST, 54-298 IU/L; ALT, 17-77 IU/L; CRP, < 0.5mg/dL; UREA, 7.75-15.52 mmol/L. The dashed lines represent the normal ranges. (P and Q) Representative images and quantification of β-catenin (P) and Cyclin D1 (Q) expression in duodenum with 4 weeks bacterial exposure, scale bar = 50 μm. Data are presented as mean ± SEM. Statistical significance determined using an unpaired t test. ns, $P > 0.05$.

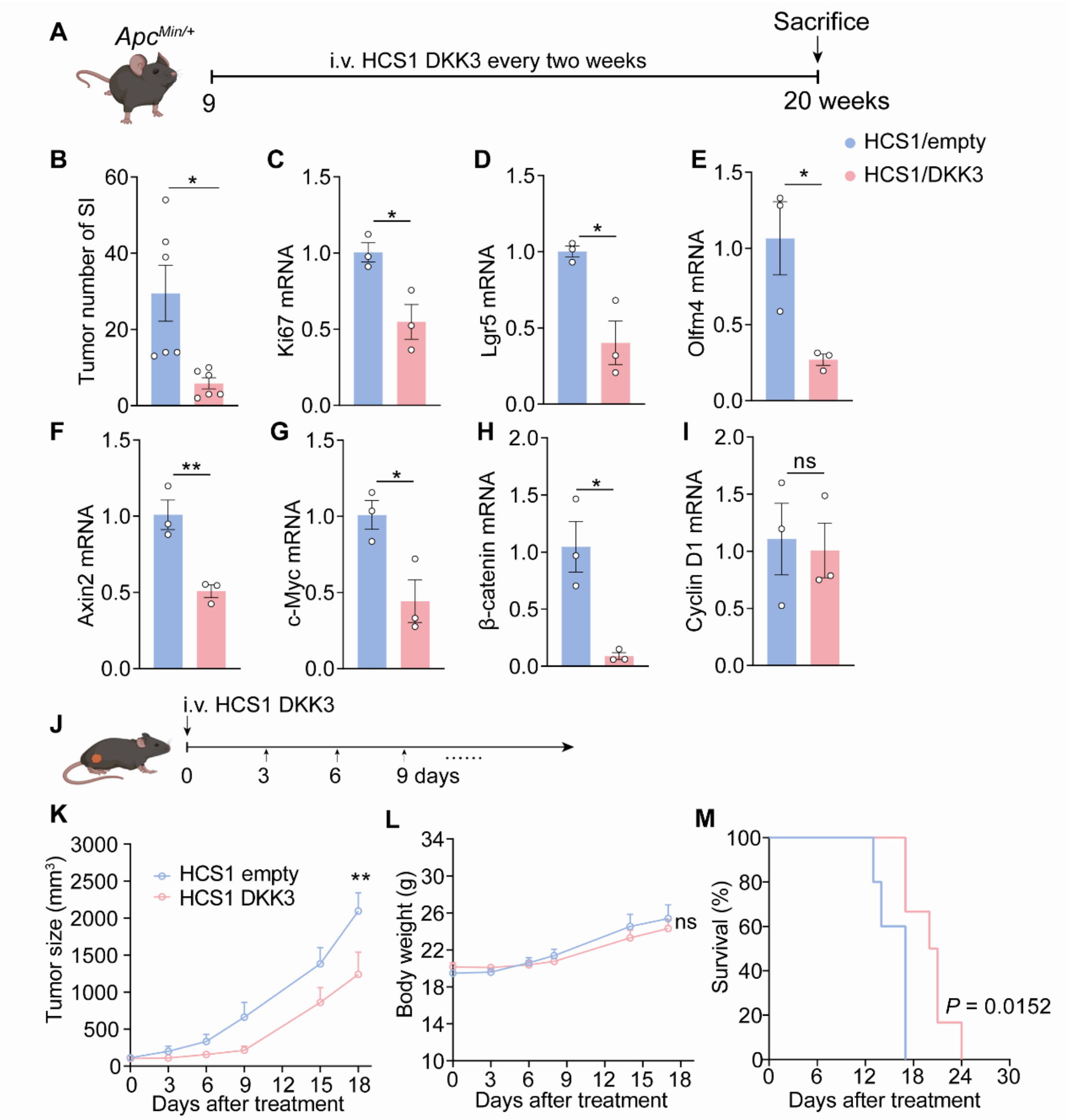

Figure S10. Antitumor effects of HCS1/DKK3 in *Apc*$^{Min/+}$ mice and MC38 models. Related to Figures 4 and 5. (A) Experimental scheme of engineered HCS1 in *Apc*$^{Min/+}$ mice. (B) Tumor numbers in small intestine at the 20th week, n = 6. (C to E) mRNA level of *Ki67* (C), *Lgr5* (D) and *Olfm4* (E) in tumors with HCS1/DKK3-treated. (F to I) mRNA level analysis of *Axin2* (F), *c-Myc* (G), *β-catenin* (H) and *Cyclin D1* (I) in *Apc*$^{Min/+}$ mice tumors with HCS1/DKK3-treated. (J) Experimental scheme of engineered HCS1 in MC38 subcutaneous tumor model. (K to M) Tumor growth (K), body weight change (L) and animal survival (M) of MC38-bearting mice after bacterial injection (n = 5-6). Data are presented as mean ± SEM. Statistical significance determined using an unpaired t test or two-way ANOVA. ns, $P > 0.05$, *$P \leq 0.05$ and ** $P \leq 0.01$.

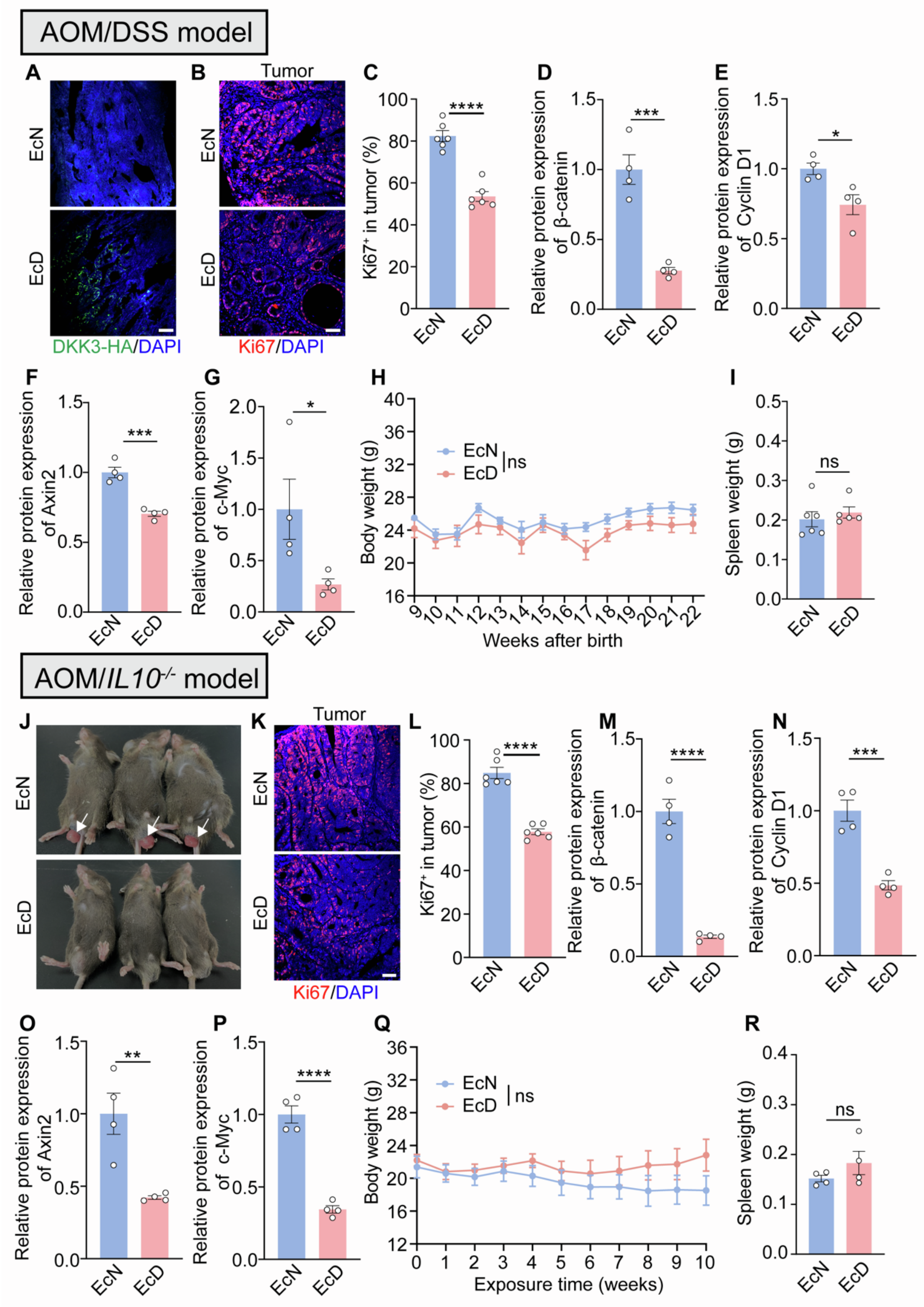

Figure S11. Effects of ISCs proliferation and Wnt signaling with EcD administration in AOM/DSS and AOM/*IL10*$^{-/-}$ models. Related to Figure 6. (A) IF staining image of HA-tagged DKK3 in colon tumor. (B and C) IF staining image of Ki67 in colon tumors and its signal quantification. (D to G) Quantitative analysis for western blot analysis of β-catenin (D), Cyclin D1 (E), Axin2 (F) and c-Myc (G) in AOM/DSS-treated tumors. (H and I) Body weight (H) and spleen weight (I) changes

of AOM/DSS mice with EcD treated. (J) The physical appearance of AOM/*IL10*$^{-/-}$ mice with EcN and EcD exposure. (K and L) IF staining image of Ki67 in colon tumors and its signal quantification. (M to P) Quantitative analysis for western blot analysis of β-catenin (M), Cyclin D1 (N), Axin2 (O) and c-Myc (P) in AOM/*IL10*$^{-/-}$ mice tumors. (Q and R) Body weight (Q) and spleen weight (R) changes of AOM/*IL10*$^{-/-}$ mice with EcD treated. Data are presented as mean ± SEM. Statistical significance determined using an unpaired t test. ns, $P > 0.05$, *$P \leq 0.05$, ** $P \leq 0.01$, *** $P \leq 0.001$ and **** $P \leq 0.0001$.

Table S1. The basic information of the patients and healthy cohort. Related to STAR Methods.

Cohorts	No	Sex	Age	Stage	Sample	Application
CRC patients	1	Male	63	Rectal cancer	Tissue	IF, PDO
	2	Male	72	Colon cancer	Tissue	IF
	3	Male	65	Rectal cancer	Tissue	IF
	4	Male	62	Rectal cancer	Tissue	PDO
	5	Male	76	Colon cancer	Tissue	PDO
	6	Female	63	Colon cancer	Tissue	IF
	7	Female	70	Colon adenoma	Tissue	IF
	8	Female	62	Colon cancer	Tissue	PDX
	9	Male	66	Colon cancer	Stool	Diagnosis
	10	Male	68	Colon cancer	Stool	Diagnosis
	11	Male	58	Rectal cancer	Stool	Diagnosis
	12	Male	62	Colon cancer	Stool	Diagnosis
	13	Male	73	Rectal cancer	Stool	Diagnosis
	14	Female	37	Rectal cancer	Stool	Diagnosis
	15	Male	65	Rectal cancer	Stool	Diagnosis
	16	Male	68	Colon cancer	Stool	Diagnosis
	17	Male	62	Rectal cancer	Stool	Diagnosis
	18	Female	78	Rectal cancer	Stool	Diagnosis
	19	Female	61	Rectal cancer	Stool	Diagnosis
	20	Female	62	Colon cancer	Stool	Diagnosis
IBD patients	1	Male	39	Crohn's Disease	Stool	Diagnosis
	2	Male	55	Ulcerative Colitis	Stool	Diagnosis
	3	Male	28	Crohn's Disease	Stool	Diagnosis
	4	Male	36	Crohn's Disease	Stool	Diagnosis
	5	Male	37	Ulcerative Colitis	Stool	Diagnosis
	6	Male	42	Crohn's Disease	Stool	Diagnosis
	7	Female	40	Crohn's Disease	Stool	Diagnosis
	8	Male	30	Ulcerative Colitis	Stool	Diagnosis
Healthy control	1	Male	37	NA	Stool	Diagnosis
	2	Male	43	NA	Stool	Diagnosis
	3	Female	27	NA	Stool	Diagnosis
	4	Male	40	NA	Stool	Diagnosis
	5	Female	31	NA	Stool	Diagnosis
	6	Male	60	NA	Stool	Diagnosis
	7	Male	36	NA	Stool	Diagnosis
	8	Female	42	NA	Stool	Diagnosis
	9	Male	58	NA	Stool	Diagnosis
	10	Male	25	NA	Stool	Diagnosis
	11	Male	65	NA	Stool	Diagnosis
	12	Female	35	NA	Stool	Diagnosis

NA, not applicable.

Table S2. Summary of the strains used in this study. Related to STAR Methods.

Lable	Bacterial strain	Plasmids	Use(s)
EcO-sfGFP	EcN	pJ23119-speFL-sfGFP	Fluorescence detection and imaging
EcO-Rluc8	EcN	pJ23119-speFL-Rluc8	Bioluminescence imaging
EcN	EcN	pJ23119-empty (backbone)	Drug protein expression control
EcD	EcN	pJ23119-pelB-DKK3-HA	Drug protein expression
HCS1/empty	HCS1	pJ23119-empty (backbone)	Drug protein expression control
HCS1/DKK3	HCS1	pJ23119-pelB-DKK3-HA	Drug protein expression
